# Antioxidant Lignans and Neolignans from Acorus tatarinowii

**DOI:** 10.1038/srep22909

**Published:** 2016-03-10

**Authors:** Yuanyuan Lu, Yongbo Xue, Shenjie Chen, Hucheng Zhu, Jinwen Zhang, Xiao-Nian Li, Jianping Wang, JunJun Liu, Changxing Qi, Guang Du, Yonghui Zhang

**Affiliations:** 1 Hubei Key Laboratory of Natural Medicinal Chemistry and Resource Evaluation, School of Pharmacy, Tongji Medical College, Huazhong University of Science and Technology, Wuhan 430030, Hubei Province, People’s Republic of China; 2 Tongji Hospital Affiliated to Tongji Medical College, Huazhong University of Science and Technology, Wuhan 430030, Hubei Province, People’s Republic of China; 3 State Key Laboratory of Phytochemistry and Plant Resources in West China, Kunming Institute of Botany, Chinese Academy of Sciences, Kunming 650204, People’s Republic of China

## Abstract

Eleven new lignans and neolignans, named acortatarinowins G–N (**1**−**8**), including three pairs of enantiomers (**1a**/**1b**−**3a**/**3b**) and five optically pure lignans and neolignans (**4**−**8**), along with five known analogs (**9**−**14**), were isolated from the rhizomes of *Acorus tatarinowii* Schott. Compounds **1**−**3** were successfully separated by chiral HPLC to afford **1a**/**1b**−**3a**/**3b**. The planar structures of **1**−**8** were elucidated by extensive spectroscopic analyses including HRESIMS and NMR. Their absolute configurations were determined by analyses of single-crystal X-ray diffraction and a modified Mosher’s method, assisted by experimental and calculated electronic circular dichroism (ECD) data. Compounds **1a** and **1b** were rare 7,8′-epoxy-8,7′-oxyneolignane. Compounds **1**−**8** were evaluated for their antioxidant activities using 2,2-diphenyl-1-picrylhydrazyl (DPPH) reducing antioxidant power assay. Compound **6,** exhibiting strong DPPH radical scavenging capacity with IC_50_ value of 16.4 ± 0.22 *μ*g/mL, could interpret the herbal traditional usage.

Reactive oxygen free radical, produced in normal or pathological cell metabolism, is oxygenated to provide energy to fuel biological processes for many living organisms^1^. However, many diseases, such as cirrhosis and arteriosclerosis, rheumatoid arthritis, cancer as well as neurodegenerative diseases associated with aging, are due to the accumulation of oxygen derived free radicals^1^,^2^,^3^.

*Acorus tatarinowii* Schott (family Araceae) is a well-known traditional Chinese medical plant, whose rhizomes are historically used to treat neurodegenerative diseases, such as apoplexy and dementia, amnesia, epilepsy, improvement of learning and memory, especially Alzheimer’s disease (AD)^4^,^5^. AD is a devastating neurodegenerative disease, and no effective treatment is available, affecting more than 35 million people worldwide^6^. Therefore, it is increasingly urgent to search for bioactive compounds on the cure of AD from traditional Chinese medicine.

As part of our continuing efforts to discover bioactive natural products in the medical plant *A. tatarinowii*, a series of enantiomeric lignans and norlignans were previously reported^7^. In our current study, further investigation on chemical constituents of the rhizomes of *A. tatarinowii* in different regions has led to the isolation of eleven new lignans and neolignans (**1a**/**1b**−**3a**/**3b** and **4**−**8**), and six known analogs (**9**−**14**). All of these new isolates were evaluated for their antioxidant activities to interpret the herbal traditional usage and to discover new potential agent for AD. Herein, we report the isolation, structural elucidation, and the absolute configuration determination of all new compounds.

## Results and Discussion

### Structure Elucidation

**Figure 1:**
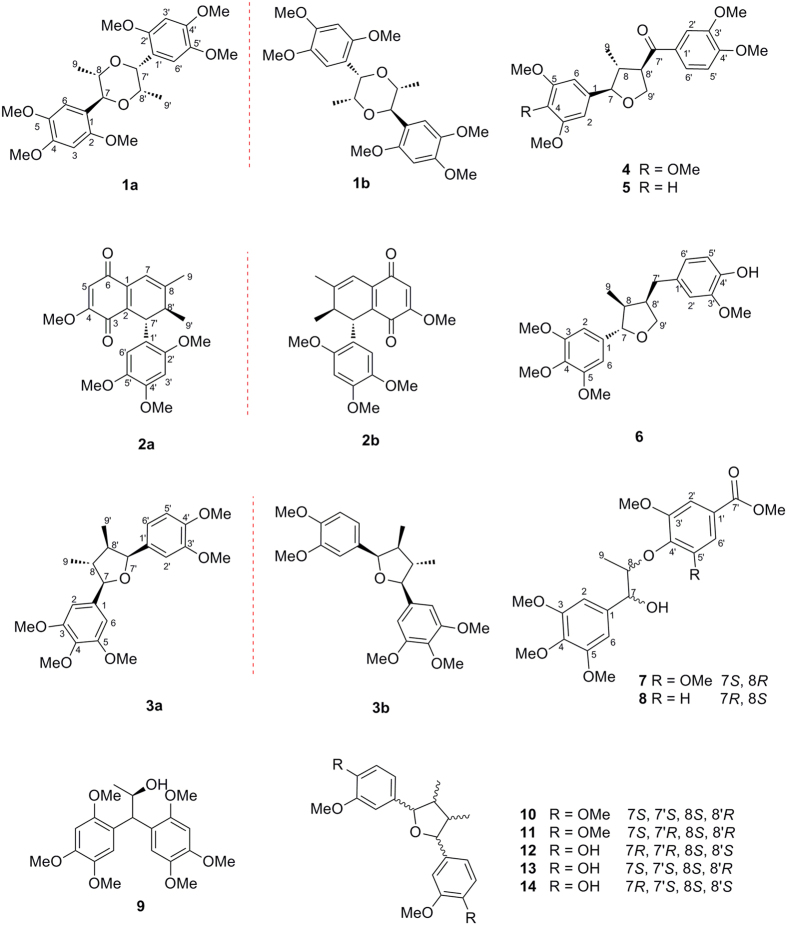
Structures of isolated compounds.

The EtOAc extract of the air-dried rhizomes of *A. tatarinowii* was subjected to a series of chromatographic separations, to yield eleven new (**1a/1b**−**3a**/**3b** and **4**−**8**) and six known (**9**−**14**) neolignans and lignans (Fig. 1). The structures of the known compounds (**9**−**14**) were identified as tatarinoid C (**9**)^8^, (+)-veraguensin (**10**)^9^, (−)-galgravin (**11**)^10^, and (+)-saucernetindiol (**12**)^11^, (−)-machilin-I (**13**)^12^ and (+)-verrucosn (**14**)^11^, respectively, by comparing their NMR data with those reported in literatures.

(±)-Acortatarinowin G (**1a/1b**), obtained as block crystals in MeOH (mp 186–187 °C), had a molecular formula of C_24_H_32_O_8_ based on ^13^C NMR data and a sodiated molecular ion in positive HRESIMS at *m/z* 471.1977 [M + Na]^+^ (calcd for C_24_H_32_NaO_8_, 471.1995). The ^1^H NMR (Table 1) data of **1** showed signals for two 1,2,4,5-tetrasubstituted phenyl groups (*δ*_H_ 7.08, 1H, s; 7.07, 1H, s; 6.51, 1H, s; and 6.49, 1H, s), four oxygenated methines (*δ*_H_ 5.25, 1H, d, *J* = 3.2 Hz; *δ*_H_ 5.02, 1H, d, *J* = 9.3 Hz; *δ*_H_ 4.34, 1H, m; and *δ*_H_ 3.76, 1H, d, m), six methoxy groups (*δ*_H_ 3.90, 2*3.89, 3.88, 3.83, and 3.81), and two methyl signals (*δ*_H_ 1.12, 3H, *J* = 6.8 Hz and *δ*_H_ 1.08, 3H, *J* = 6.3 Hz). The ^13^C NMR and DEPT (Table 2) of **1** revealed 24 carbons, which were resolved into 12 aromatic carbons, four oxygenated methine carbons, six methoxy groups, and two methyl carbons. The spectroscopic data suggested that **1** was a 7,8-dioxane neolignan which was very similar to verimol H except that two 1,4-dimethoxyphenyl moieties in verimol H were replaced by two 1,2,4,5-tetramethoxyphenyl groups in **1**^13^. This conclusion was supported by the HMBC correlations from H-7 (*δ*_H_ 5.02) to C-1 (*δ*_C_ 119.8), C-2 (*δ*_C_ 151.8), C-6 (*δ*_C_ 111.7), C-8 (*δ*_C_ 78.8), C-9 (*δ*_C_ 17.1), and C-8′ (*δ*_C_ 70.8), and from H-7′ (*δ*_H_ 5.25) to C-1′ (*δ*_C_ 119.5), C-2′ (*δ*_C_ 150.0), C-6′ (*δ*_C_ 111.2), C-8′ (*δ*_C_ 70.8), C-9′ (*δ*_C_ 12.3), and C-8 (*δ*_C_ 78.8). In addition, the HMBC correlations of OMe-2/C-2, OMe-4/C-4, OMe-5/C-5, OMe-2′/C-2′, OMe-4′/C-4′ and OMe-5′/C-5′, verified that compound **1** was 2,4,5, 2′,4′,5′-hexamethoxy-7,8′-epoxy-8,7′-oxyneolignane.

**Figure 2:**
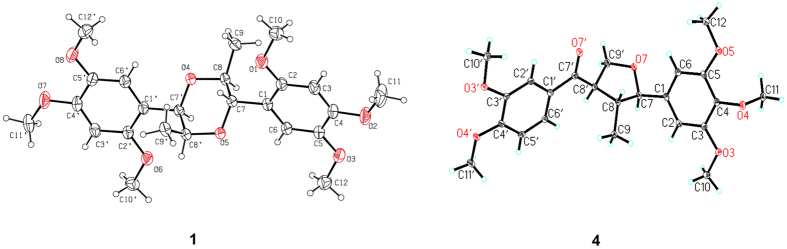
ORTEP drawing of compounds 1 and 4.

**Figure 3:**
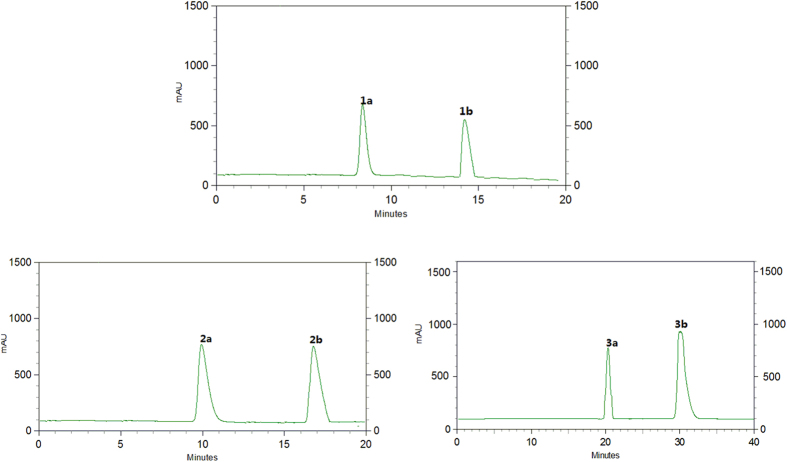
Chiral HPLC separation profiles of compounds 1a/1b−3a/3b.

**Figure 4:**
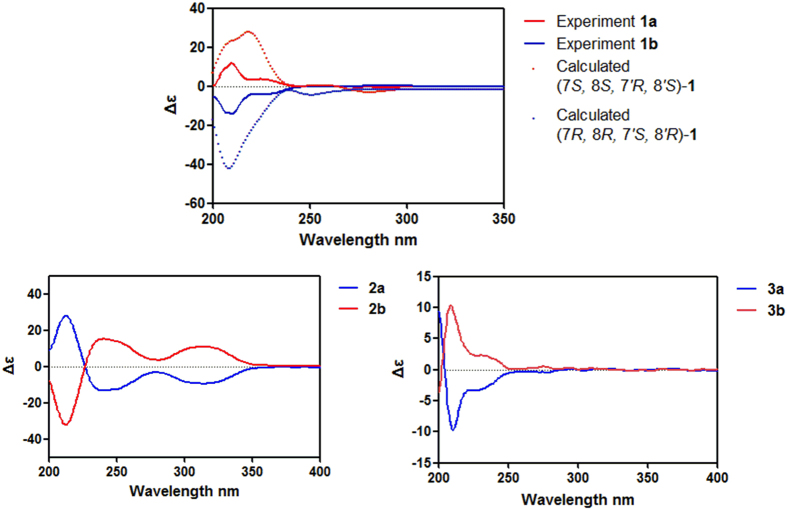
The experimental ECD spectra of 1a/1b−3a/3b, and the calculated ECD spectra for 1a/1b.

The relative configuration of compound **1** was determined through a NOESY experiment and the coupling constants, and further confirmed by X-ray crystallographic analyses. The large coupling constant of H-7/H-8 (*J*_7,8_ = 9.3 Hz) suggested a *trans*-relationship between H-7 and H-8, and the small coupling constant of H-7′/H-8′ (*J*_7′,8′_ = 3.2 Hz) indicated H-7′ and H-8′ were in a *cis* position. The key cross-peak of H-7′/H-8 in the NOESY spectrum indicated the relative configuration of acortatarinowin G was (7R*, 8R*, 7′S*, 8′R*). Fortunately, crystals of **1** were obtained from MeOH, and a single X-ray diffraction experiment was performed using Cu K*α* radiation (Fig. 2), which supported the above conclusion. Surprisingly, the crystal of **1** had the centrosymmetric space group *P-*2*yc*, indicating its racemic nature^13^,^14^, which was supported by the absence of optical rotation and any Cotton effects in the ECD spectrum. Subsequent chiral resolution led to the isolation of **1a** and **1b** (Fig. 3, **1a:1b** = 1:1) with opposite specific rotation and mirror imaged Cotton effects in their ECD spectrum (Fig. 4). An 8*S* configuration for **1a** was established by a positive Cotton effect around 230 nm in the ECD spectrum (Fig. 4)^15^,^16^,^17^. Furthermore, the experiment ECD spectrum of **1a**/**1b** matched well with calculated ECD spectrum of (7*S,* 8*S*, 7′*R*, 8′*S*)-**1** and (7*R*, 8*R*, 7′*S*, 8′*R*)-**1** (Fig. 4), respectively. Thus, the absolute configurations of **1a** and **1b** were established as shown (Fig. 1).

(±)-Acortatarinowin H (**2a/2b**), obtained as brown-red amorphous powder, had a molecular formula of C_22_H_24_O_6_ as revealed by HRESIMS spectrum (*m/z* 407.1493 [M + Na]^+^) and ^13^C NMR data. The ^1^H NMR data showed signals (Table 1) attributable to two methyl groups, two methine protons, four methoxy protons, two olefinic protons, and two aromatic protons. The ^13^C-NMR spectrum showed the presence of 22 carbon signals, corresponding to two methyl carbons, two methine carbons, four methoxy groups, 12 olefinic carbons and two conjugated carbonyl carbons, which resembled those of 7′-(2′,4′,5′-trimethoxyphenyl)-4-methoxy-8,8′-dimethyl-2,5 quinone^18^. A side-by-side comparison of the ^1^H and ^13^C NMR data of **1** with those of 7′-(2′,4′,5′-trimethoxyphenyl)-4-methoxy-8,8′-dimethyl-2,5-quinone revealed that the small differences were the absence of two olefinic carbons and the appearance of two methine carbons in **2**. Based on the aforementioned evidence, **2** was determined to be 4,2′,4′,5′-tetramethoxy-7-ene-3,6-dione-2,7′-cyclolignane, which was further supported by the ^1^H−^1^H COSY correlations of H-7′/H-8′ and H-8′/H-9′, and the key HMBC correlations from H-7′ (*δ*_H_ 4.44) to C-1′ (*δ*_C_ 121.4), C-2′ (*δ*_C_ 151.1), C-6′ (*δ*_C_ 112.2), C-8′ (*δ*_C_ 41.5), C-9′ (*δ*_C_ 19.5), C-1 (*δ*_C_ 137.3), C-3 (*δ*_C_ 181.5), C-2 (*δ*_C_ 132.5), and C-8 (*δ*_C_ 153.3).

The relative configurations at C-7′ and C-8′ of the core skeleton of **2** were established by analyses of coupling constants aided by molecular models generated by Chem3D. According to those models, H-7′ and H-8′ in a *trans* position matched well with the coupling constant (*J*_7′,8′_ = 0 Hz), which was supported by a single peak at *δ*_H_ 4.44 (H-7′) and a quartet peak at *δ*_H_ 2.31 (H-8′). The absence of any Cotton effects in the ECD spectrum and optical rotation revealed compound **2** was a pair of racemic mixture as well. These findings were supported by chiral HPLC analyses of **2** (Fig. 3). Compounds **2a** and **2b** were successfully obtained using a Daicel IC column, showing opposite specific rotations (**2a**: [α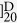 +14; 2**b**: [*α*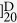 −14) and opposite Cotton effects (Fig. 4) [**2a**: 213 (Δ*ε* +26.05), 243 (Δ*ε*−12.82), 315 (Δ*ε*−9.33); **2b**: 213 (Δ*ε*−27.01), 243 (Δ*ε* +13.02), 315 (Δ*ε* +10.31)].A negative Cotton effect around 315 nm indicated an 8′*S* for **2a**^19^,^20^. Therefore, the absolute configurations of **2a** and **2b** were determined as 7′*R*, 8′*S* and 7′*S*, 8′*R*, respectively.

(±)-Acortatarinowin I (**3a/3b**), obtained as a light yellow gum, its NMR data and positive optical rotation were the same as those of 3,4,5,3′,4′-pentamethoxy-7,7′-epoxylignan reported in the literature with absolute configuration not determined^21^. Based on our previous research^7^, compound **3** was subjected to a Daicel IC column to check its partial racemic nature, and (−)-**3a** and (+)-**3b** (Fig. 3, **3a**:**3b** ≈1:3) were successfully obtained, showing typical antipodal ECD curves (Fig. 4) and specific rotations of opposite sign. The absolute configuration for **3b** was determined as 7*S*,8*S*,7′*R*,8′*S* by comparing the ECD curve of **3b** with that of closely related compound hydroxyvera^22^. Hence, that of **3a** was determined as 7*R*,8*R*,7′*S*,8′*R* (Fig. 1).

Acortatarinowin J (**4**), was isolated as colourless crystals (MeOH) with mp 158–159 °C. The molecular formula of **4** was determined as C_23_H_28_O_7_ according to ^13^C NMR data and HREIMS data *m/z* 439.1718 [M + Na]^+^ (calcd for C_23_H_28_NaO_7_, 439.1733). The IR spectrum indicated the presence of conjugated carbonyl group (1674 cm^–1^) and aromatic rings (1594 and 1515 cm^–1^). The NMR data (Tables 1 and 2) showed signals attributable to a 1,3,4,5-tetrasubstituted and a 1,3,4-trisubstituted aromatic moiety, five methoxy groups, three methines including an oxygenated methine, an oxygenated methylene unit, a methyl group and a conjugated carbonyl group. These findings indicated that compound **4** is a 7,9′-epoxylignan similar to magnone B^23^. Comparison of the NMR data indicated that the only small difference between **4** and magnone B was that the hydroxymethyl group (C-8) in magnone B was reduced to a methyl group. This deduction was supported by the HMBC correlations from H-7 (*δ*_H_ 4.38) to C-1 (*δ*_C_ 136.0), C-2,6 (*δ*_C_ 103.7), C-8 (*δ*_C_ 45.9), C-9 (*δ*_C_ 15.4), C-8′ (*δ*_C_ 54.4), and C-9′ (*δ*_C_ 70.8), from H-8 (*δ*_H_ 2.62) to C-1(*δ*_C_ 136.0), C-7(*δ*_C_ 89.2), C-8′ (*δ*_C_ 54.4), and C-7′ (*δ*_C_ 197.6), and from H-2′/6′ (*δ*_H_ 7.55/7.56) to C-7′ (*δ*_C_ 197.6). In addition, the HMBC correlations for OMe-3/C-3, OMe-4/C-4, OMe-5/C-5, OMe-3′/C-3′, and OMe-4′/C-4′ verified the location of these substituents. Thus, the structure of **4** was determined as 3,3′,4,4′, 5-pentamethoxy-7,9′-epoxylignan-7′-one.

The relative configuration of **4** was established through a NOESY experiment and coupling constant. The big coupling constant (*J* = 9.5 Hz) between H-7 and H-8 indicated the t*rans*-orientation, and the strong NOESY correlation of H-7 and H-8′ verified they were in a *cis*-orientation. Therefore, the 2D structure of **4** was determined as (7R*, 8R*, 8′R*)-3,3′,4,4′,5-pentamethoxy-7,9′-epoxylignan-7′-one. Fortunately, the crystals of **4** were obtained from MeOH, and the absolute configuration of acortatarinowin J was finally elucidated as 7*R*,8*R*,8′*R* on the basis of single crystal X-ray diffraction (Fig. 2).

Acortatarinowin K (**5**), obtained as white amorphous powder, possesses the molecular formula C_22_H_26_O_6_, as determined from a sodiated molecular ion in the positive HRESIMS at *m/z* 387.1797 [M + H]^+^ (calcd for C_22_H_27_O_6_, 387.1808) and its NMR data. A side-by-side comparison of the ^13^C NMR data of **5** with those of **4** revealed that **5** was closely related to **4** except the absence of a methoxy group. Correspondingly, the signals attributable to a symmetrical 1,3,4,5-tetrasubstituted aromatic moiety in **4** was replaced by those of a 1,3,5-trisubstituted aromatic moiety in the ^1^H NMR spectrum. Therefore, compound **5** was surmised to be 3,3′,4′, 5-tetramethoxy-7,9′-epoxylignan-7′-one. This deduction was confirmed by 2D NMR data analyses. The big coupling constant (*J*_7,8_ = 9.5 Hz) and key NOESY correlation from H-7 to H-8′ indicated the relative configuration of **5** was the same as **4**. Furthermore, the experiment ECD spectrum of **5** matched well with that of **4** (Supporting Information, SI, Figs S37 and S47). Thus, the absolute configuration of **5** was determined as 7*R*,8*R*,8′*R*.

Acortatarinowin L (**6**), had the molecular formula C_22_H_28_O_6_, determined on the basis of a sodiated molecular ion in the positive HRESIMS at *m/z* 411.1774 [M + Na]^+^ (calcd for C_22_H_28_NaO_6_, 411.1784) and its NMR data. A comparison of the NMR data (Tables 1 and 2) between **6** and **4** indicated that they differed in the presence of a methylene unit and the absence of a conjugated carbonyl and a methoxy group in **6**. Thus, compound **6** was determined as 4′-hydroxy-3,3′,4,5-tetramethoxy-7,9′-epoxylignan. The NOESY correlations from H-9 to H-7′ and H-7 revealed H-7 was oriented opposite to H-8 and H-8′. Thus, the relative configuration of **6** was determined as 7R*, 8R*, 8′S*. Compound **6** displayed a coupled Cotton effect, negative at 235 nm and positive at 215 nm (SI, Fig. S57), indicating exciton coupling in the π→π* transition of the phenyl chromophores^24^. The positive chirality ([α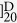 +18) revealed the 7*R*, 8*R*, 8′*S* configuration for **6** on the basis of the ECD exciton chirality rule^17^,^24^,^25^. Therefore, the absolute configuration of **6** was assigned as 7*R*, 8*R*, 8′*S*.

**Figure 5:**
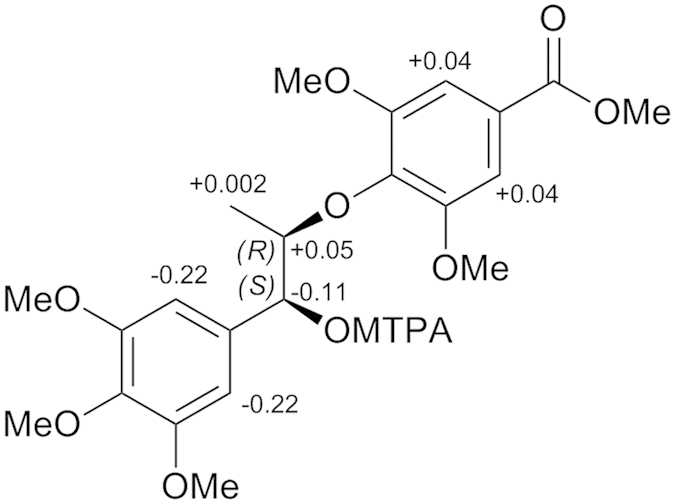
Δ*δ*_H(*S*−*R*)_ values (in ppm) for the MTPA esters of 7.

Acortatarinowin M (**7**) was obtained as a light yellow gum. The HRESIMS ion at *m/z* 459.1619 [M + Na]^+^ (calcd for C_22_H_28_NaO_9_, 459.1631) and the NMR data established its molecular formula as C_22_H_28_O_9_. The NMR data (Tables 1 and 2) displayed signals for two symmetric tetrasubstituted aromatic moieties, six methoxy groups, two oxygenated methines, a methyl, and a conjugated carbonyl. These findings indicated that compound **7** was an 8-*O*-4′ type neolignan and was similar to acortatarinowin C with the difference in the presence of an methoxy group in **7**^7^. The key HMBC correlation from H-2′**/**H**-**6′ to C-7′ revealed compound **7** was 5′-methoxy derivative of acortatarinowin C. The absolute configuration at C-7 was determined as 7*S* by modified Mosher’s method (Fig. 5), and C-8 was *R* due to a negative Cotton effect around 237 nm^15^,^16^,^17^.

Acortatarinowin N (**8**) was obtained as a light yellow gum. The NMR data and an HRESIMS [M + Na]^+^ ion at *m/z* 429.1464 (calcd for C_21_H_26_NaO_8_, 429.1525) allowed the assignment of the molecular formula of C_21_H_26_O_8_ the same as acortatarinowin C^7^. A side-by-side comparison of the ^1^H and ^13^C NMR data of **8** with those of acortatarinowin C indicated there were only small changes in chemical shifts from H-7/C-7 to H-9/C-9 [(*δ*_H_ 4.73 (1H, d, *J* = 4.9 Hz, H-7), *δ*_C_ 77.1 (C-7); 4.65 (1H, dq, *J* = 6.1, 4.9 Hz, H-8), 80.0 (C-8); and 1.31 (3H, d, *J* = 6.1 Hz, H-9,15.6 (C-9 in **8**); (*δ*_H_ 4.75 (1H, d, *J* = 5.4 Hz, H-7), *δ*_C_ 77.1 (C-7); 4.69 (1H, dq, *J* = 6.2, 5.4 Hz, H-8), 80.0 (C-8); 1.17 (3H, d, *J* = 6.2 Hz, H-9,16.1 (C-9) in acortatarinowin C)]. On the basis of these observations, along with 2D NMR analyses, compound **8** was deduced to be the stereoisomer of acortatarinowin C with an *erythro* configuration (7R*, 8S*) due to its small coupling constant (*J*_7,8_ = 4.9 Hz). The positive ECD Cotton effect near 238 nm of **8** agreed well with that of (−)-acortatarinowin B^7^ and its structurally related neolignan compound (7*R*, 8*S*)-balanophonin^25^,^26^. Thus, the absolute configuration of acortatarinowin N was determined as 7*R*,8*S.*

### Antioxidant Activity Evaluation

The abilities of the compounds **1**−**8** to scavenge DPPH radical were evaluated from 6.25 to 100 *μ*g/mL. Trolox (vitamin E) was used as positive control for antioxidant activity (Table 3). Among the compounds tested, compound **6** exhibited the most potent antioxidant activity with IC_50_ value of 16.437 ± 0.22 *μ*g/mL, whereas the other compounds did not exhibit any activity with the maximum concentration 100 *μ*g/mL. By comparing the structures of all the compounds tested, it was concluded that the presence of phenolic hydroxyl functionality was required for the antioxidant activity of these lignans and neolignans.

## Conclusion

In this study, eleven new lignans and neolignans, including three pairs of enantiomers **1a**/**1b**–**3a/3b,** were isolated from the rhizomes of *A. tatarinowii*. Compound**s 1a** and **1b** were rare 7,8′-epoxy-8,7′-oxyneolignanes. The absolute configurations of the two 7,8′-epoxy-8,7′-oxyneolignanes (**1a/1b**) were determined for the first time by comparison of their experimental and calculated ECD spectrum. Biologically, the antioxidant activities of the new isolates were evaluated. Compound **6** possessing a phenolic hydroxyl moiety exhibited the most potent antioxidant activity, whereas the other compounds, containing no phenolic hydroxyl groups in their structures, did not show activity in DPPH assay. Currently, pathogenetic and biochemical studies have consistently suggested oxidative stress have played an important role in AD pathogenesis^27^, and many studies have focused on searching therapeutic agents for AD through inhibiting reactive oxygen species production^28^. Thus, the results of the study could elucidate the bioactivity of *A. tatarinowii* in some extent. Further investigations on *in vivo* animal experiments and structure-function relationship for developing more excellent agent for AD are performing.

## Methods

### General experimental procedures

Melting points were measured using a Beijing Tech X-5 micro-melting point apparatus and were uncorrected. UV, FT-IR, and ECD spectra were obtained from a Varian Cary 50, a Bruker Vertex 70, and a JASCO-810 spectrometer, respectively. Optical rotations were measured in MeOH/CH_2_Cl_2_ on a Perkin-Elmer 341 polarimeter equipped with a 1 dm 75 microcell and a sodium lamp (589 nm). 1D and 2D NMR spectra were acquired on a Bruker AM-400 spectrometer and the ^1^H and ^13^C NMR chemical shifts were referenced to the solvent or solvent impurity peaks for methanol-*d*_4_ (*δ*_H_ 3.31 and *δ*_C_ 49.0) and CDCl_3_ (*δ*_H_ 7.26 and *δ*_C_ 77.0). High-resolution electrospray ionization mass spectra (HRESIMS) were acquired in the positive ion mode with a Thermo Fisher LC-LTQ-Orbitrap XL spectrometer. Column chromatography was performed using silica gel (100–200 and 200–300 mesh; Qingdao Marine Chemical Inc., China), octadecylsilyl (ODS, 50 *μ*m, YMC Co. Ltd., Japan), and Sephadex LH-20 (40–70 *μ*m, Amersham Pharmacia Biotech AB, Uppsala, Sweden, Sweden). The crystallographic data were acquired on a Bruker SMART APEX-II CCD diffractometer equipped with graphite-monochromatised Cu Kα radiation (*λ* = 1.54178 Å). Semi-preparative HPLC was carried out on an Agilent 1200 quaternary system with a UV detector or on a Dionex HPLC system equipped with an Ultimate 3000 pump, an Ultimate 3000 autosampler injector, and an Ultimate 3000 diode array detector (DAD) using a reversed-phased C_18_ column (5 *μ*m, 10 × 250 mm) at a flow rate of 2.5 mL/min. Thin-layer chromatography (TLC) was performed with silica gel 60 F_254_ (Yantai Chemical Industry Research Institute) and RP-C_18_ F_254_ plates (Merck, Germany).

### Plant material

The rhizomes of *A. tatarinowii* were collected at Jiujiang, Jiangxi Province, P. R. China in September 2013, and were identified by one of the authors, Prof. J. P. Wang. A voucher specimen (No. 2013–0916A) was deposited with the Herbarium of Materia Medica, Faculty of Pharmacy, Tongji Medical College of Huazhong University of Science and Technology, China.

### Extraction and isolation

The air-dried rhizomes of *A. tatarinowii* (40 kg) were extracted with 95% EtOH three times at room temperature, and the extract was partitioned with petroleum ether, EtOAc, and *n*-BuOH against water. The EtOAc fraction (400.0 g) was separated by chromatography to obtain six fractions (Fr.1–6) using silica gel CC eluting with a gradient of petroleum ether–acetone (20:1–1:1). Fr. 2 was further purified by column chromatography (silica gel CC, 10 × 100 cm), eluting with a gradient of petroleum ether–EtOAc (15:1–1:1), to yield five fractions (Fr.2.1–2.5). Fr. 2.3 were subjected to an ODS column (MeOH–H_2_O, 30:70–70:30) and a Sephadex LH-20 (CH_2_Cl_2_−MeOH, 1:1) column to yield three mixtures (A–C). Mixture A was then purified by semi-preparative HPLC (CH_3_CN-H_2_O, 45:55) to obtain compound **1** (*t*_*R*_ 56.0 min, 10.0 mg). Compounds **9** (19.5 mg) and **10** (12.6 mg) were got from mixture B using an RP C-_18_ column eluting with MeOH–H_2_O (65:35), and compound **11** (5 mg) was obtained from Mixture C. Fr.2.4 was separated by repeated silica gel CC eluted with a petroleum ether–EtOAc gradient and Sephadex LH-20 (CH_2_Cl_2_−MeOH, 1:1) to yield two mixtures (D and F). Compounds **2** (*t*_*R*_ 60.0 min, 8.2 mg, CH_3_CN-H_2_O, 50:50) and **3** (*t*_*R*_ 90.0 min, 6.0 mg, CH_3_CN-H_2_O, 45:55) were purified from mixtures D and F by semi-preparative HPLC, respectively. Compound **1** (**1a**/**1b**) was further purified by chiral HPLC eluting with EtOH to give **1a** (*t*_*R*_ 7.0 min, 2.0 mg) and **1b** (*t*_*R*_ 11.5 min, 2.0 mg). Compounds **2a** (*t*_*R*_ 10.0 min, 2.8 mg), **2b** (*t*_*R*_ 17.0 min, 2.8 mg), **3a** (*t*_*R*_ 20.0 min, 1.5 mg), and **3b** (*t*_*R*_ 30.0 min, 4.0 mg) were successfully separated using the same IC column as **1** eluting with EtOH–*n*-hexane 15:85 and 25:75, respectively. Fr. 4 was subjected to a silica gel again eluting with petroleum ether–EtOAc (10:1–3:1) to yield four subfractions, named Fr.4.1–4.4. Fr.4.2 and Fr.4.3 were further partitioned with ODS column (MeOH–H_2_O, 30:70–70:30) and Sephadex LH-20 (CH_2_Cl_2_−MeOH, 1:1) to obtain four subfractions, named Fr.4.2.1–4.2.2 and Fr.4.3.1–4.3.2, respectively. Compounds **7** (*t*_*R*_ 49.0 min, 4.0 mg) and a mixture of **4** and **5** (*t*_*R*_ 44.0 min, 27.0 mg) were separated by semi-preparative HPLC (MeOH–H_2_O, 55:45) equipped with an RP-C_18_ column from Fr.4.2.1, and compounds **4** and **5** were finally purified by semi-preparative HPLC eluting with CH_3_CN-H_2_O (45:55). Compound **8** (*t*_*R*_ 63.5 min, 6.0 mg) was obtained from Fr.4.2.2 by semi-preparative HPLC (MeOH–H_2_O, 50:50). Fr.4.3.1 was subjected to an RP-C_18_ column to obtain compound **6** (*t*_*R*_ 87.4 min, 4.0 mg) and a mixture of **12**–**14** (*t*_*R*_ 74.0 min, 15.0 mg) eluting with CH_3_CN-H_2_O (40:60). Compounds **12**–**14** were purified by repeated semi-preparative HPLC equipped with an RP C_18_ column and a SiO_2_ column, respectively.

#### (±)-Acortatarinowin G (**1**)

Colorless block crystals (MeOH), mp 186*–*187 °C; 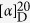 0 (*c* 0.1, MeOH); UV (MeOH) *λ*_max_ (log *ε*) 205 (4.91), 230 (4.41), 291 (4.13) nm; IR (KBr) *ν*_max_ 1612, 1511, 1465, 1399, 1317, 1206, 1130, 1086, 1034, 862, 781 cm^–1^; ^1^H NMR (CDCl_3_, 400 MHz) data, see Table 1; ^13^C NMR (CDCl_3_, 100 MHz) data, see Table 2; (+)-HREIMS *m/z* 471.1977 [M + Na]^+^ (calcd for C_24_H_32_NaO_8_, 471.1995).

#### (+)-Acortatarinowin G (**1a**)

White amporous powder; 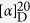 +28 (*c* 0.1, MeOH); ECD (MeOH) 210 (Δ*ε* +13.03), 230 (Δ*ε* +3.33), 260 (Δ*ε* +0.52).

#### (−)-Acortatarinowin G (**1b**)

White amporous powder; 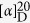 –28 (*c* 0.1, MeOH); ECD (MeOH) 210 (Δ*ε*−13.51), 230 (Δ*ε*−3.53), 260 (Δ*ε*−0.54).

#### (±)-Acortatarinowin H (**2**)

Brown-red amorphous powder, 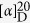 0 (*c* = 0.2, CH_2_Cl_2_); UV (MeOH) *λ*_max_ (log *ε*) = 208 (4.21), 243 (3.83), 290 (3.43), and 315 (3.42) nm; IR *ν*_max_ =1650, 1573, 1509, 1456, 1299, 1243, 1218, 1036, 843, 810 cm^−1^; ^1^H NMR (CDCl_3_, 400 MHz) data, see Table 1; ^13^C NMR (CDCl_3_, 100 MHz) data, see Table 2; HRESIMS *m/z* 407.1493 [M + Na]^+^ (calcd for C_22_H_24_NaO_6_, 407.1471).

#### (+)-Acortatarinowin H (**2a**)

Brown-red amorphous powder; 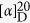 +14 (*c* 0.1, CH_2_Cl_2_); ECD (MeOH) 213(Δ*ε*+26.05), 243 (Δ*ε*−12.82), 315 (Δ*ε*−9.33).

#### (−)-Acortatarinowin H (**2b**)

Brown-red amorphous powder; 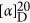 –14 (*c* 0.1, CH_2_Cl_2_); ECD (MeOH) 213 (Δ*ε*−27.01), 243 (Δ*ε* +13.02), 315 (Δ*ε* +10.31).

#### (±)-Acortatarinowin I (**3**)

Light yellow gum, 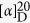 +13 (*c* = 0.4, MeOH); UV (MeOH) *λ*_max_ (log *ε*) = 207 (4.72), 232 (4.51), and 278 (4.04) nm; IR *ν*_max_ = 927, 1590, 1509, 1454, 1417, 1381, 1328, 1233, 1125, 1027, 812, 766, and 713 cm^−1^; ^1^H NMR (methanol-*d*_4_, 400 MHz) data, see Table 1; ^13^C NMR (methanol-*d*_4_, 100 MHz) data, see Table 2; HRESIMS *m/z* 425.1958 [M + Na]^+^ (calcd for C_23_H_30_NaO_6_, 425.1940).

#### (−)-Acortatarinowin I (**3a**)

Light yellow gum; 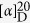 –32 (*c* 0.1, MeOH); ECD (MeOH) 210 (Δ*ε*−9.71), 233 (Δ*ε*−3.10);

#### (+)-Acortatarinowin I (**3b**)

light yellow gum; 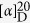 +32 (*c* 0.3, MeOH); ECD (MeOH) 210 (Δ*ε* +10.32), 230 (Δ*ε* +2.55).

#### Acortatarinowin J (**4**)

Colorless block crystals, mp 158*–*159 °C; 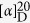 −1.4 (*c* = 0.4, CH_2_Cl_2_); UV (MeOH) *λ*_max_ (log *ε*) = 207 (5.19), 231(4.60), 278 (4.11), and 303 (3.95) nm; ECD (MeOH) *λ*_max_ (Δ*ε*) 210 (−3.51), 237 (+1.32), 275 (−0.80), 305 (−0.92) nm; IR *ν*_max_ = 2934, 2030, 1674, 1594, 1514, 1486, 1419, 1266, 1127, 1023, 829, and 766 cm^−1^; ^1^H NMR (CDCl_3_, 400 MHz) data, see Table 1; ^13^C NMR (CDCl_3_, 100 MHz) data, see Table 2; HRESIMS *m/z* 439.1718 [M + Na]^+^ (calcd for C_23_H_28_NaO_7_, 439.1733).

#### Acortatarinowin K (**5**)

White amorphous powder, 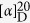 −1.2 (*c* = 0.1, MeOH); UV (MeOH) *λ*_max_ (log *ε*) = 205 (4.45), 231(4.24), 278 (3.93), and 308 (3.77) nm; ECD (MeOH) *λ*_max_ (Δ*ε*) 210 (−1.05), 233 (+0.71), 266 (−0.20), 303 (−0.48) nm; IR *ν*_max_ = 2934, 2031, 1673, 1633, 1518, 1446, 1419, 1299, 1166, 1022, 813, and 765 cm^−1^; ^1^H NMR (methanol-*d*_4_, 400 MHz) data, see Table 1; ^13^C NMR (methanol-*d*_4_, 100 MHz) data, see Table 2; HRESIMS *m/z* 387.1797 [M + H]^+^ (calcd for C_22_H_27_O_6_, 387.1808).

#### Acortatarinowin L (**6**)

Light yellow gum, 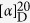 +18 (*c* = 0.1, MeOH); UV (MeOH) *λ*_max_ (log *ε*) = 205 (4.81), 230 (4.15), and 280 (3.53) nm; ECD (MeOH) *λ*_max_ (Δ*ε*) 210 (+3.92), 228 (+0.81), 275 (+0.44) nm; IR *ν*_max_ = 3428, 2934, 1590, 1513, 1461, 1418, 1370, 1327, 1271, 1234, 1125, 1032, 1005, 799, and 707 cm^−1^; ^1^H NMR (methanol-*d*_4_, 400 MHz) data, see Table 1; ^13^C NMR (methanol-*d*_4_, 100 MHz) data, see Table 2; HRESIMS *m/z* 411.1774 [M + Na]^+^ (calcd for C_22_H_28_NaO_6_, 411.1784).

#### Acortatarinowin M (**7**)

Light yellow gum, 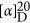 +12 (*c* = 0.1, MeOH); UV (MeOH) *λ*_max_ (log *ε*) = 208 (4.73) and 272 (4.01) nm; ECD (MeOH) *λ*_max_ (Δ*ε*) 210 (+0.02), 221 (−1.41), 241 (−0.93), and 296 (−0.41) nm; IR *ν*_max_ = 3479, 2937, 1717, 1590, 1500, 1461, 1416, 1335, 1228, 1126, 1033, 1000, 831, and 763 cm^−1^; ^1^H NMR (methanol-*d*_4_, 400 MHz) data, see Table 1; ^13^C NMR (methanol-*d*_4_, 100 MHz) data, see Table 2; HRESIMS *m/z* 459.1619 [M + Na]^+^ (calcd for C_22_H_28_NaO_9_, 459.1631).

#### Acortatarinowin N (**8**)

Light yellow gum, 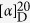 −23 (*c* = 0.1, MeOH); UV (MeOH) *λ*_max_ (log *ε*) = 207 (4.77), 263 (4.22), and 293 (3.93) nm; ECD (MeOH) 215 (Δ*ε −*0.70), 224 (Δ*ε −*0.78), 238 (Δ*ε* +2.13), 296 (−1.68) nm; IR (KBr) *ν*_max_ 3456, 2939, 2714, 1714, 1593, 1508, 1463, 1416, 1328, 1270, 1219, 1126, 1057, 1004, 762 cm^–1^; ^1^H NMR (methanol-*d*_4_, 400 MHz) data, see Table 1; ^13^C NMR (methanol-*d*_4_, 100 MHz) data, see Table 2; (+)-HREIMS *m/z* 429.1464 [M + Na]^+^ (calcd for C_21_H_26_NaO_8_, 429.1525).

#### (S)-MTPA Derivative of **7**

^1^H NMR (methanol-*d*_4_, 400 MHz) *δ*_H_ 7.50−7.15 (5H, overlap, aromatic protons), 6.59 (2H, s, H-2,6), 6.10 (1H, d, *J* = 7.2 Hz, H-7), 4.85 (m, H-8), 1.016 (1H, d, *J* = 6.4 Hz, H-9), 7.35 (2H, s, H-2′,6′), HRESIMS *m/z* 675.2020 [M + Na]^+^ (calcd for C_32_H_35_F_3_NaO_11_, 675.2029).

#### (R)-MTPA Derivative of **7**

^1^H NMR (methanol-*d*_4_, 400 MHz) *δ*_H_ 7.50−7.15 (5H, overlap, aromatic protons), 6.81 (2H, s, H-2,6), 6.21 (1H, d, *J* = 7.2 Hz, H-7), 4.80 (m, H-8), 1.014 (1H, d, *J* = 6.4 Hz, H-9), 7.31 (2H, s, H-2′,6′), HRESIMS *m/z* 675.2019 [M + Na]^+^ (calcd for C_32_H_35_F_3_NaO_11_, 675.2029).

### Single Crystal X-ray Diffraction Analyses and Crystallographic Data of Compounds (±)-1 and 4

The crystallographic data of **(±)−1** CCDC 1434906) and **4** (CCDC 1028833) have been deposited in the Cambridge Crystallographic Data Centre.

#### Crystallographic Data of Compound (±)-1

C_24_H_32_O_8_, *M* = 448.5, monoclinic, *a* = 14.8444(2) Å, *b* = 10.0477(2) Å, *c* = 8.05590(10) Å, *α* = 90.00°, *β* = 91.0230(10)°, *γ* = 90.00°, *V* = 1201.36(3) Å^3^, *T* = 296 (2) K, space group *P-2yc*, *Z* = 2, *μ*(CuKα) = 1.54178 mm^−1^, 9895 reflections measured. The final *R*_*1*_ values were 0.0226 (*I *> 2*σ* (*I*)). The final *wR*(*F*^2^) values were 0.0911 (*I *> 2*σ*(*I*)). The final *R*_*1*_ values were 0.0338 (all data). The final *wR*(*F*^2^) values were 0.0906 (all data). The goodness of fit on *F*^2^ was 1.048. Flack parameter = 0.19 (12).

#### Crystallographic Data of Compound 4

C_23_H_28_O_7_, *M* = 416.45, monoclinic, *a* = 9.8071(4) Å, *b* = 7.9422(3) Å, *c* = 13.6711(5) Å, *α* = 90.00°, *β* = 99.0390(10)°, *γ* = 90.00°, *V* = 1051.62(7) Å^3^, *T* = 100(2) K, space group *P*21, *Z* = 2, *μ*(CuKα) = 0.801 mm^−1^, 8314 reflections measured, 3314 independent reflections (*R*_*int*_ = 0.0370). The final *R*_*1*_ values were 0.0338 (*I *> 2*σ*(*I*)). The final *wR*(*F*^2^) values were 0.0983 (*I* > 2*σ*(*I*)). The final *R*_*1*_ values were 0.0339 (all data). The final *wR*(*F*^2^) values were 0.0983 (all data). The goodness of fit on *F*^2^ was 1.094. Flack parameter = 0.17(15). The Hooft parameter is 0.09(5) for 1354 Bijvoet pairs.

### Preparation of the (*R*) and (*S*)-MTPA Ester of 7^29^

Compound **7** (0.6 mg) was dissolved in 2.0 mL anhydrous CH_2_Cl_2_. Dimethylaminopyridine (30.0 mg), 1-ethyl-3-(3-dimethylaminopropyl)-carbodiimide hydrochloride (20 *μ*L), and (*R*)-MTPA chloride (25.0 *μ*L) were added in sequentially. The reaction mixture was stirred for 3 h at room temperature under N_2_. The solution was evaporated under reduced pressure. The residue was passed to a small silica gel CC eluting with petroleum ether–acetone (5:1) to afford the (*S*)-MTPA ester of **7**. The (*R*)-MTPA ester of **7** was prepared with (*S*)-MTPA chloride in the same manner.

### DPPH Radical Scavenging Activity

The free radical scavenging activity of each isolated new neolignans and lignans was tested using a DPPH assay previously described^30^. Briefly, the tested compounds (6.25, 12.5, 25, 50, 75, and 100*μ*g/mL) were reacted with DPPH (100 *μ*g/mL) in EtOH (with 5% DMSO). The reaction was placed in the wells of 96-well plates at room temperature in the dark. After 30 min of incubation, the optical density of the reaction mixture at 515 nm was read using a microplate reader. All the tests were performed three times, and the results were averaged. Vitamin E was used as the positive control. An EtOH solution (with 5% DMSO) was used as a control.

## Additional Information

**How to cite this article**: Lu, Y. *et al.* Antioxidant Lignans and Neolignans from Acorus tatarinowii. *Sci. Rep.* **6**, 22909; doi: 10.1038/srep22909 (2016).

## Supplementary Material

Supplementary Information

Supplementary checkcif 1

Supplementary checkcif 4

## Figures and Tables

**Table 1:** H NMR Data for Compounds **1**−**8** (400 MHz, *J* in Hz).

No.	1^b^	2^b^	3^a^	4^b^	5^b^	6^a^	7^a^	8^a^
2			6.82 s	6.62 s	7.04 s	6.63 s	6.76 s	6.77 s
3	6.51 s							
4					6.93 s			
5		5.88 s						
6	7.07 s		6.82 s	6.62 s	6.93 s	6.63 s	6.76 s	6.77 s
7	5.02 d (9.3)	6.45 s	4.41 d (9.3)	4.38 d (9.5)	4.41 d (9.0)	4.52 d (7.4)	4.71 d (5.9)	4.73 d (4.9)
8	3.76 m		1.77 m	2.62 m	2.45 m	2.24 m	4.42 m	4.65 m
9	1.09 d (6.3)	1.86 s	1.08 d (6.6)	1.07 d (6.6)	1.03 d (6.8)	1.12 d (7.0)	1.13 d (6.3)	1.31 d (6.1)
2′			6.956 d (2.0)	7.56 d (2.0)	7.58 d (2.0)	6.78 d (1.8)	7.33 s	7.53 d (2.0)
3′	6.50 s	6.53 s						
5′			6.97 d (8.2)	6.92 d (8.8)	7.08 d (8.5)	6.72 d (8.0)		6.97d (8.5)
6′	7.08 s	6.45 s	6.97 dd (8.2, 2.0)	7.55 dd (8.8, 2.0)	7.71 dd (8.5, 2.0)	6.64 dd (8.0, 1.8)	7.33 s	7.57 dd (8.5, 2.0)
7′	5.25 d (3.2)	4.44 s	5.15 d (8.8)			2.83 dd (13.4, 5.0), 2.45 dd (13.4, 11.1)		
8′	4.34 m	2.31 q (7.3)	2.28 m	3.83 m	4.03 m	2.61 m		
9′a	1.12 d (6.8)	1.11 d (7.2)	0.66 d (7.0)	4.30 dd (9.3, 8.8)	4.28 dd (9.0, 8.5)	4.02 dd (8.4, 6.3)		
9′b				4.15 dd (9.3, 7.5)	4.11 dd (9.0, 6.8)	3.73 dd (8.4, 5.1)		
OMe-2	3.82 s							
OMe-3			3.87 s	3.87 s	3.86 s	3.83 s	3.83 s	3.82 s
OMe-4	3.89 s	3.79 s	3.78 s	3.83 s		3.75 s	3.74 s	3.70 s
OMe-5	3.88 s		3.87 s	3.87 s	3.86 s	3.83 s	3.83 s	3.82 s
OMe-2′	3.83 s	3.90 s						
OMe-3′			3.83 s	3.94 s	3.90		3.89 s	3.85 s
OMe-4′	3.89 s	3.84 s	3.83 s	3.95 s	3.93	3.83 s		
OMe-5′	3.90 s	3.64 s					3.89 s	
OMe-7′							3.90 s	3.86 s

^a^in methanol-*d*_4_.

^b^in CDCl_3_.

**Table 2:** ^13^C NMR Data for Compounds **1**−**8** (100 MHz).

No.	1^b^	2^b^	3^a^	4^b^	5^b^	6^a^	7^a^	8^a^
1	119.8	137.3	138.7	136.0	134.3	140.0	138.4	138.7
2	151.8	132.5	104.9	103.7	111.4	104.2	105.5	105.6
3	97.7	181.5	154.6	153.4	150.7	154.5	154.2	154.1
4	149.4	159.6	138.1	137.8	112.7	138.4	138.4	138.3
5	143.3	106.2	154.6	153.4	150.7	154.5	154.2	154.1
6	111.7	186.6	104.9	103.7	120.8	104.2	105.5	105.6
7	67.9	113.3	88.9	89.2	90.2	88.6	78.5	77.1
8	78.8	153.3	49.8	45.9	47.7	46.6	84.9	80.0
9	17.1	23.4	15.1	15.4	15.1	12.8	17.1	15.6
1′	119.5	121.4	135.1	130.3	131.4	133.5	126.4	123.9
2′	150.0	151.1	112.4	110.2	111.8	113.5	107.9	114.0
3′	97.0	99.1	149.9	149.4	150.5	145.8	154.4	150.1
4′	148.8	148.9	150.2	153.9	155.6	149.0	142.2	153.1
5′	143.6	143.0	112.7	110.5	111.9	116.2	154.4	115.4
6′	111.2	112.2	120.9	123.2	124.9	122.2	107.9	124.6
7′	75.1	35.0	84.6	197.6	200.2	34.3	168.1	168.4
8′	70.8	41.5	47.1	54.4	55.1	45.4		
9′	12.3	19.5	15.3	70.8	71.5	73.4		
OMe-2	56.8							
OMe-3			56.7	56.3	56.5	56.6	56.7	56.5
OMe-4	56.3	56.3	61.1	60.9		61.1	61.1	61.1
OMe-5	56.3		56.7	56.3	56.5	56.6	56.7	56.5
OMe-2′	56.8	57.2						
OMe-3′			56.5	56.1	56.5		56.6	56.5
OMe-4′	56.3	56.3	56.5	56.2	56.6	56.4		
OMe-5′	56.7	57.2					56.6	
OMe-7′							52.8	52.5

^a^in methanol-*d*_4_.

^b^in CDCl_3_.

**Table 3:** DPPH Radical Scavenging Activity of New Compounds *in vitro*.

Compounds	Concentrations (*μ*g/mL)	Scavenging ratio (%)	IC_50_ (*μ*g/mL)
1a	100	−2.292 ± 0.263	
1b	100	−2.893 ± 0.278	
2a	100	−2.477 ± 0.351	
2b	100	−4.892 ± 0.088	
3a	100	−1.487 ± 0.351	
3b	100	−1.363 ± 0	
4	100	−1.301 ± 0.263	
5	100	−1.796 ± 0.088	
6	100	84.784 ± 0.756	16.437 ± 0.22
	50	71.794 ± 0.611	
	25	59.629 ± 0.328	
	12.5	43.712 ± 0.435	
	6.25	29.609 ± 0.993	
7	100	−1.796 ± 0.088	
8	100	−2.145 ± 0.078	
Trolox	50	95.629 ± 0.372	3.895 ± 0.38
	25	94.805 ± 0.611	
	12.5	94.433 ± 0.682	
	6.25	69.238 ± 3.335	
	3.125	41.114 ± 4.279	

The data are expressed as the means ± SEM. Three independent experiments were performed. Trolox (vitamin E) was used as the positive control.
