# Supplementary material for: Antioxidant Lignans and Neolignans from Acorus tatarinowii
Source: Sci Rep. 2016 Mar 10;6:22909. doi: 10.1038/srep22909 (PMC4785495; doi:10.1038/srep22909)
Supplement: Supplementary Information [file srep22909-s1.pdf]

Antioxidant Lignans and Neolignans from Acorus tatarinowii

Yuanyuan Lu,[1,2] Yongbo Xue,[1] Shenjie Chen,[1] Hucheng Zhu,[1] Jinwen Zhang,[2] Xiao-Nian Li,[3] Jianping Wang,[1] JunJun Liu,[1] Changxing Qi,[1] Guang Du[2,]* & Yonghui Zhang[1,]*

[1]Hubei Key Laboratory of Natural Medicinal Chemistry and Resource Evaluation, School of Pharmacy, Tongji Medical College, Huazhong University of Science and Technology, Wuhan 430030, Hubei Province, People's Republic of China

[2]Tongji Hospital Affiliated to Tongji Medical College, Huazhong University of Science and Technology, Wuhan 430030, Hubei Province, People's Republic of China

[3]State Key Laboratory of Phytochemistry and Plant Resources in West China, Kunming Institute of Botany, Chinese Academy of Sciences, Kunming 650204, People's Republic of China

* **Corresponding Authors**

* Tel./fax: +86-027-83692892

Emails: zhangyh@mails.tjmu.edu.cn (Y.Z.);

tjyxb@sina.com (G.D.)

Figure S1. (+)-HR-ESI-MS spectrum of **1** (**1a/1b**)......1

Figure S2. IR spectrum of **1** (**1a/1b**)......1

Figure S3. UV spectrum of **1** (**1a/1b**)......2

Figure S4. ^{1}H NMR spectrum of **1** (**1a/1b**) in $CDCl_3$......2

Figure S5. ^{13}C NMR spectrum of **1**(**1a/1b**) in $CDCl_3$......3

Figure S6. HSQC spectrum of **1** (**1a/1b**) in $CDCl_3$......3

Figure S7. HMBC spectrum of **1** (**1a/1b**) in $CDCl_3$......4

Figure S8. ^{1}H-^{1}H COSY spectrum of **1** (**1a/1b**) in $CDCl_3$......4

Figure S9. NOESY spectrum of **1** (**1a/1b**) in $CDCl_3$......5

Figure S10. (+)-HR-ESI-MS spectrum of **2** (**2a/2b**)......5

Figure S11. IR spectrum of **2** (**2a/2b**)......6

Figure S12. UV spectrum of **2** (**2a/2b**)......6

Figure S13. ^{1}H NMR spectrum of **2** (**2a/2b**) in $CDCl_3$......7

Figure S14.^{13}C NMR spectrum of **2** (**2a/2b**) in $CDCl_3$......7

Figure S15. HSQC spectrum of **2** (**2a/2b**) in $CDCl_3$......8

Figure S16. HMBC spectrum of **2** (**2a/2b**) in $CDCl_3$......8

Figure S17. ^{1}H-^{1}H COSY spectrum of **2** (**2a/2b**) in $CDCl_3$......9

Figure S18. NOESY spectrum of **2** (**2a/2b**) in $CDCl_3$......9

Figure S19. (+)-HR-ESI-MS spectrum of **3** (**3a/3b**)......10

Figure S20. IR spectrum of **3** (**3a/3b**)......10

Figure S21. UV spectrum of **3** (**3a/3b**)......11

Figure S22. ^{1}H NMR spectrum of **3** (**3a/3b**) in CD_3OD......11

Figure S23.^{13}C NMR spectrum of **3** (**3a/3b**) in CD_3OD......12

Figure S24. HSQC spectrum of **3** (**3a/3b**) in CD_3OD......112

Figure S25. HMBC spectrum of **3** (**3a/3b**) in CD_3OD......13

Figure S26. ^{1}H-^{1}H COSY spectrum of **3** (**3a/3b**) in CD_3OD......13

Figure S27. NOESY spectrum of **3** (**3a/3b**) in CD_3OD......14

Figure S28. (+)-HR-ESI-MS spectrum of **4**......14

Figure S29. IR spectrum of **4**......15

Figure S30. UV spectrum of **4**......15

Figure S31. ^{1}H NMR spectrum of **4** in $CDCl_3$........16
Figure S32. ^{13}C NMR spectrum of **4** in $CDCl_3$........16
Figure S33. HSQC spectrum of **4** in $CDCl_3$........17
Figure S34. HMBC spectrum of **4** in $CDCl_3$........17
Figure S35. ^{1}H-^{1}H COSY spectrum of **4** in $CDCl_3$........18
Figure S37. ECD spectrum of **4** in MeOH........18
Figure S38. (+)-HR-ESI-MS spectrum of **5**........19
Figure S39. IR spectrum of **5**........19
Figure S40. UV spectrum of **5**........20
Figure S41. ^{1}H NMR spectrum of **5** in CD_3OD........21
Figure S42. ^{13}C NMR spectrum of **5** in CD_3OD........21
Figure S43. HSQC spectrum of **5** in CD_3OD........22
Figure S44. HMBC spectrum of **5** in CD_3OD........22
Figure S45. ^{1}H-^{1}H COSY spectrum of **5** in CD_3OD........22
Figure S46. NOSEY spectrum of **5** in CD_3OD........23
Figure S47. ECD spectrum of **5** in MeOH........23
Figure S48. (+)-HR-ESI-MS spectrum of **6**........24
Figure S49. IR spectrum of **6** (**6a/6b**)........24
Figure S50. UV spectrum of **6**........25
Figure S51. ^{1}H NMR spectrum of **6** in CD_3OD........25
Figure S52. ^{13}C NMR spectrum of **6** in CD_3OD........26
Figure S53. HSQC spectrum of **6** in CD_3OD........26
Figure S54. HMBC spectrum of **6** in CD_3OD........27
Figure S55. ^{1}H-^{1}H COSY spectrum of **6** in CD_3OD........27
Figure S56. NOESY spectrum of **6** in CD_3OD........28
Figure S57. ECD spectrum of **6** in MeOH........28
Figure S58. (+)-HR-ESI-MS spectrum of **7**........29
Figure S59. IR spectrum of **7**........29
Figure S60. UV spectrum of **7**........30
Figure S61. ^{1}H NMR spectrum of **7** in CD_3OD........31

Figure S62. ^{13}C NMR spectrum of **7** in CD_3OD..31
Figure S63. HSQC spectrum of **7** in CD_3OD...32
Figure S64. HMBC spectrum of **7** in CD_3OD 32
Figure S65. ^{1}H-^{1}H COSY spectrum of **7** in CD_3OD..32
Figure S66. NOESY spectrum of **7** in CD_3OD..33
Figure S67. ECD spectrum of **7** in MeOH...33
Figure S68. (+)-HR-ESI-MS spectrum of **8**...34
Figure S69. IR spectrum of **8**..34
Figure S70. UV spectrum of **8** 35
Figure S71. ^{1}H NMR spectrum of **8** in CD_3OD...35
Figure S72. ^{13}C NMR spectrum of **8** in CD_3OD..36
Figure S73. HSQC spectrum of **8** in CD_3OD...36
Figure S74. HMBC spectrum of **8** in CD_3OD 37
Figure S75. ^{1}H-^{1}H COSY spectrum of **8** in CD_3OD..37
Figure S76. NOESY spectrum of **8** in CD_3OD..38
Figure S77. ECD spectrum of **8** in MeOH...38
Figure S78. (+)-HR-ESI-MS spectrum of *S*-MTPA-**7** ester 39
Figure S79. (+)-HR-ESI-MS spectrum of *R*-MTPA-**7** ester.......................................39
Figure S80. ^{1}H NMR spectrum of *R*/*S*-MTPA-**7** ester in CD_3OD..............................40
Figure S81. ECD spectra calculation..40
Figure S82. Five lowest energy conformers of the isomer 7*S*,7′*R*,8*S*,8′*S* and five lowest energy conformers of the 7*R*,7′*S*,8*R*,8′*R* isomer of compound **1**......................41
Table S1. Relative free energies (ΔG) and equilibrium populations (P) of the conformers of the (7*S*,7′*R*,8*S*,8′*S*)-**1** and (7*R*,7′*S*,8*R*,8′*R*)-**1** 42

Figure S1. (+)-HR-ESI-MS spectrum of **1** (**1a/1b**)

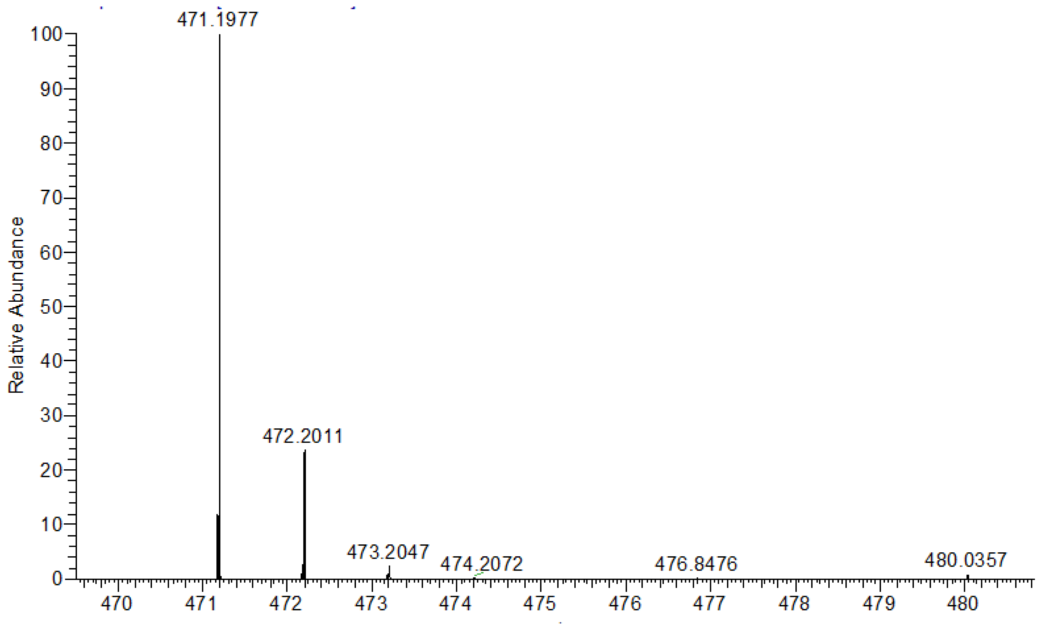

Figure S2. IR spectrum of **1** (**1a/1b**)

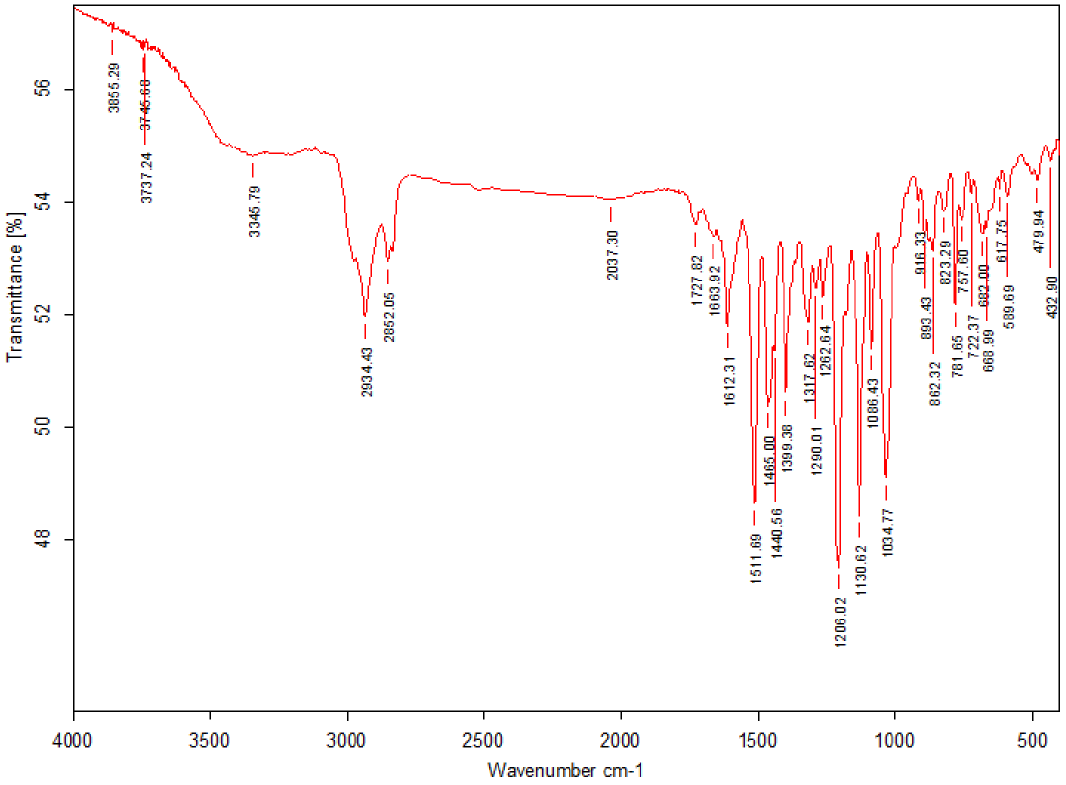

Figure S3. UV spectrum of **1** (**1a/1b**)

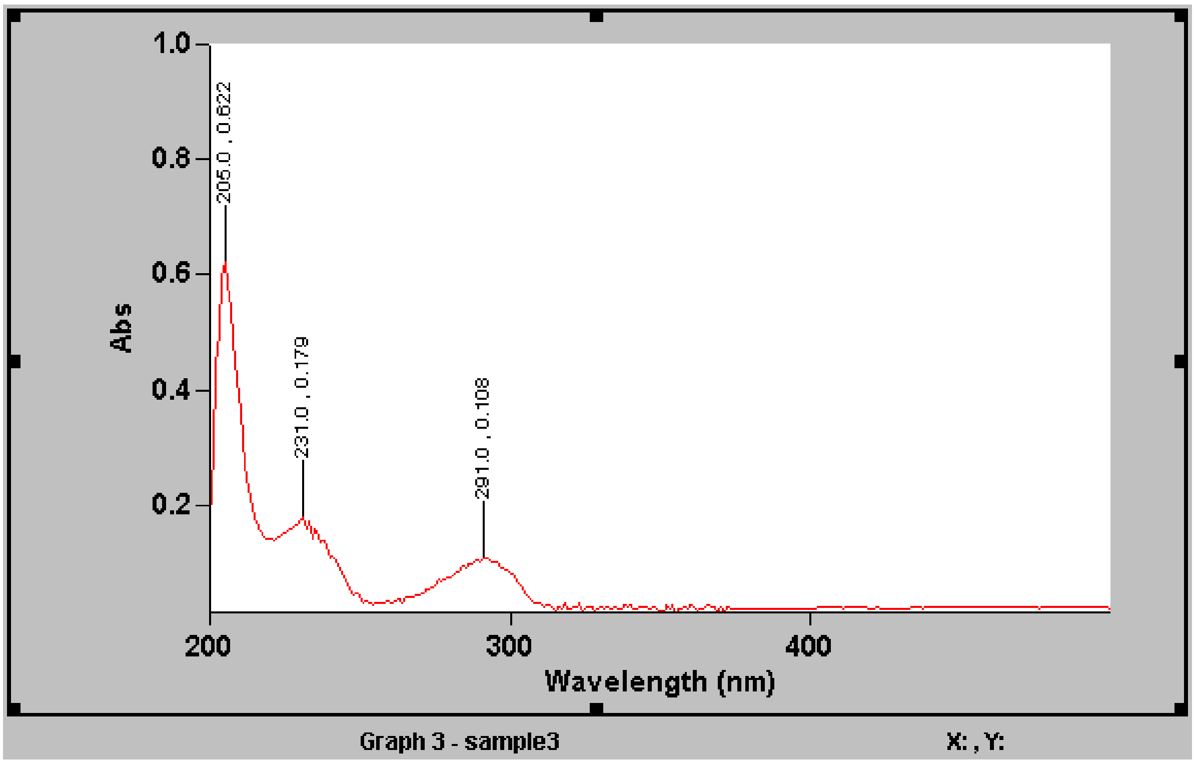

Figure S4. ^{1}H NMR spectrum of **1** (**1a/1b**) in $CDCl_3$

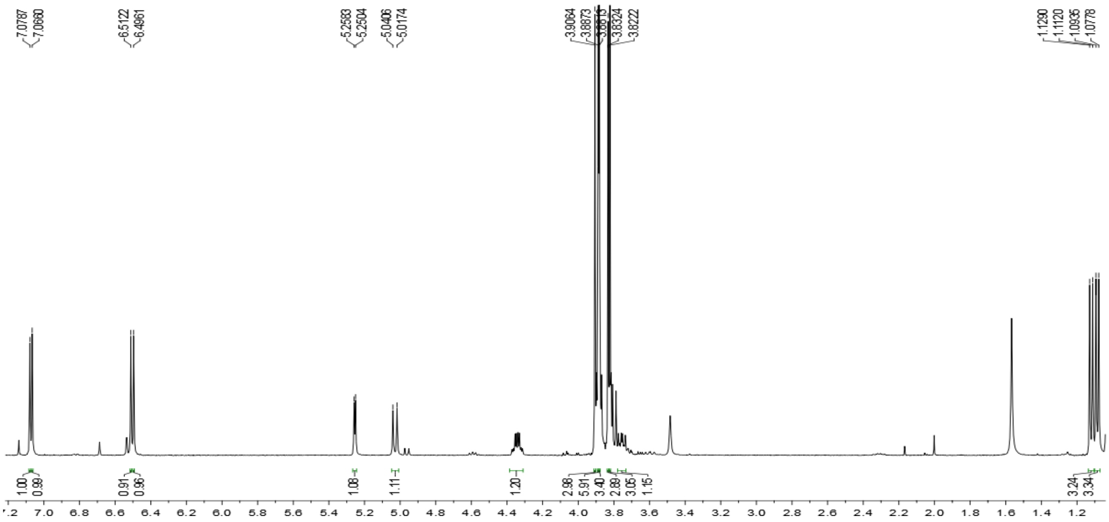

Figure S5. ^{13}C NMR spectrum of **1**(**1a/1b**) in $CDCl_3$

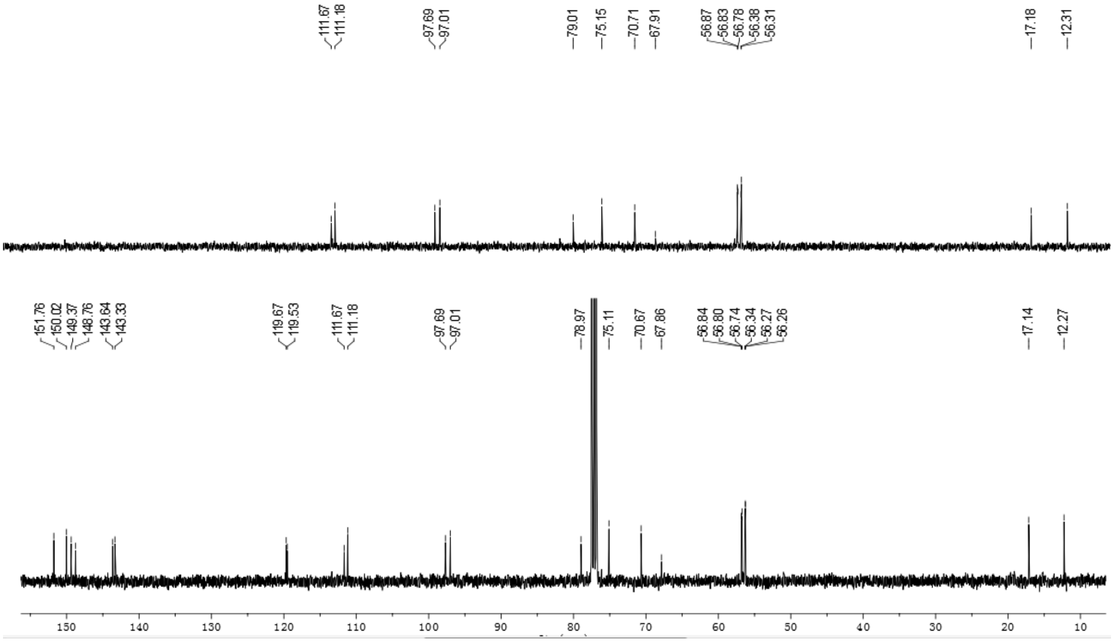

Figure S6. HSQC spectrum of **1** (**1a/1b**) in $CDCl_3$

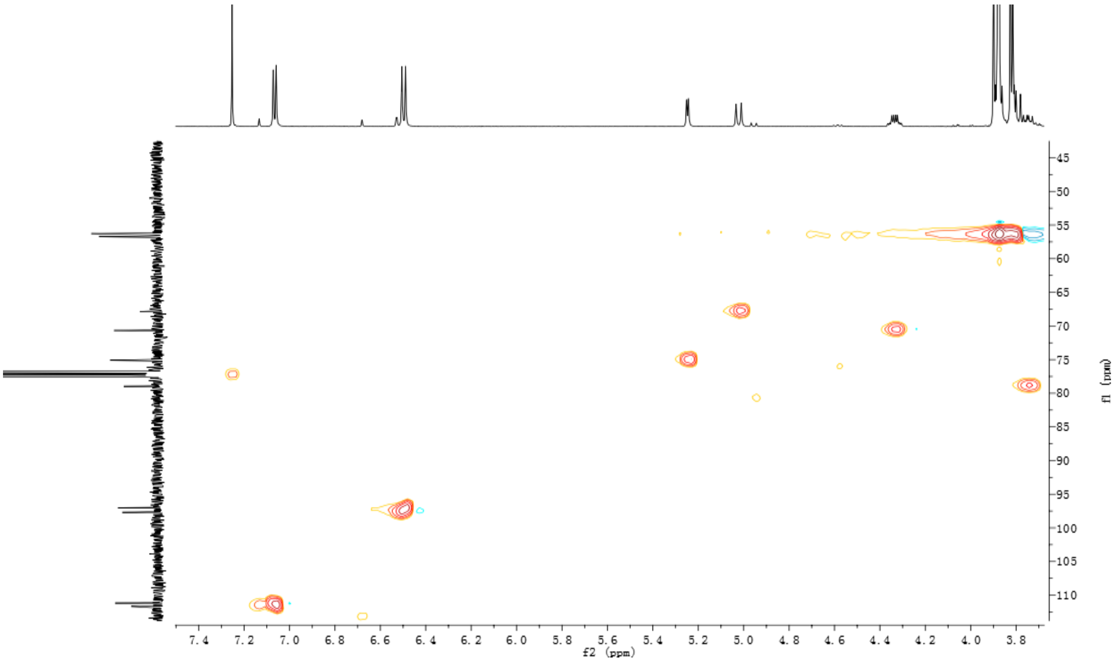

Figure S7. HMBC spectrum of **1** (**1a/1b**) in $CDCl_3$

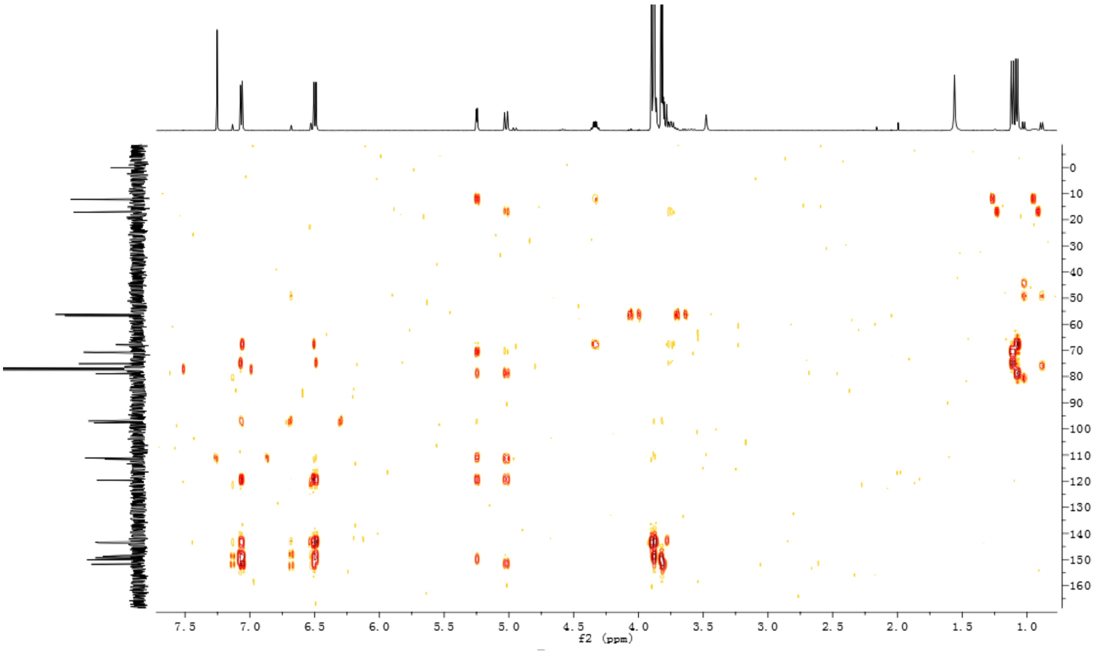

Figure S8. ^1H-^1H COSY spectrum of **1** (**1a/1b**) in $CDCl_3$

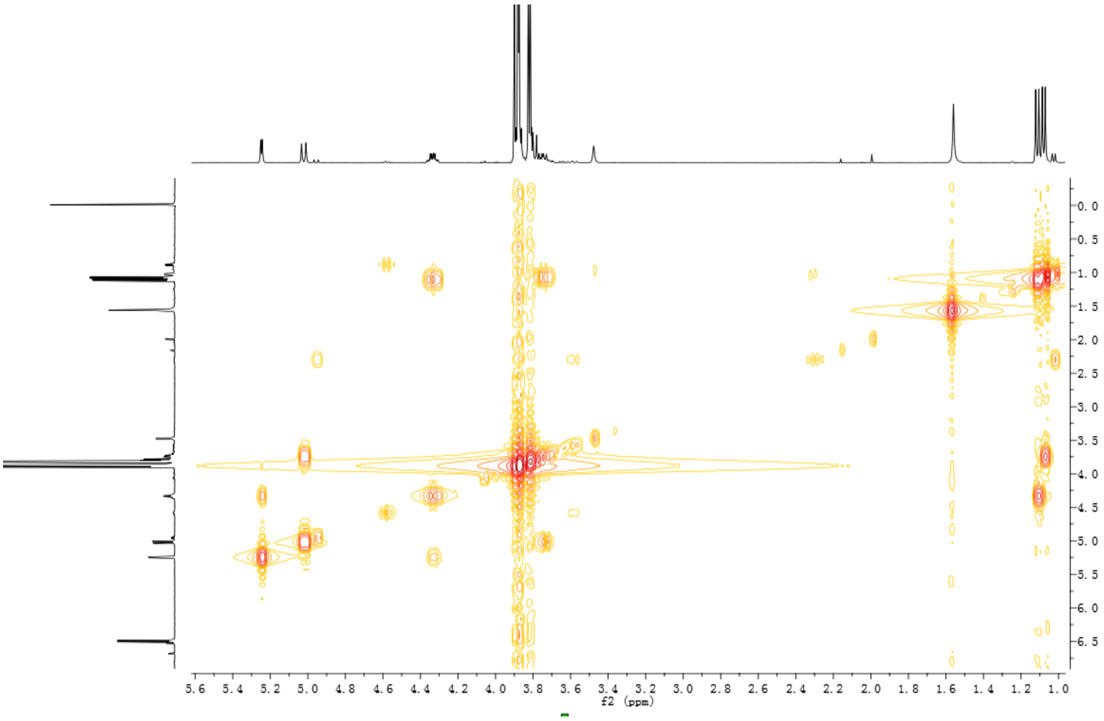

Figure S9. NOESY spectrum of **1** (**1a**/**1b**) in $CDCl_3$

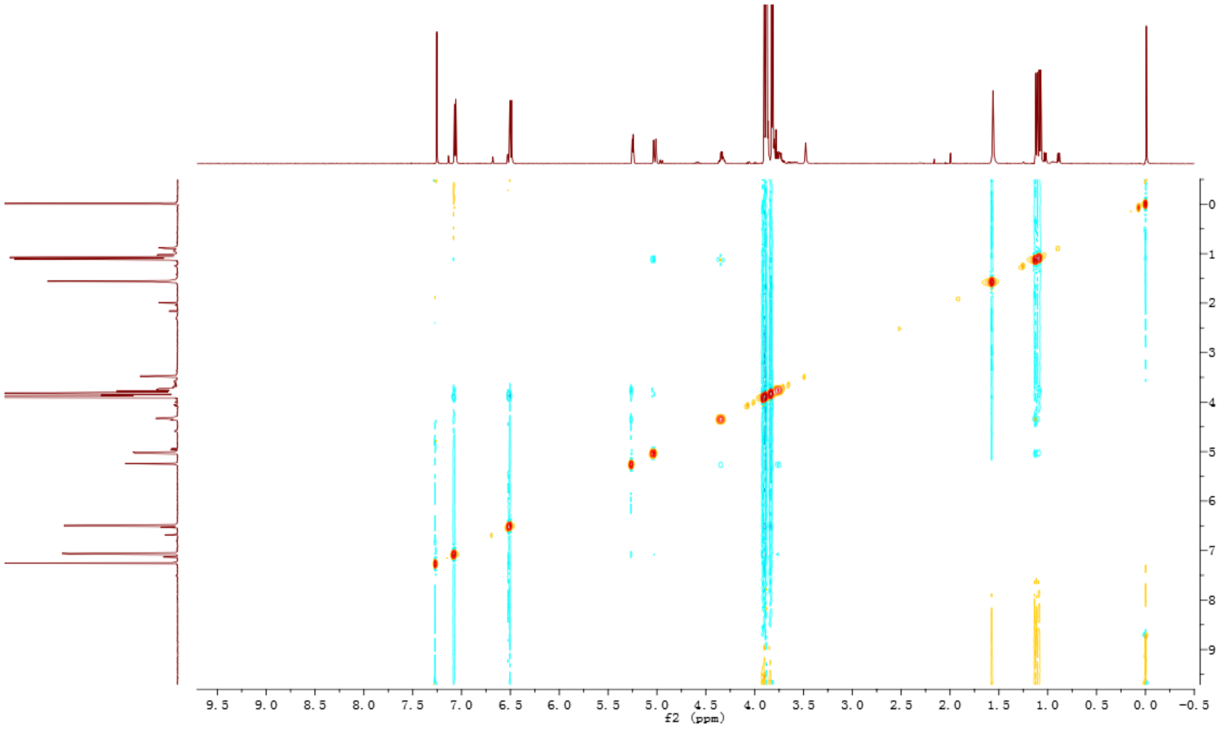

Figure S10. (+)-HR-ESI-MS spectrum of **2** (**2a**/**2b**)

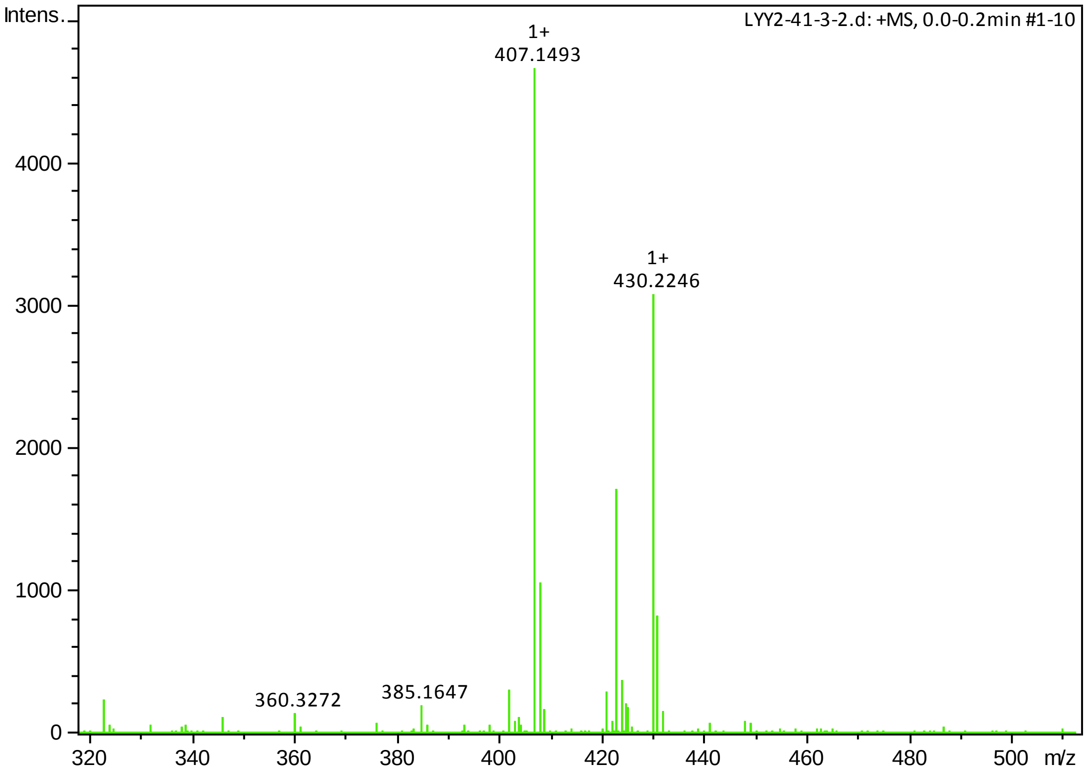

Figure S11. IR spectrum of **2** (**2a/2b**)

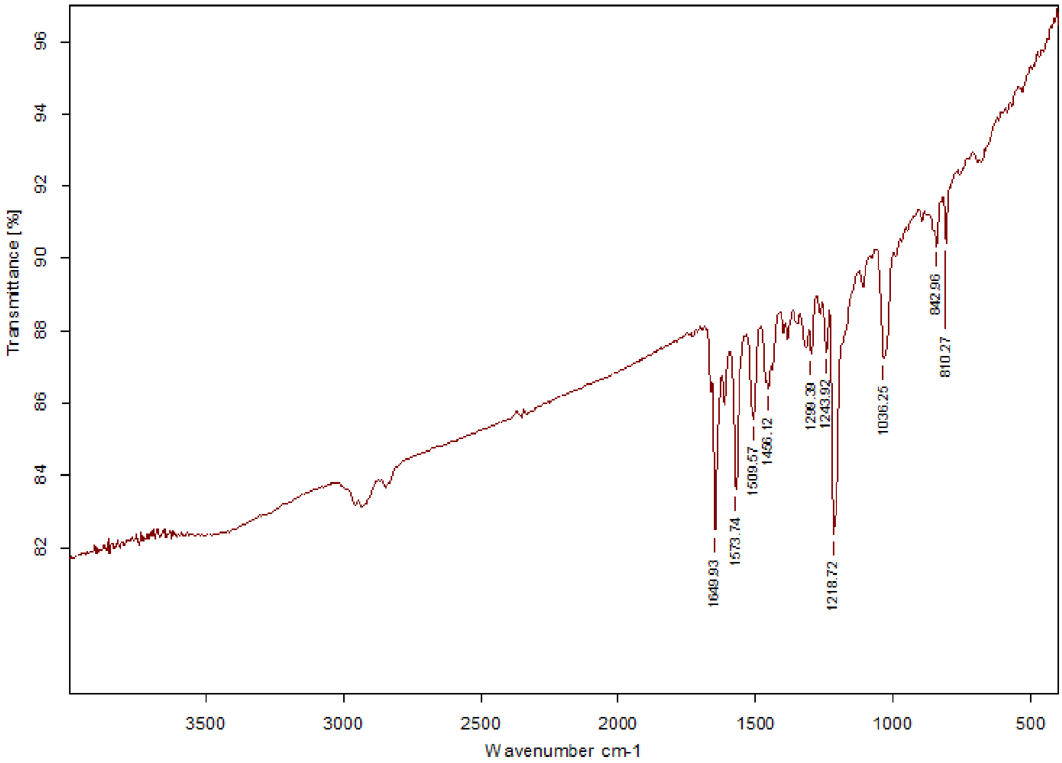

Figure S12. UV spectrum of **2** (**2a/2b**)

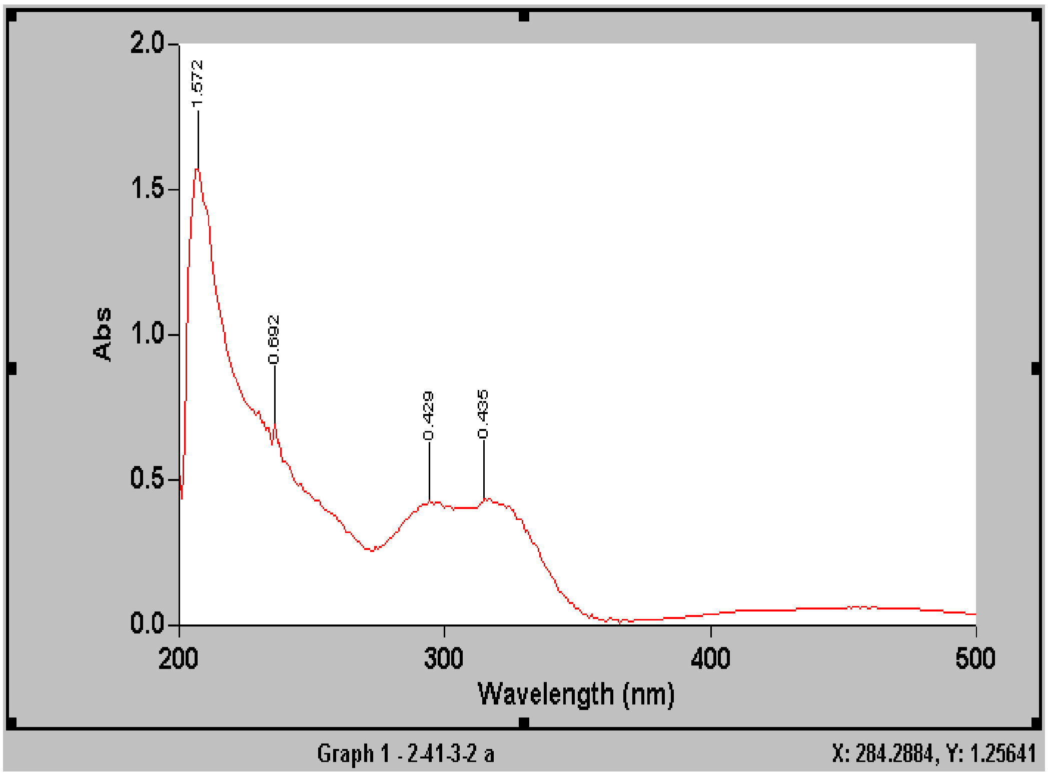

Figure S13. ^{1}H NMR spectrum of **2** (**2a/2b**) in $CDCl_3$

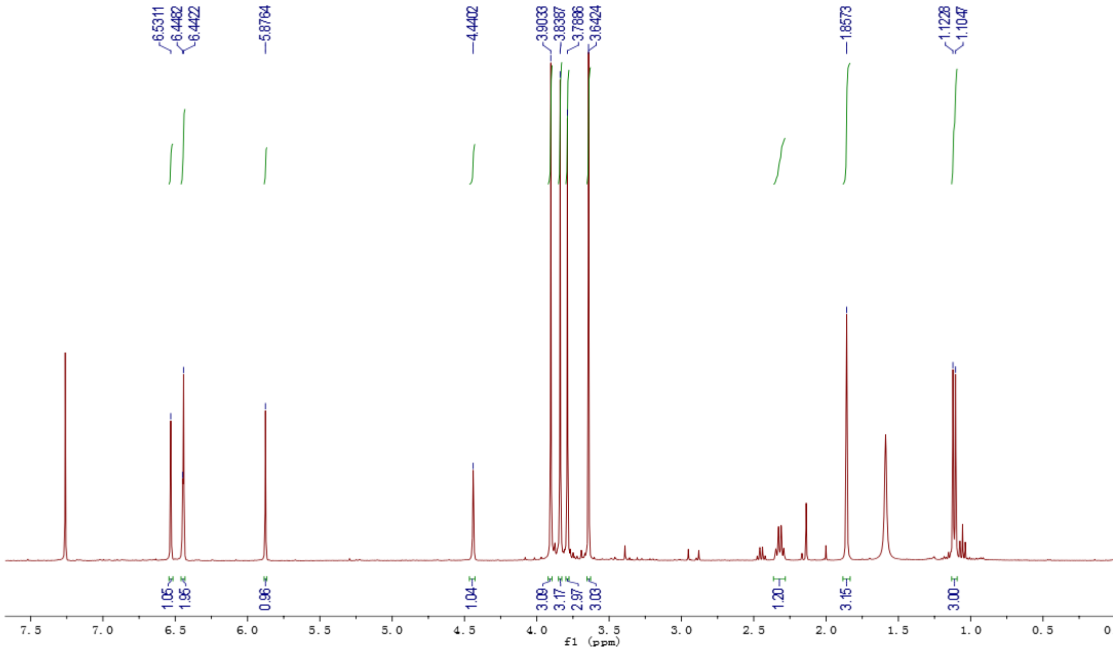

Figure S14.^{13}C NMR spectrum of **2** (**2a/2b**) in $CDCl_3$

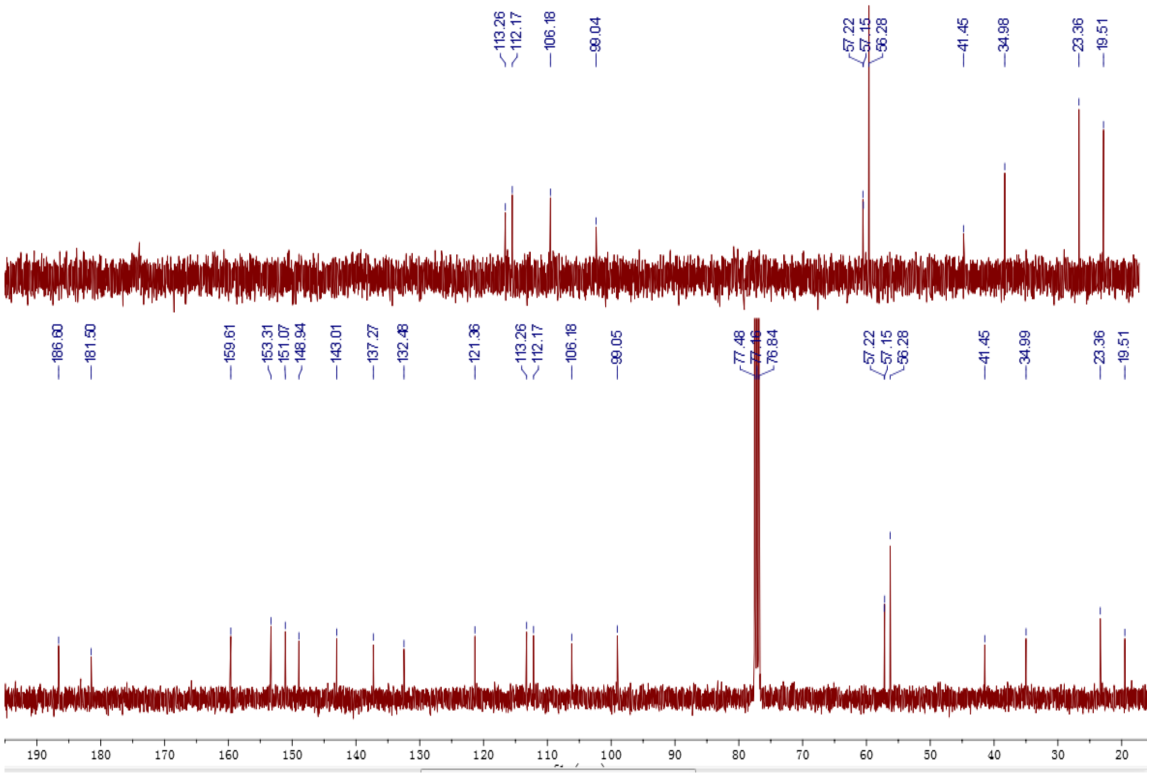

Figure S15. HSQC spectrum of **2** (**2a/2b**) in $CDCl_3$

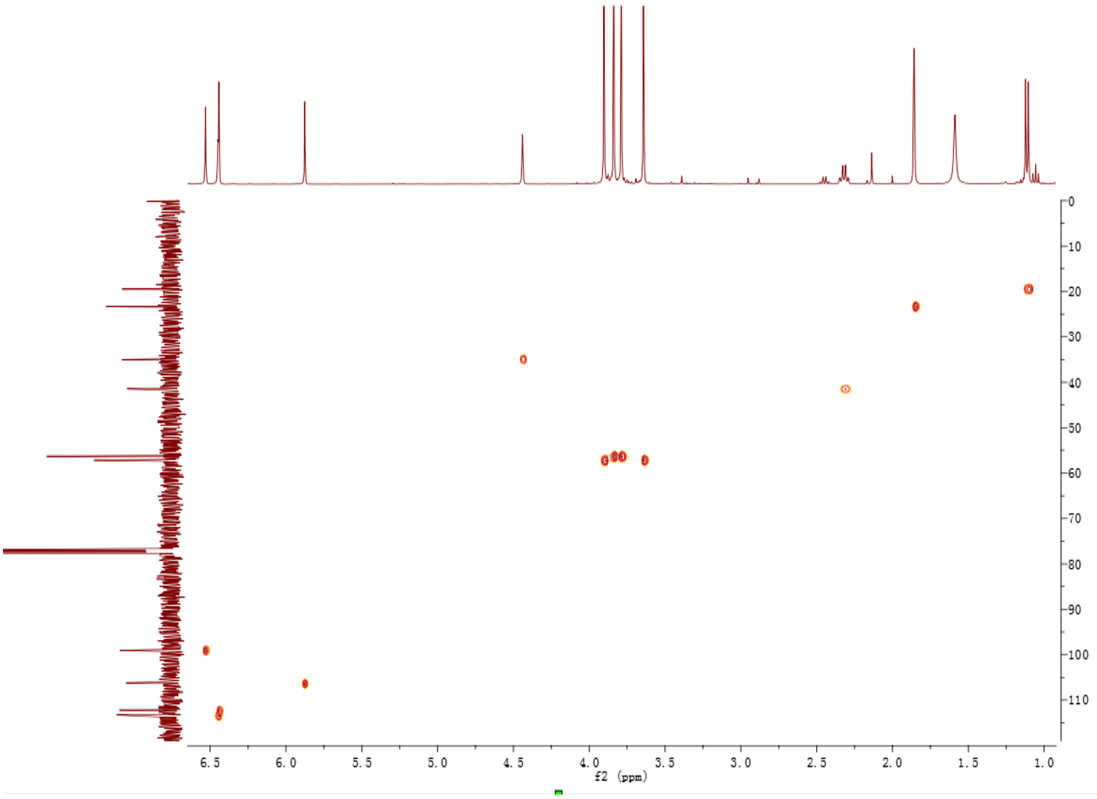

Figure S16. HMBC spectrum of **2** (**2a/2b**) in $CDCl_3$

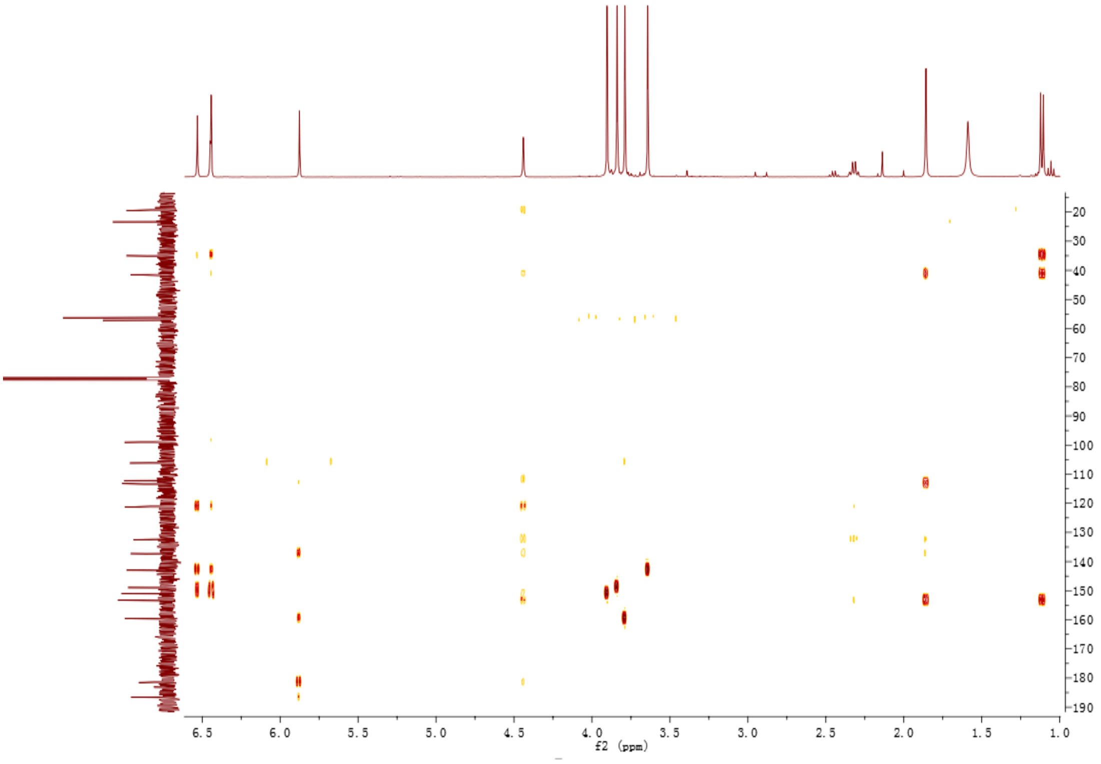

Figure S17. ^{1}H-^{1}H COSY spectrum of **2** (**2a/2b**) in $CDCl_3$

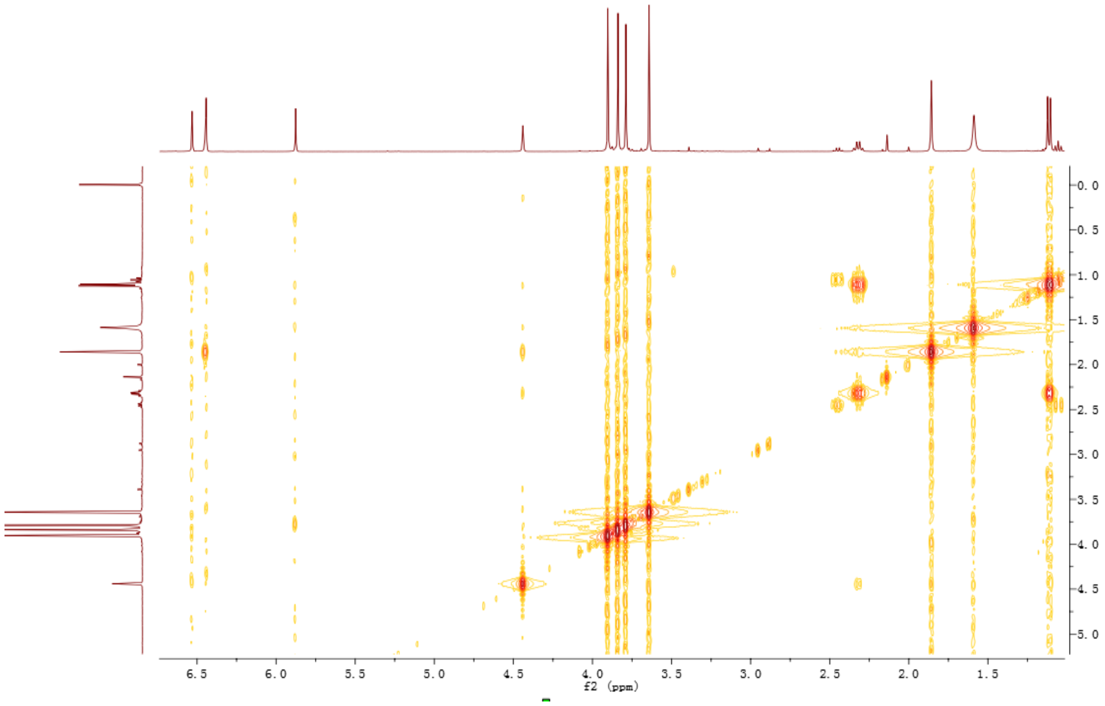

Figure S18. NOESY spectrum of **2** (**2a/2b**) in $CDCl_3$

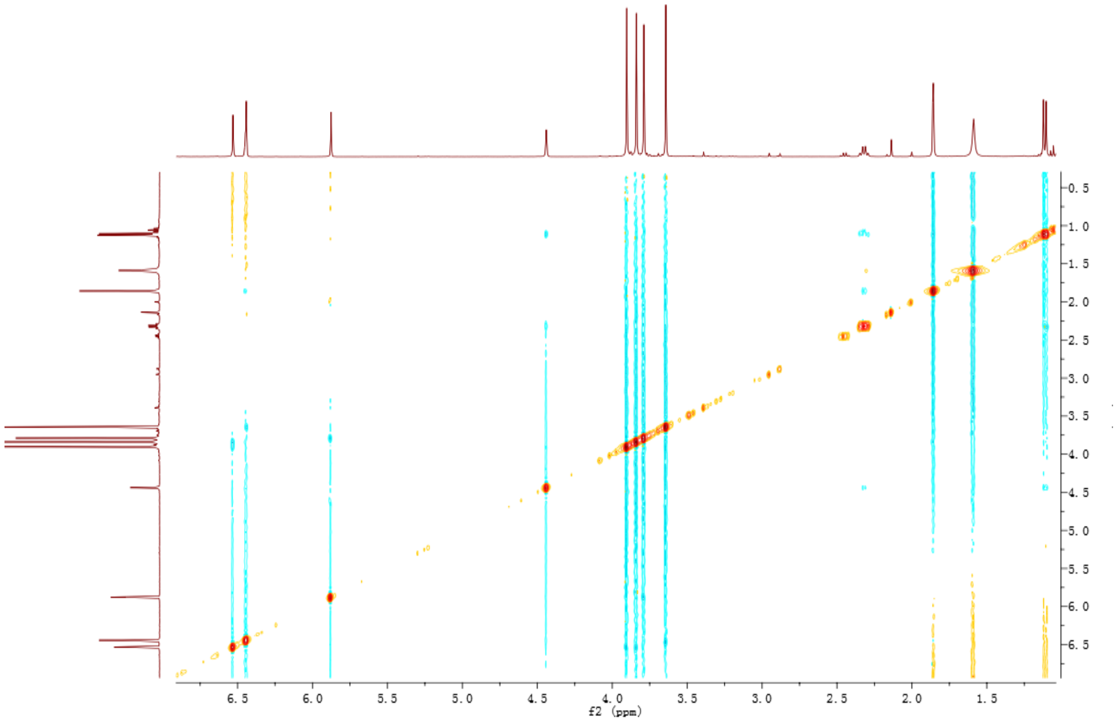

Figure S19. (+)-HR-ESI-MS spectrum of **3** (**3a**/**3b**)

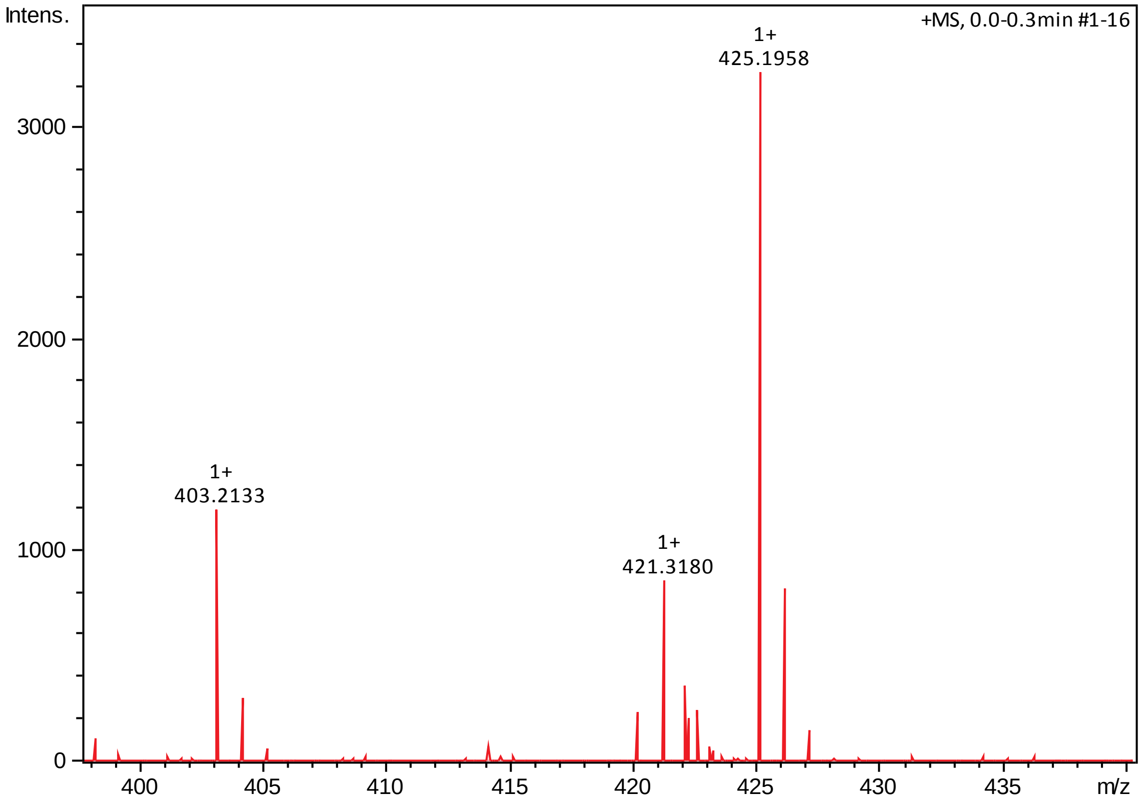

Figure S20. IR spectrum of **3** (**3a**/**3b**)

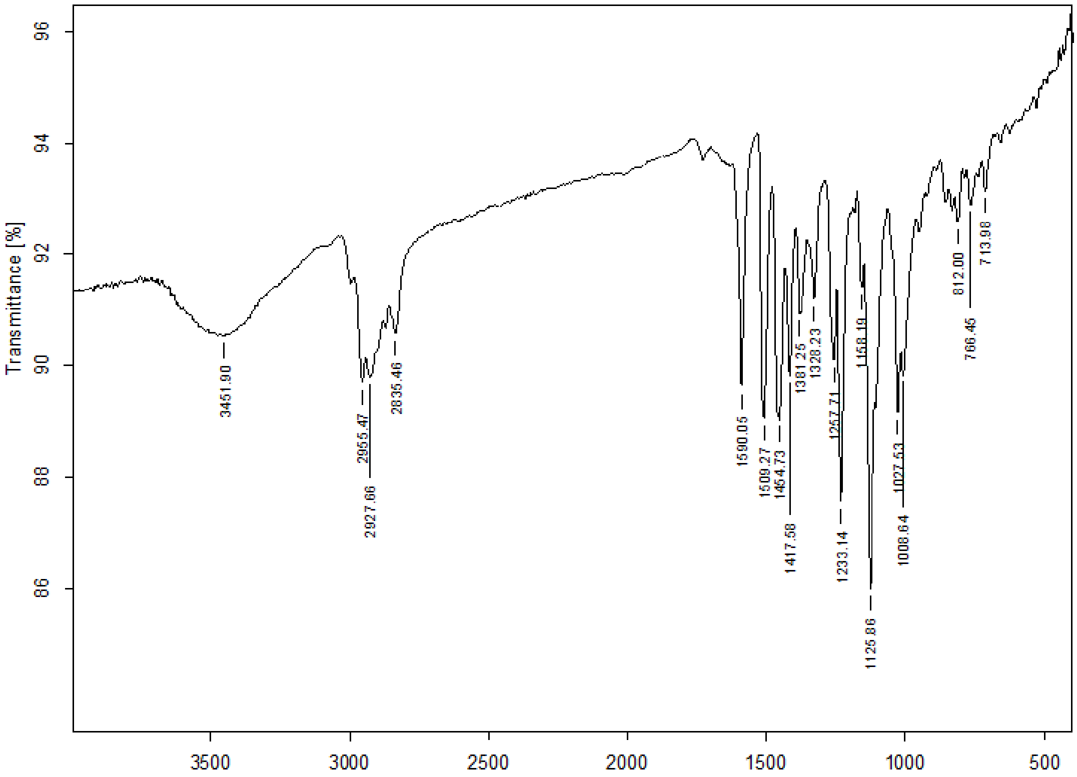

Figure S21. UV spectrum of **3** (**3a/3b**)

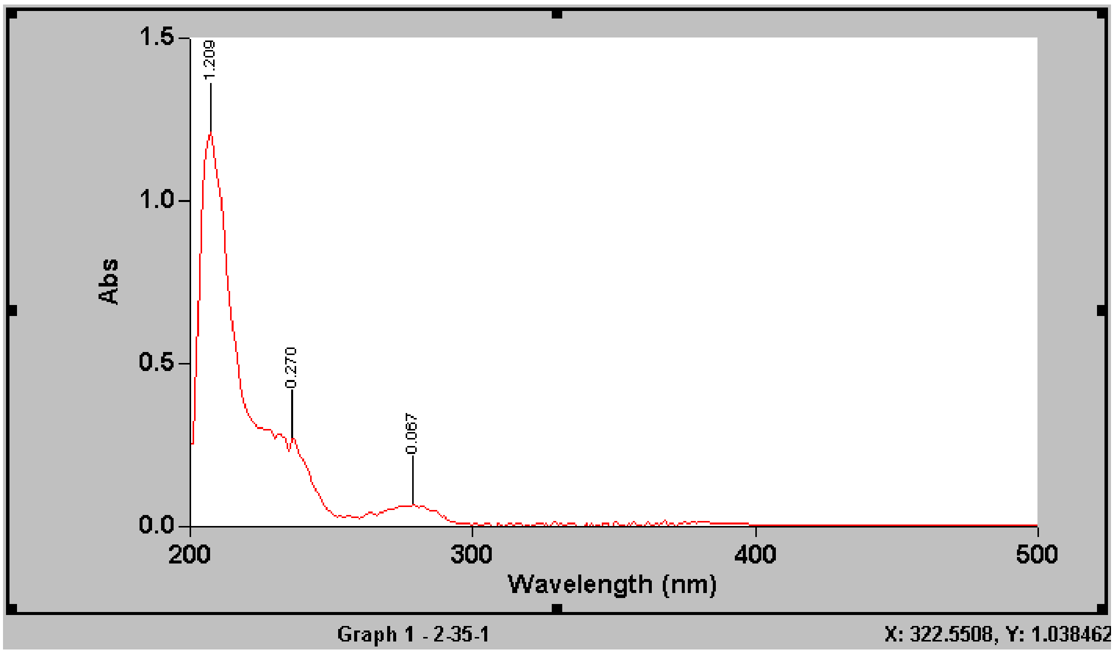

Figure S22. ^{1}H NMR spectrum of **3** (**3a/3b**) in CD_3OD

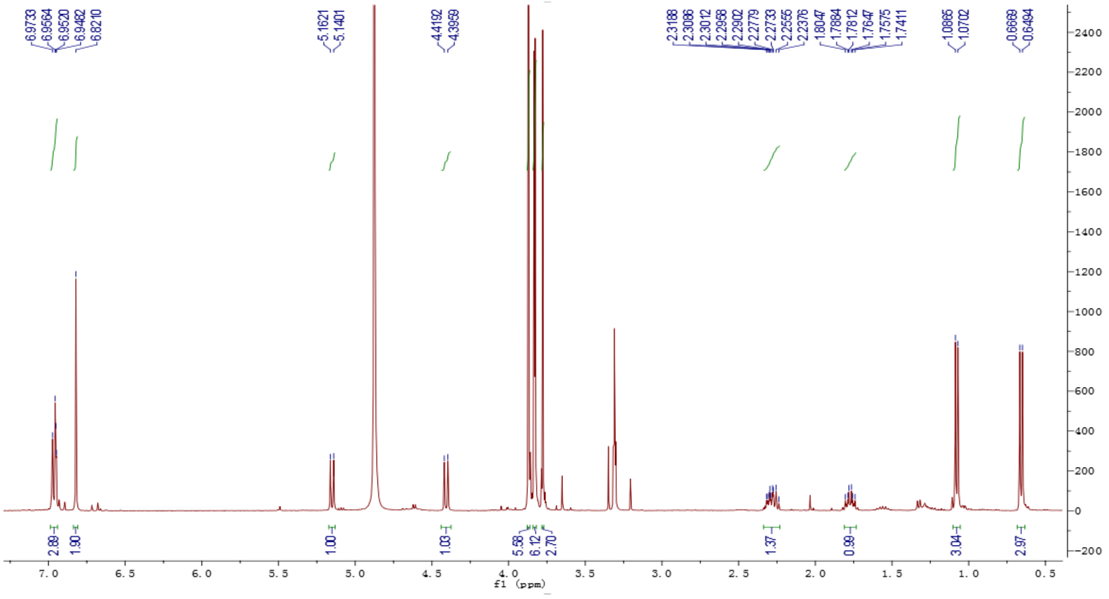

Figure S23.^{13}C NMR spectrum of **3** (**3a/3b**) in CD_3OD

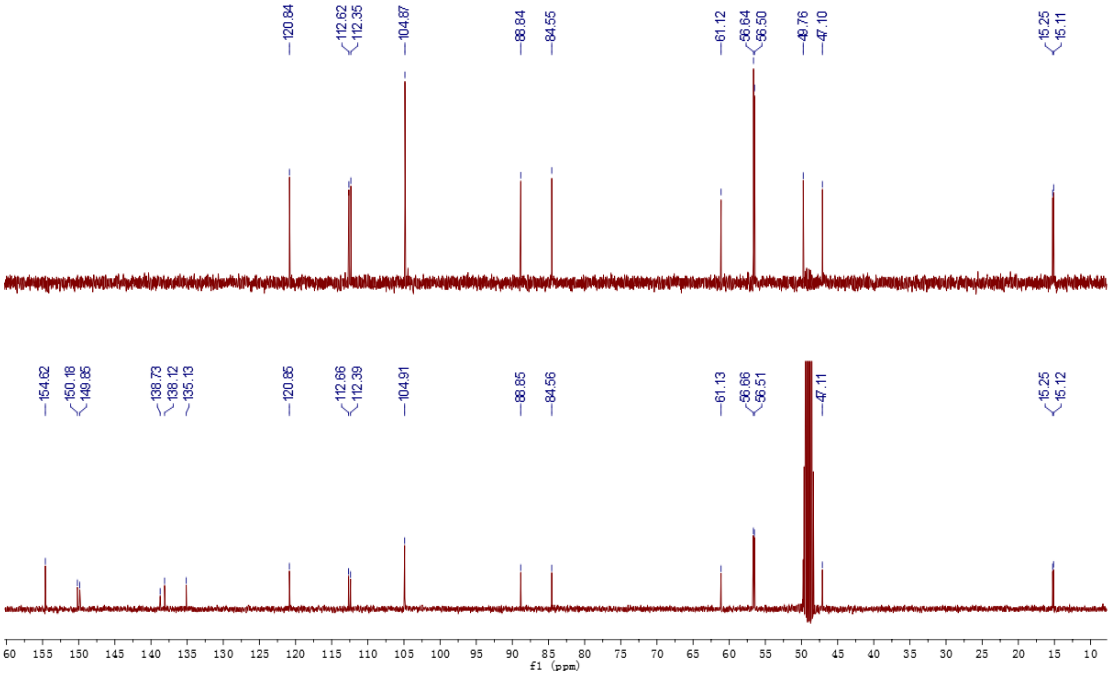

Figure S24. HSQC spectrum of **3** (**3a/3b**) in CD_3OD

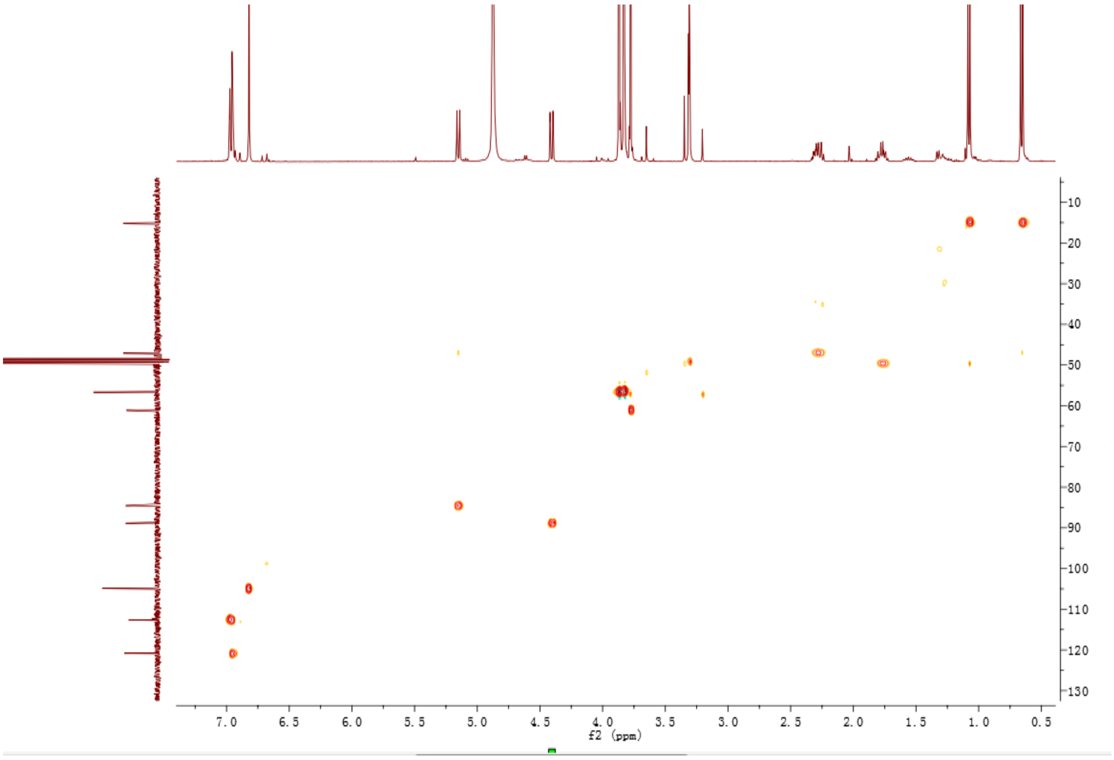

Figure S25. HMBC spectrum of **3** (**3a**/**3b**) in CD_3OD

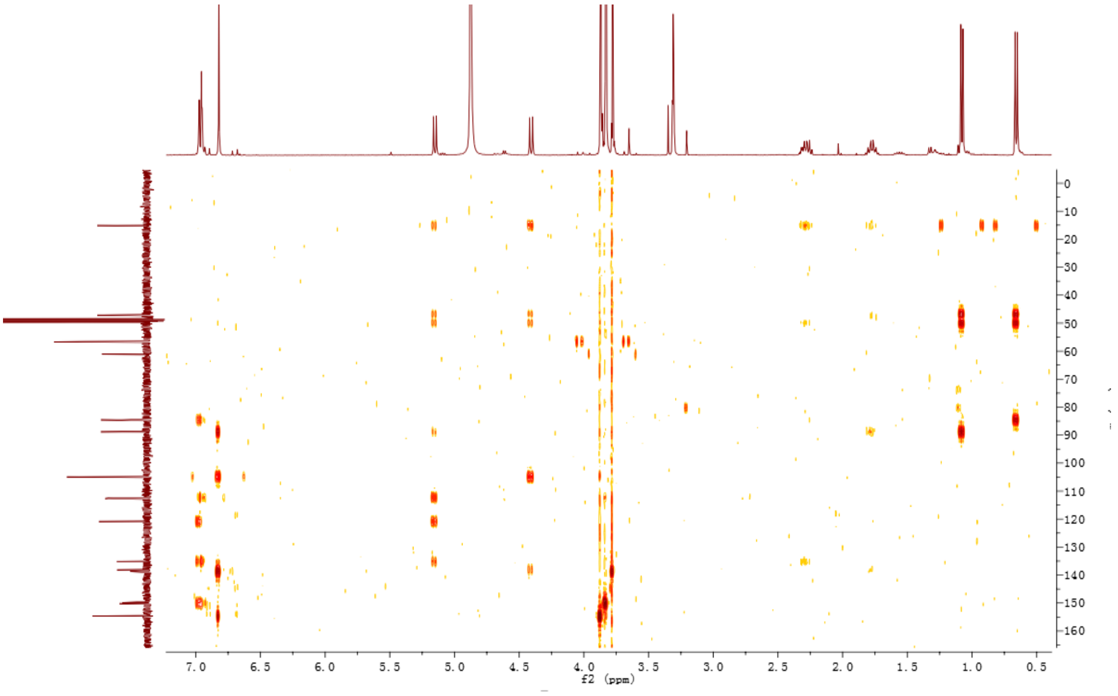

Figure S26. ^{1}H-^{1}H COSY spectrum of **3** (**3a**/**3b**) in CD_3OD

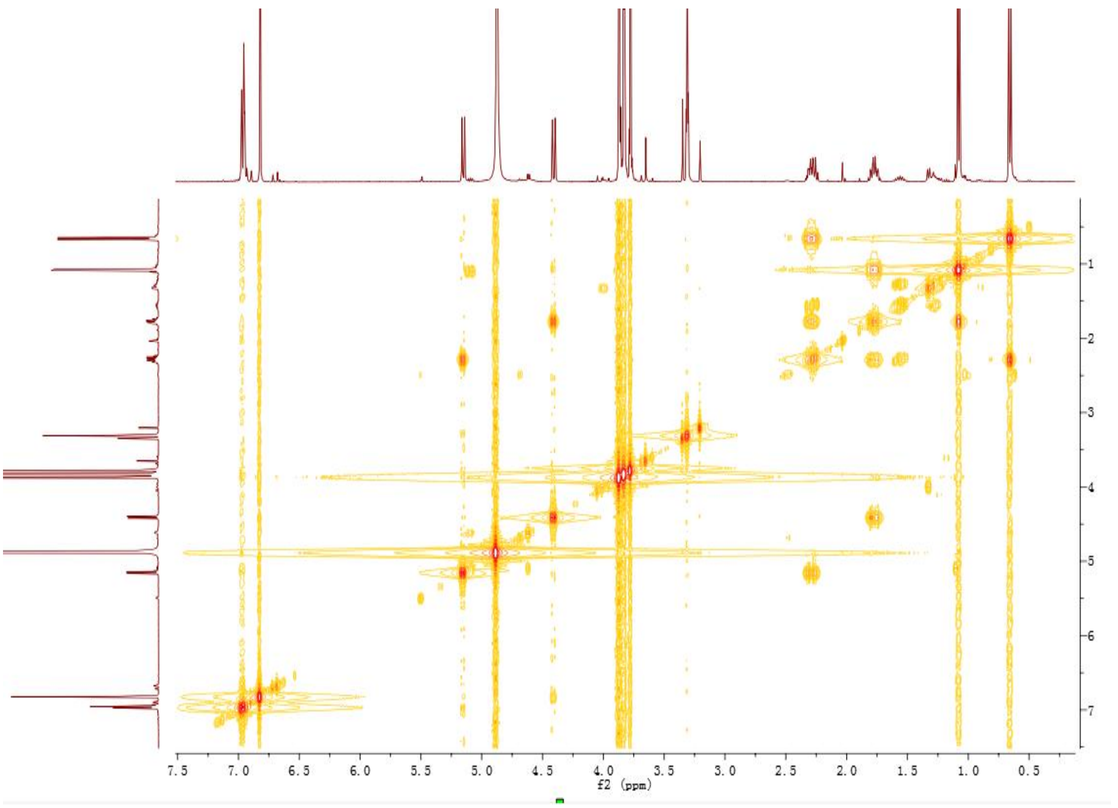

Figure S27. NOESY spectrum of **3** (**3a/3b**) in CD_3OD

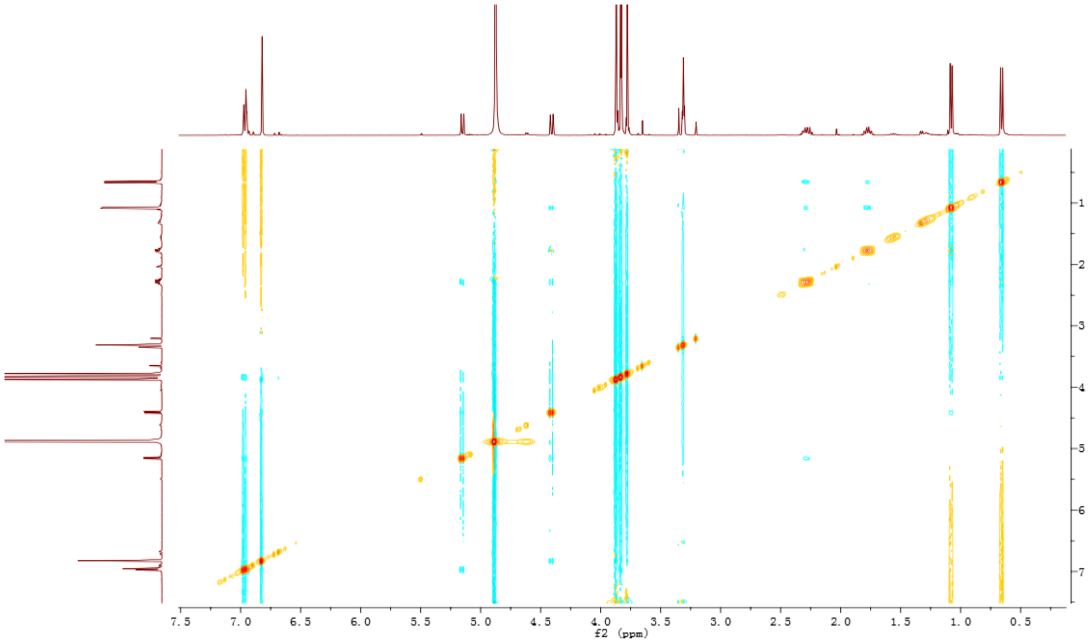

Figure S28. (+)-HR-ESI-MS spectrum of **4**

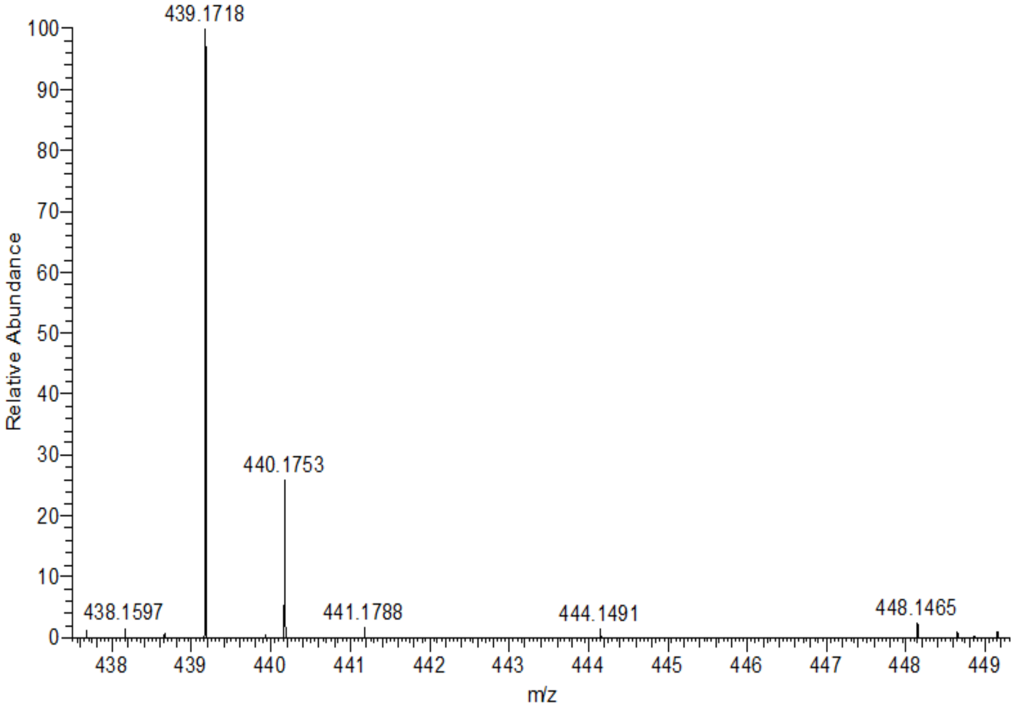

Figure S29. IR spectrum of **4**

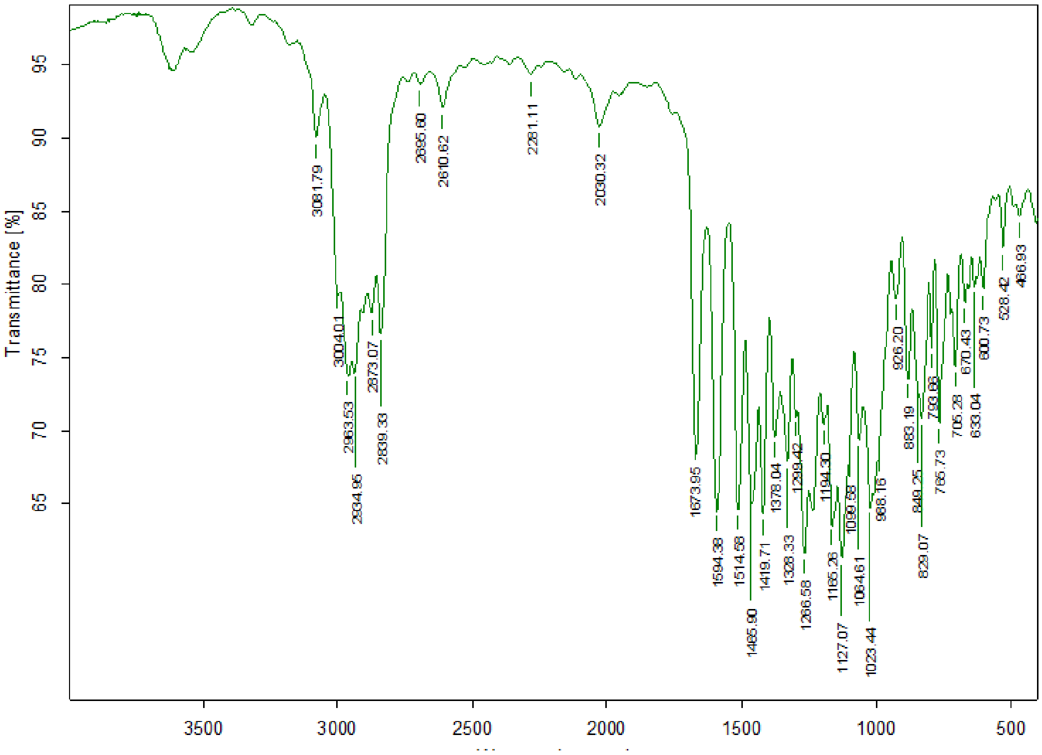

Figure S30. UV spectrum of **4**

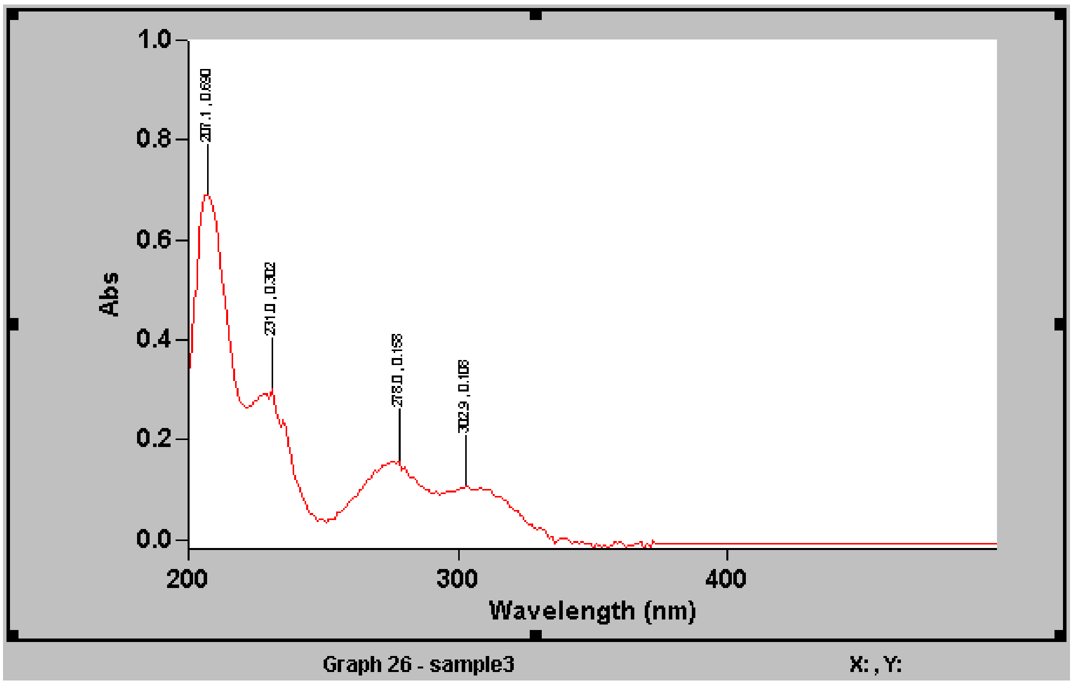

Figure S31. ^{1}H NMR spectrum of **4** in $CDCl_3$

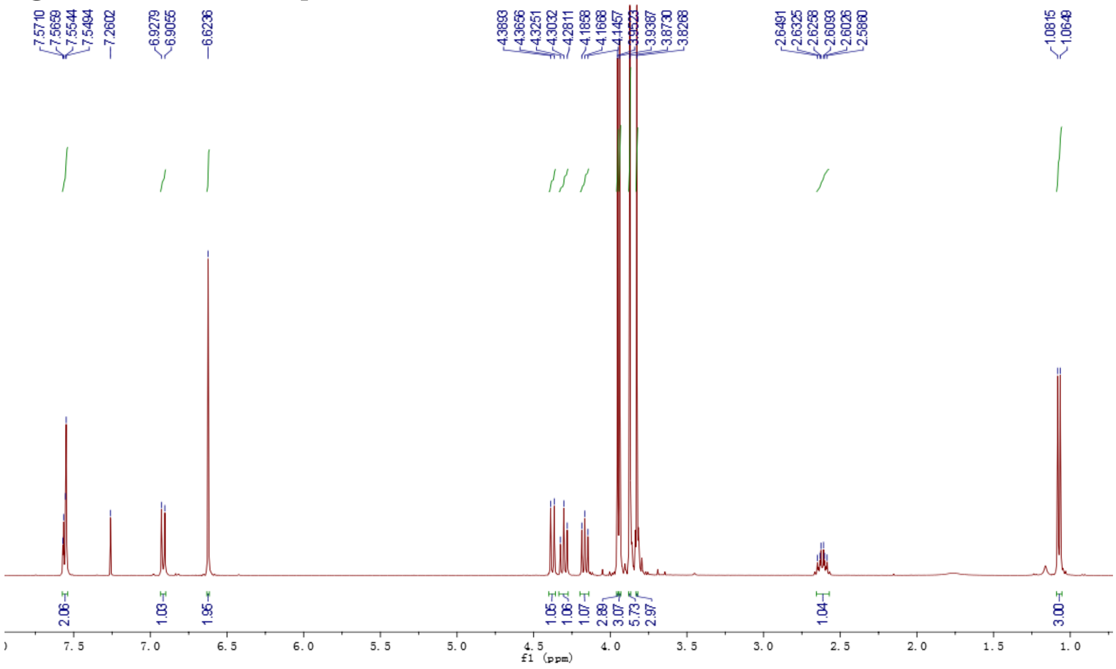

Figure S32. ^{13}C NMR spectrum of **4** in $CDCl_3$

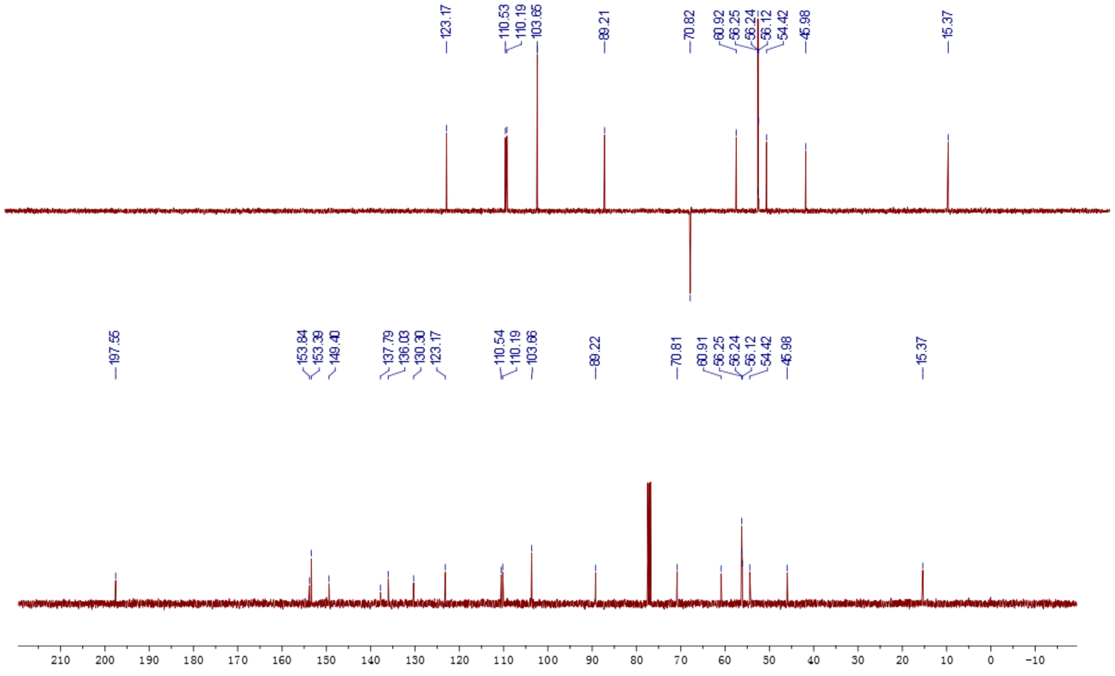

Figure S33. HSQC spectrum of **4** in $CDCl_3$

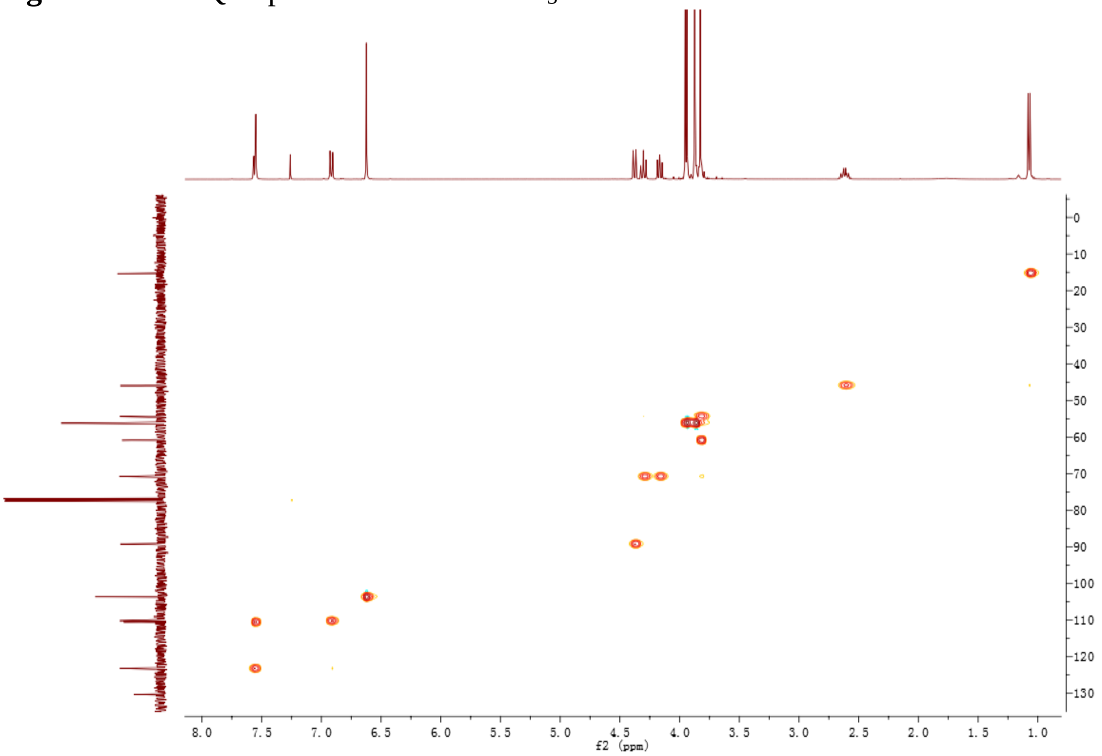

Figure S34. HMBC spectrum of **4** in $CDCl_3$

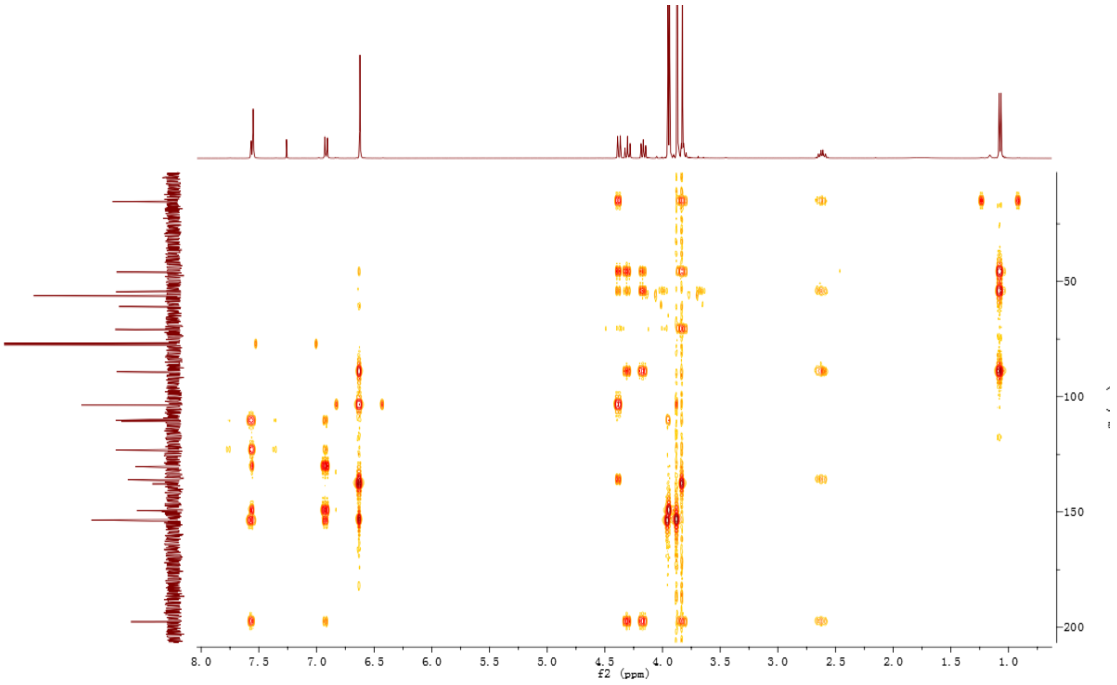

Figure S35. ^{1}H-^{1}H COSY spectrum of **4** in $CDCl_3$

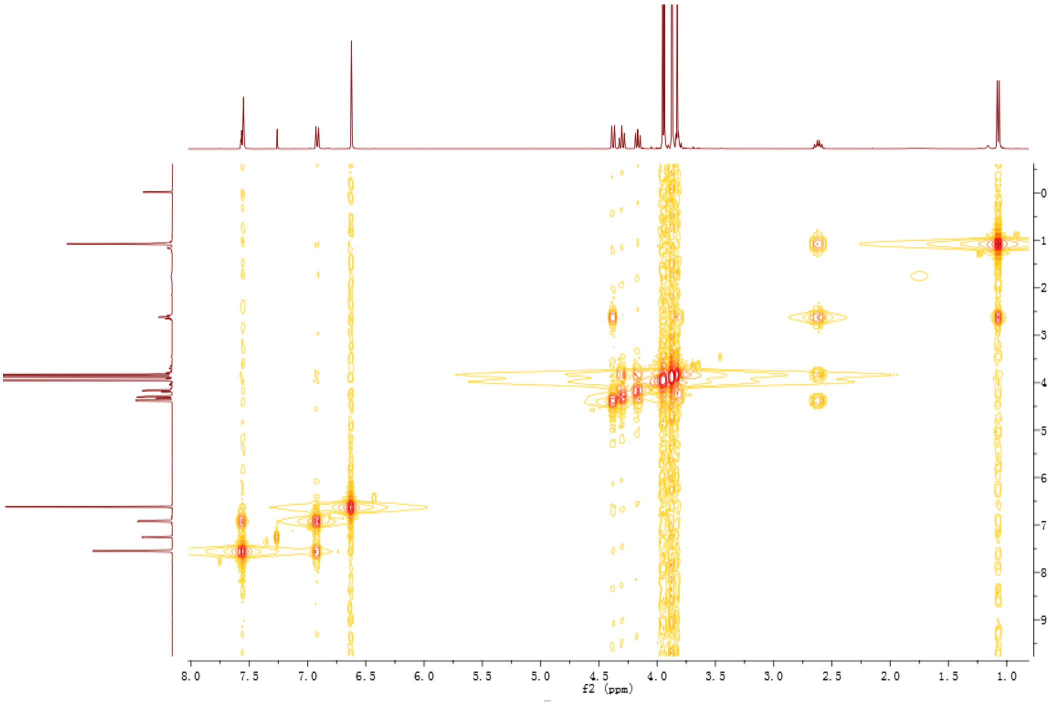

Figure S36. NOESY spectrum of **4** in $CDCl_3$

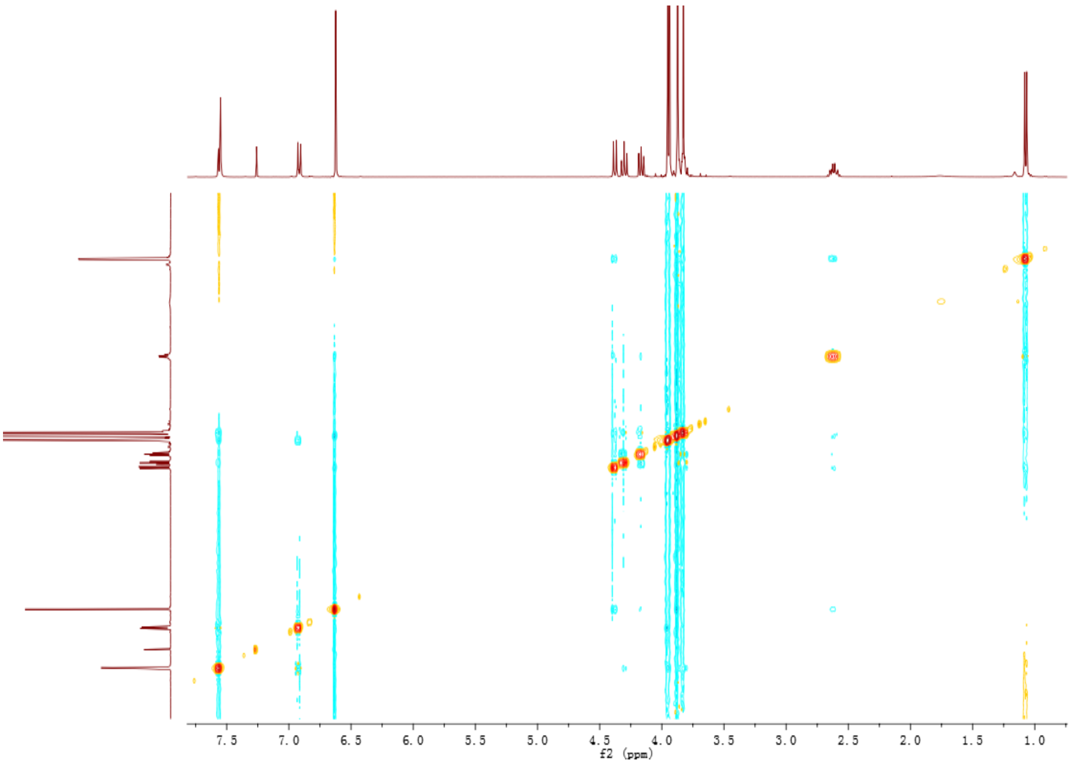

Figure S37. ECD spectrum of **4** in MeOH

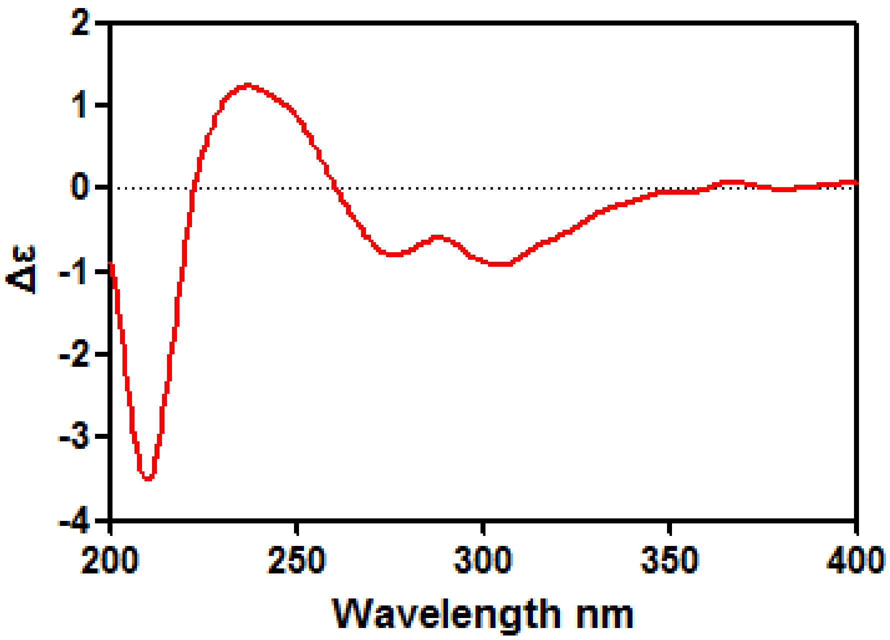

Figure S38. (+)-HR-ESI-MS spectrum of **5**

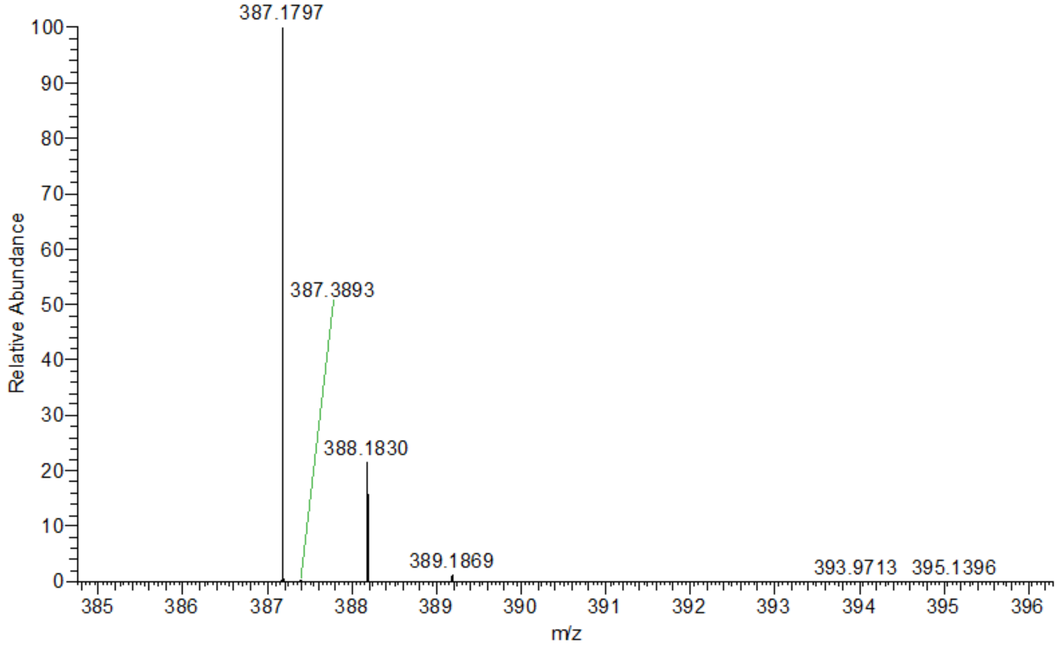

Figure S39. IR spectrum of **5**

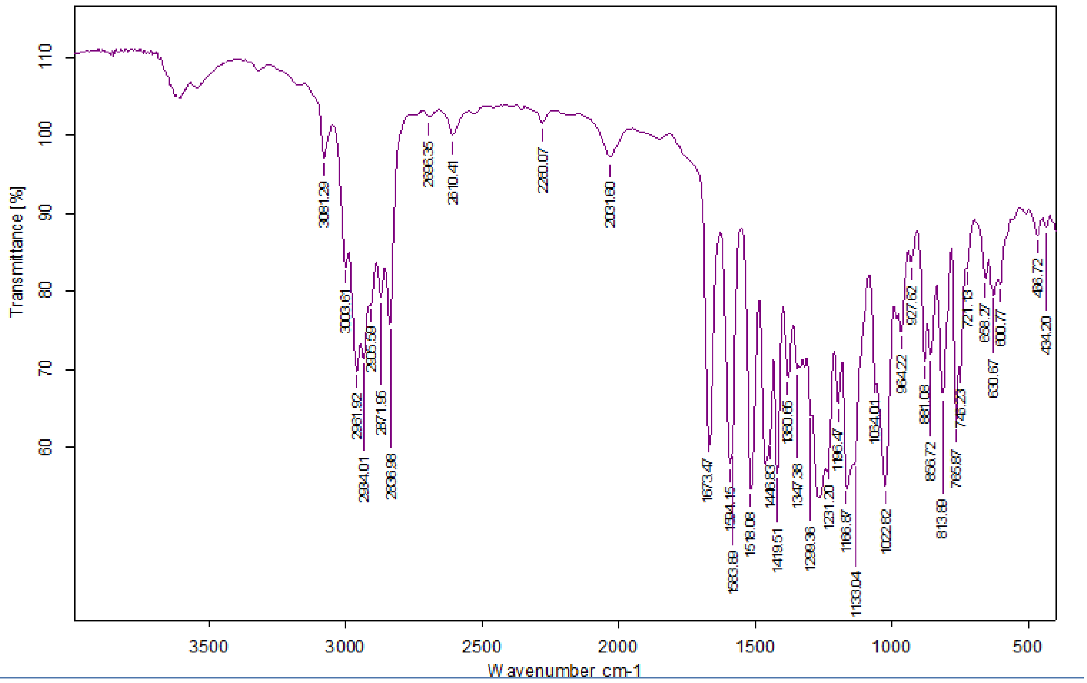

Figure S40. UV spectrum of **5**

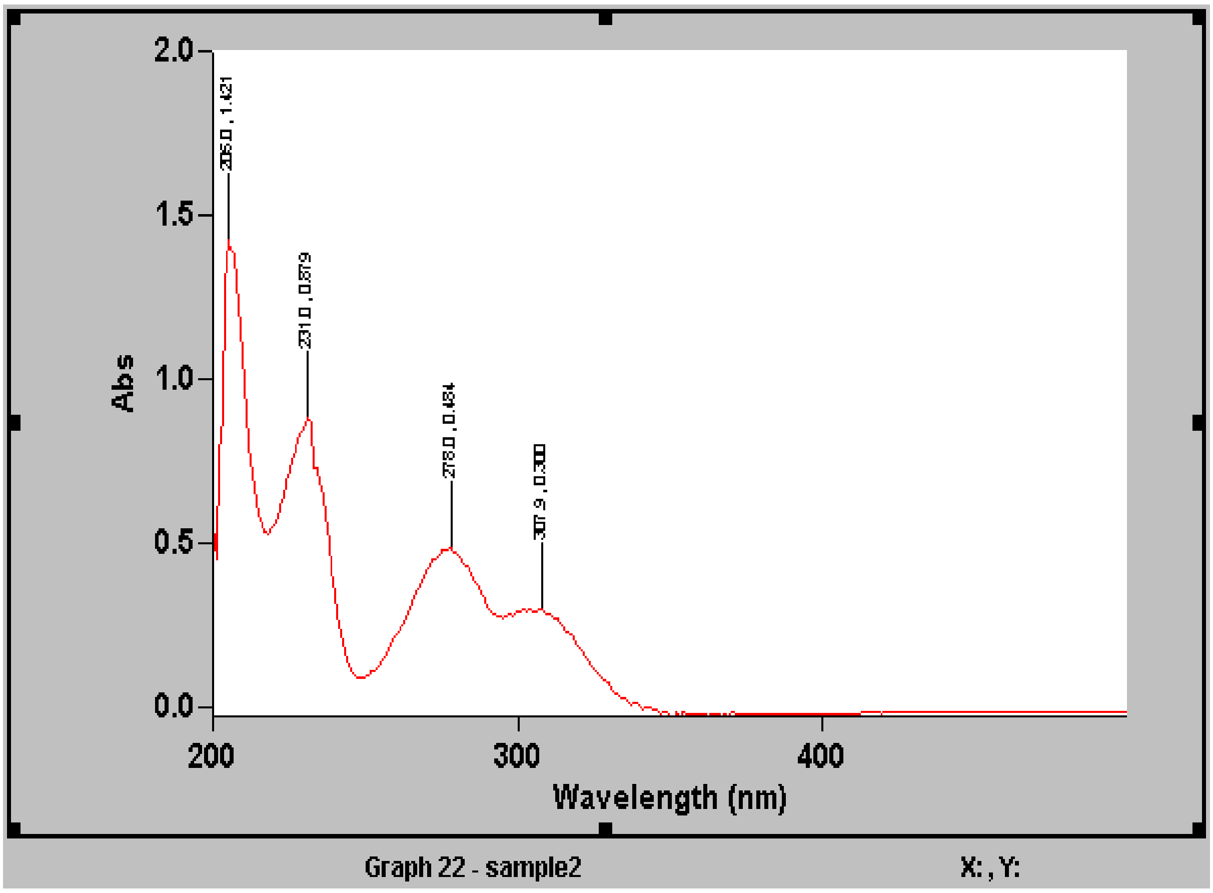

Figure S41. ^{1}H NMR spectrum of **5** in CD_3OD

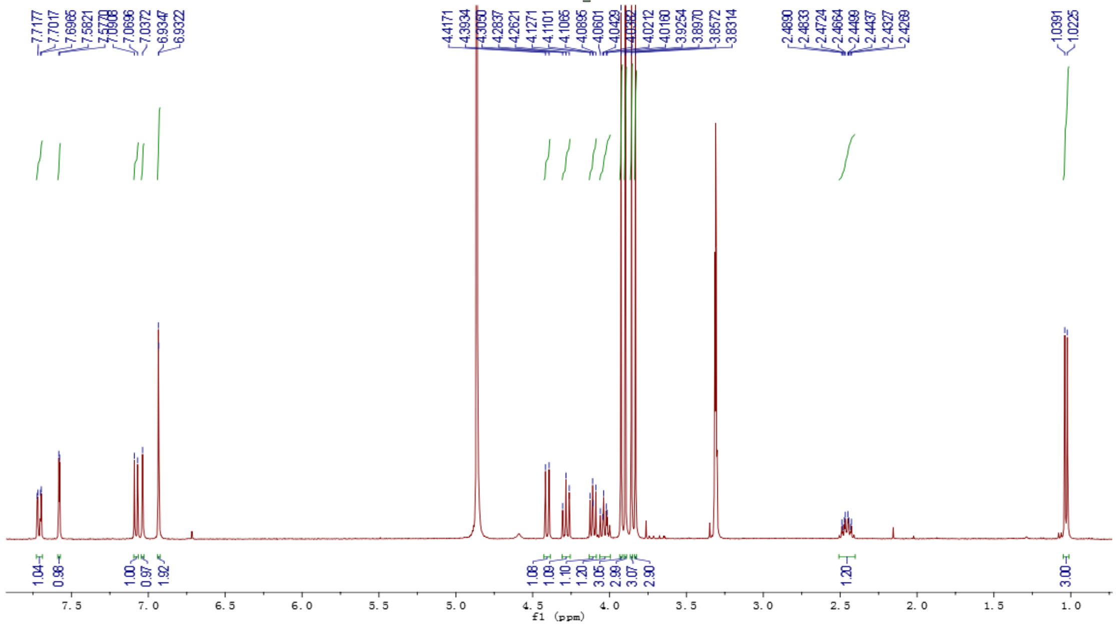

Figure S42. ^{13}C NMR spectrum of **5** in CD_3OD

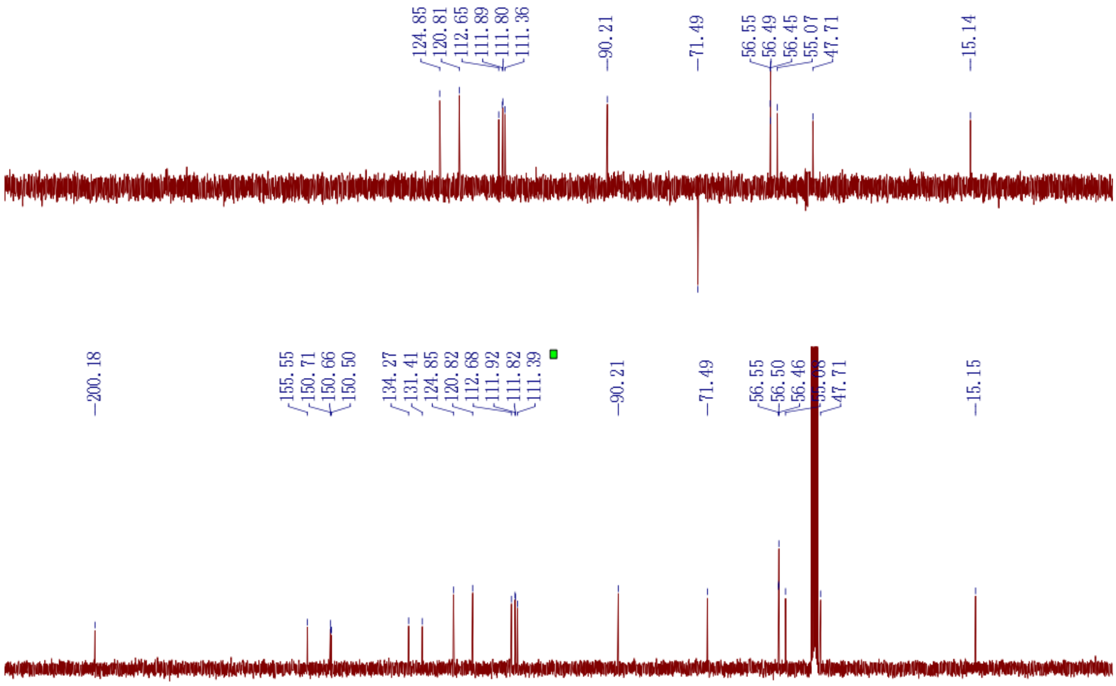

Figure S43. HSQC spectrum of **5** in CD_3OD

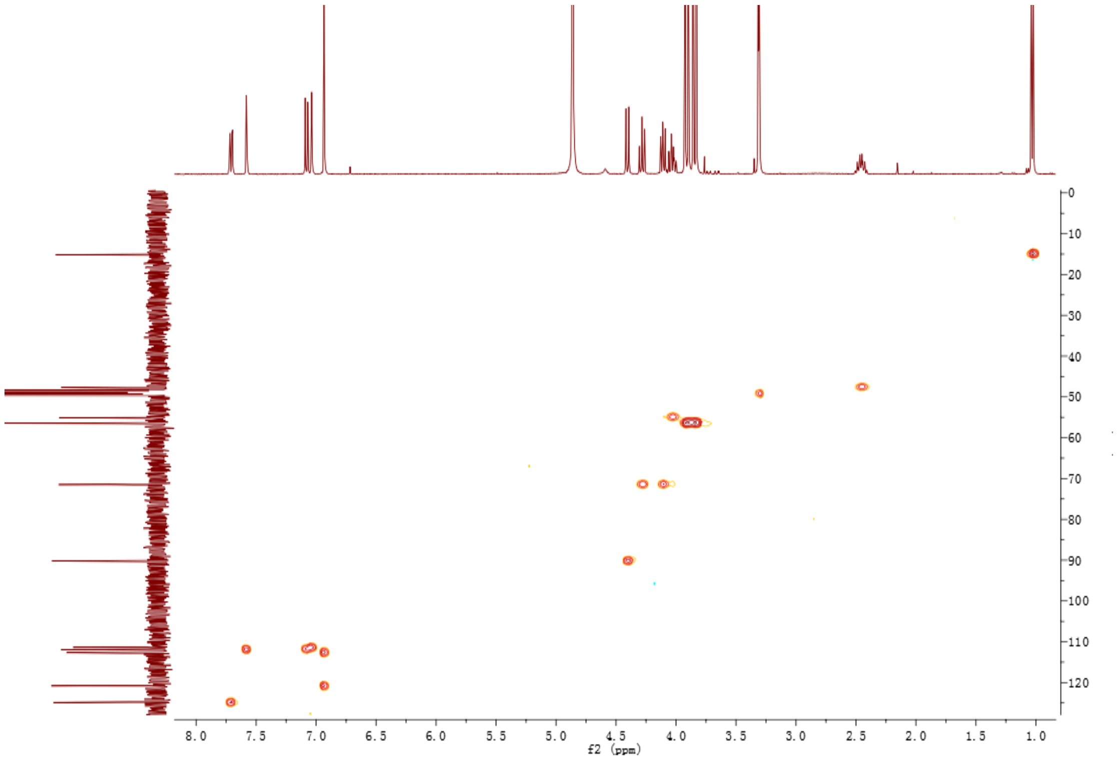

Figure S44. HMBC spectrum of **5** in CD_3OD

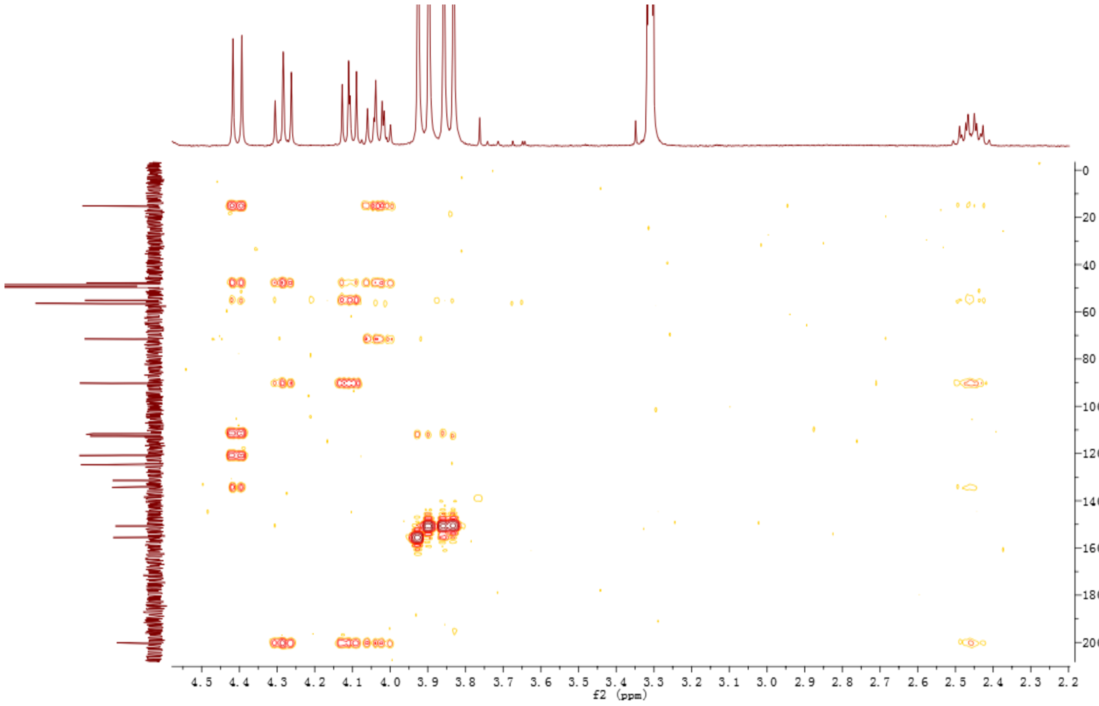

Figure S45. ^{1}H-^{1}H COSY spectrum of **5** in CD_3OD

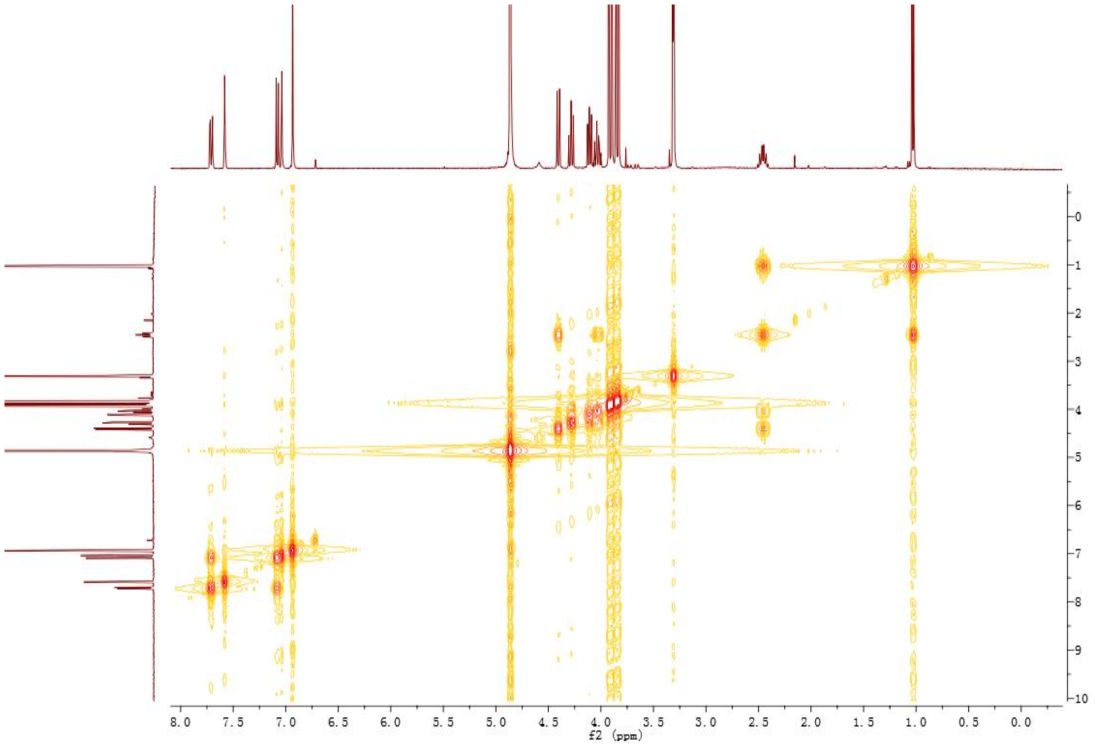

Figure S46. NOSEY spectrum of **5** in CD_3OD

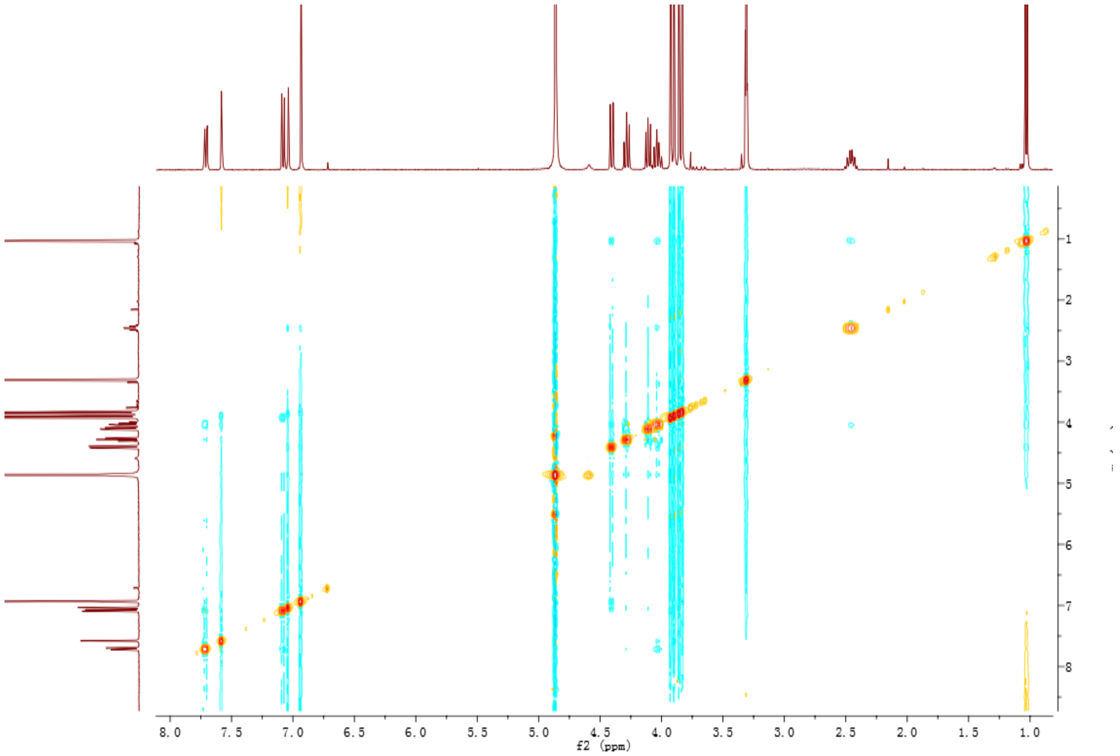

Figure S47. ECD spectrum of **5** in MeOH

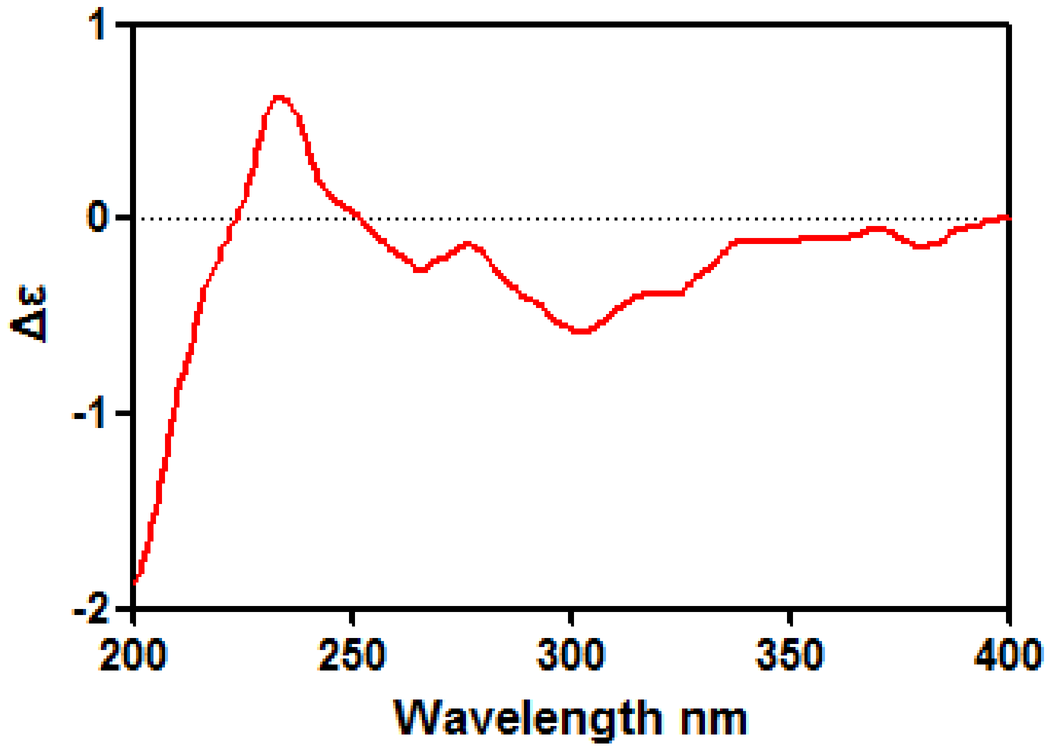

Figure S48. (+)-HR-ESI-MS spectrum of **6**

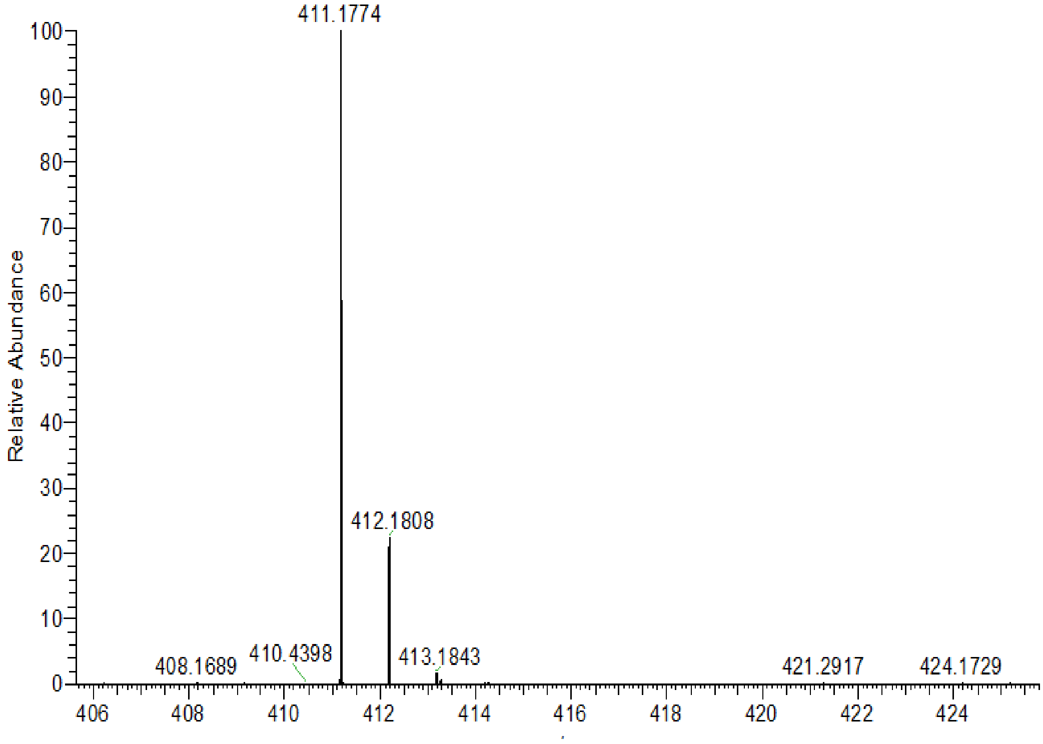

Figure S49. IR spectrum of **6** (**6a/6b**)

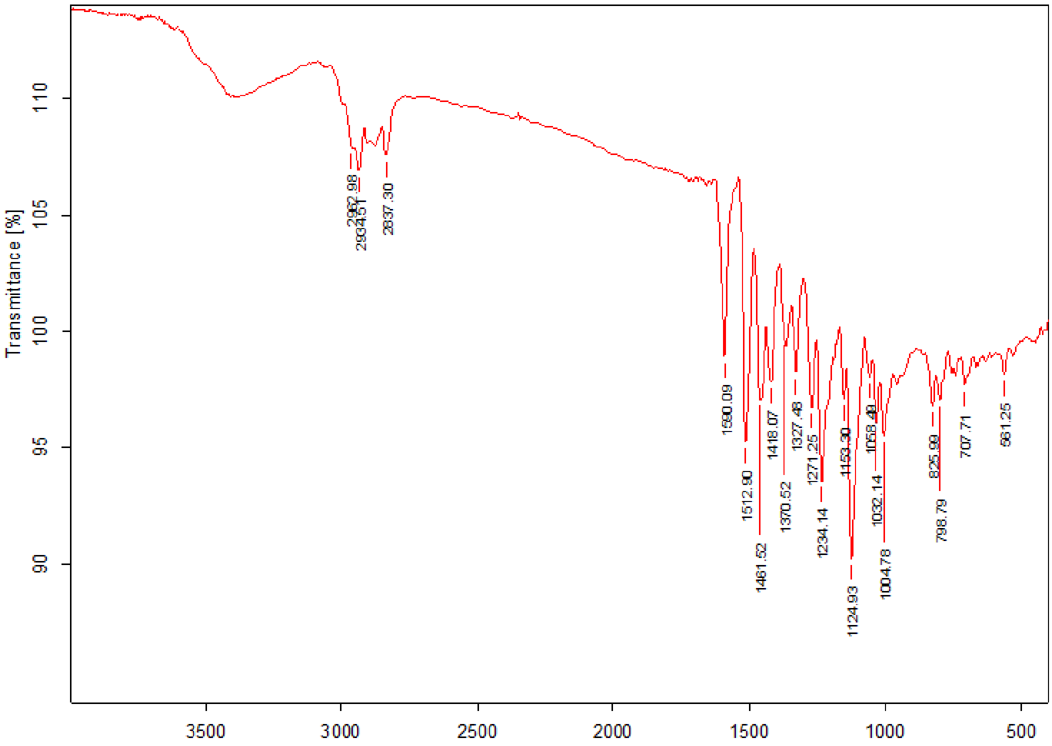

Figure S50. UV spectrum of **6**

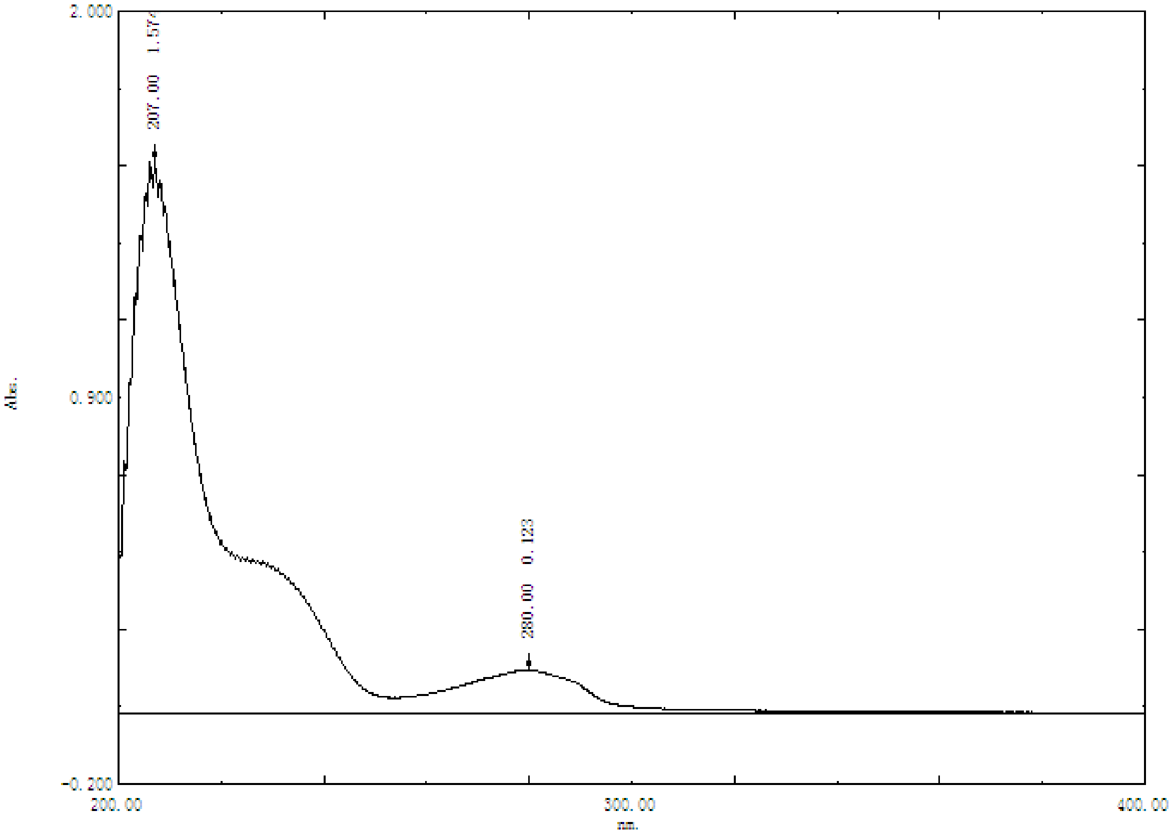

Figure S51. ^{1}H NMR spectrum of **6** in CD_3OD

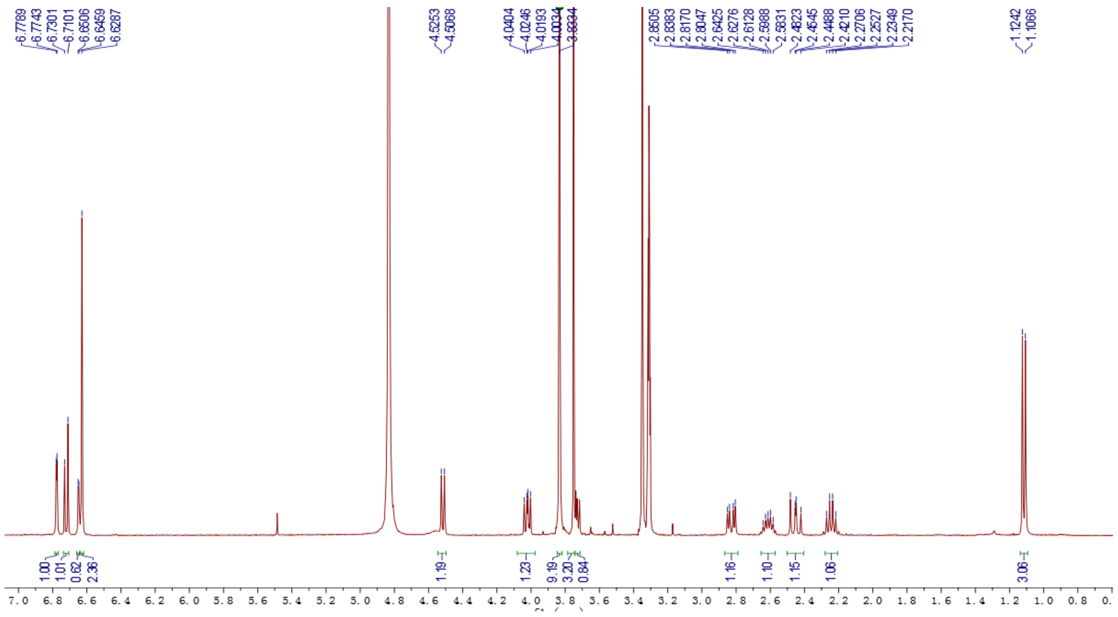

Figure S52. ^{13}C NMR spectrum of **6** in CD_3OD

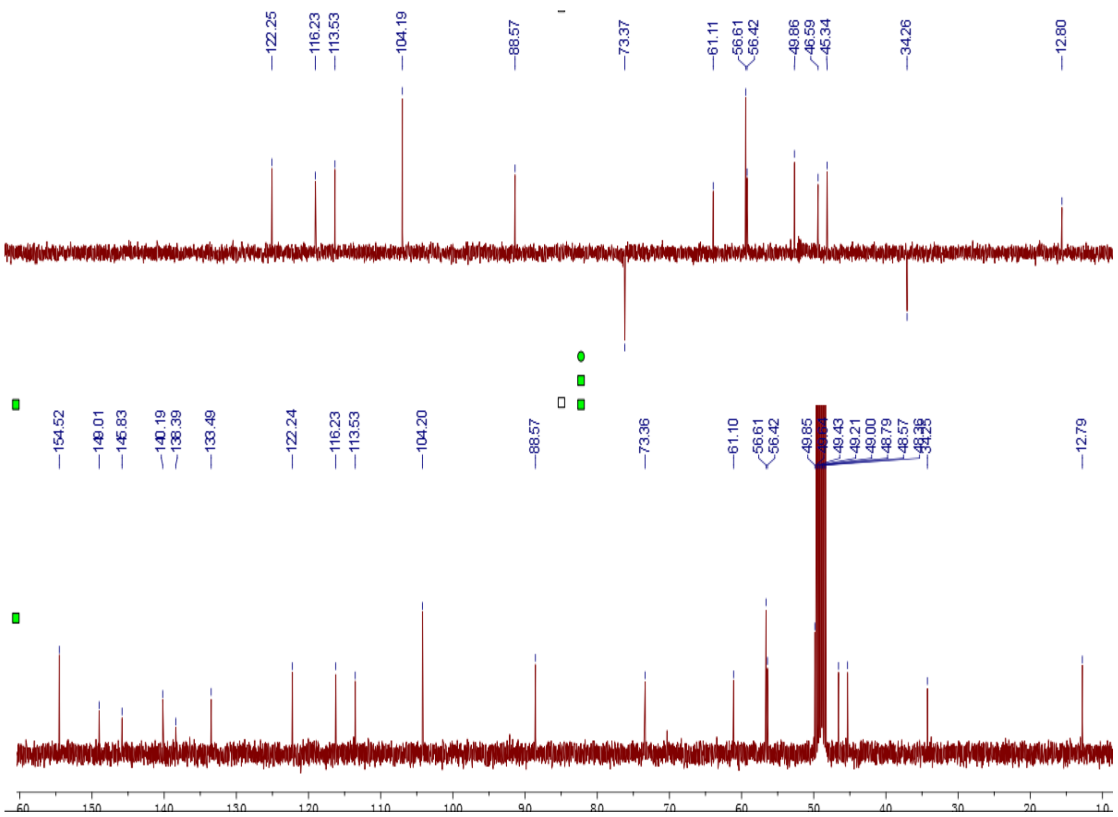

Figure S53. HSQC spectrum of **6** in CD_3OD

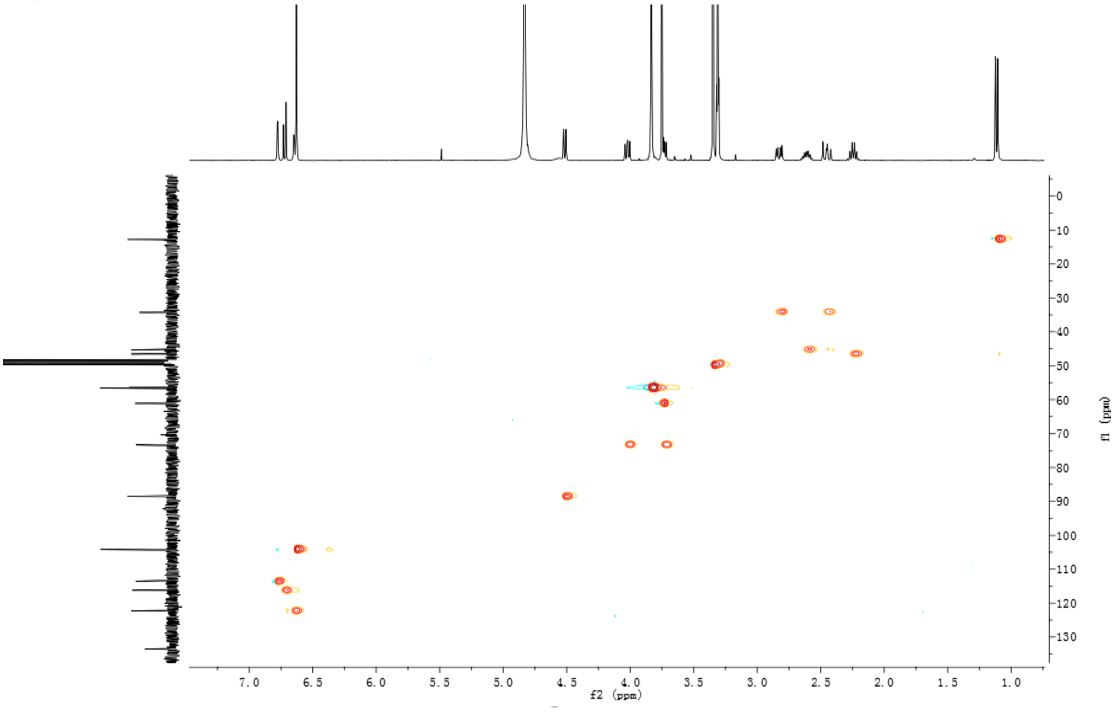

Figure S54. HMBC spectrum of **6** in CD_3OD

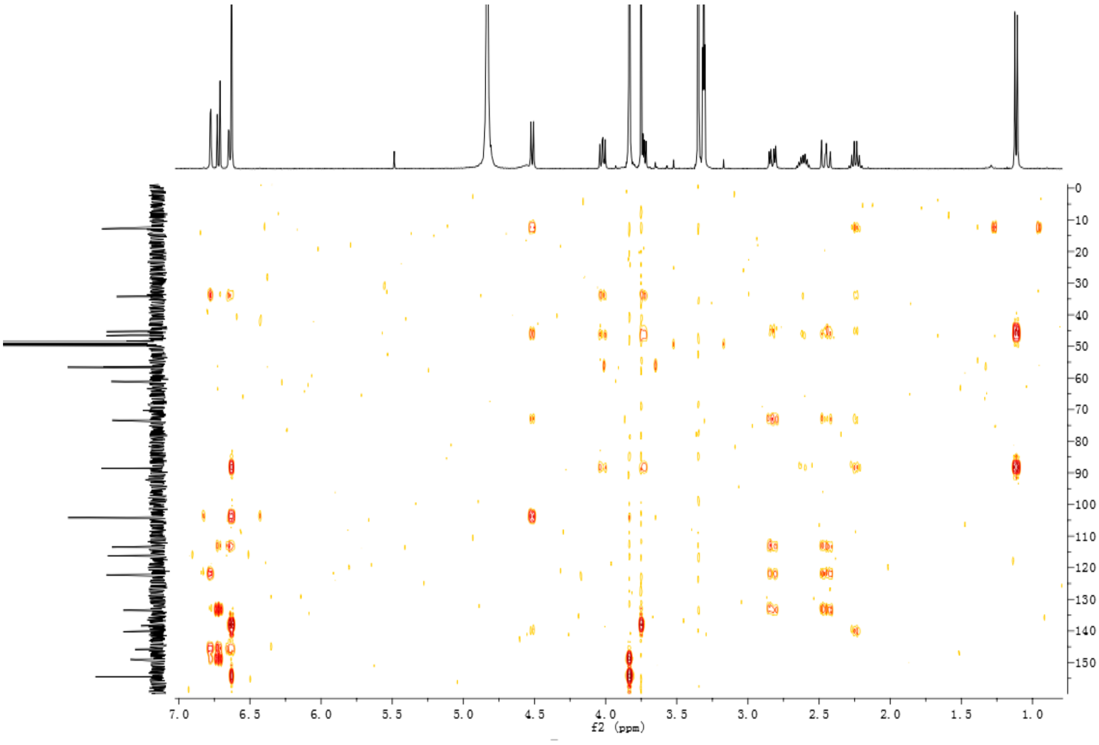

Figure S55. ^{1}H-^{1}H COSY spectrum of **6** in CD_3OD

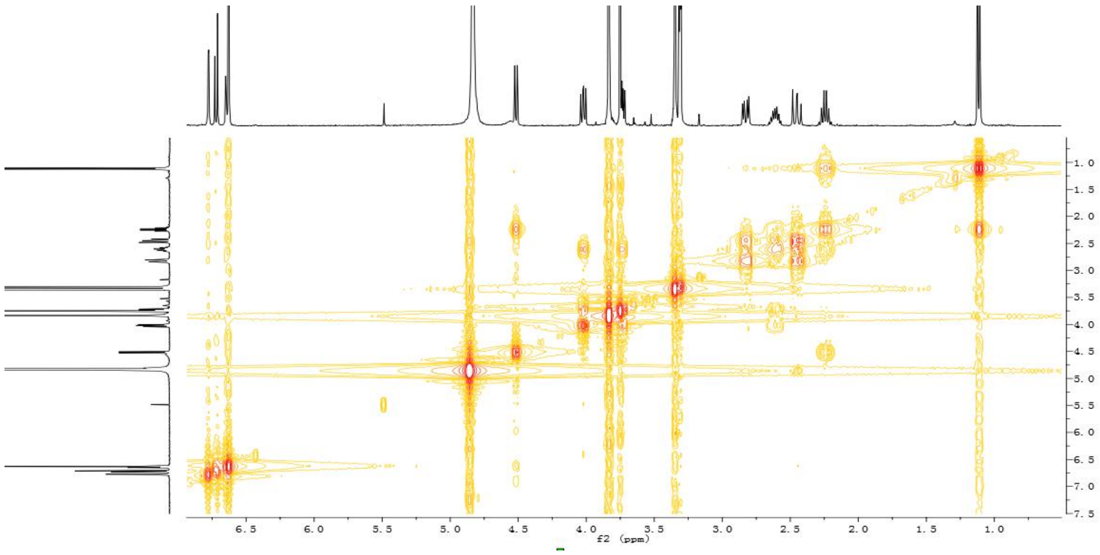

Figure S56. NOESY spectrum of **6** in CD_3OD

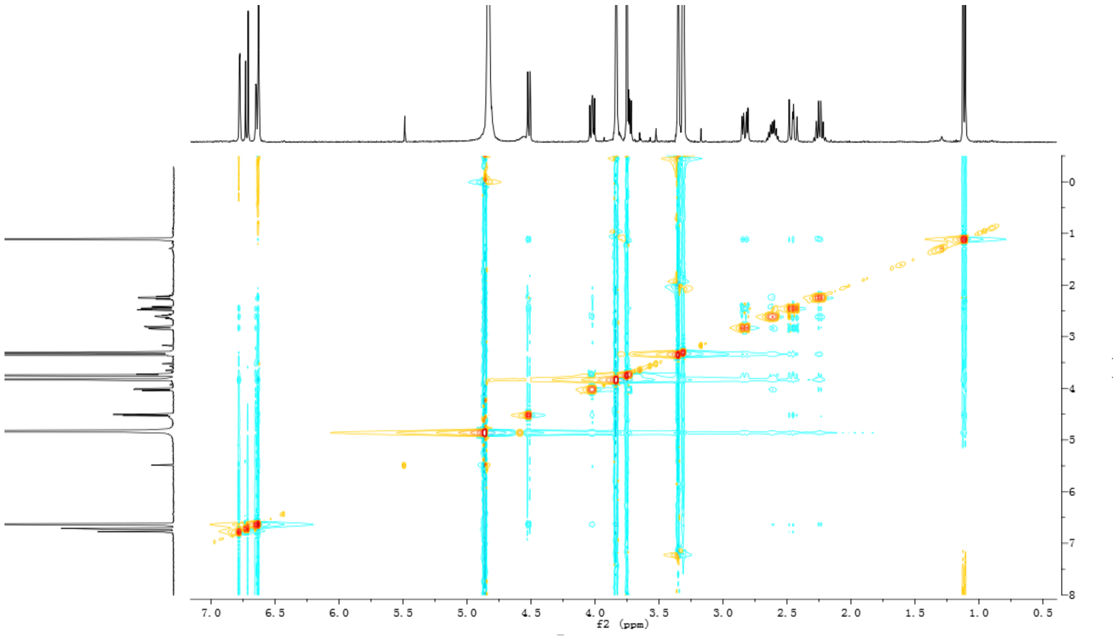

Figure S57. ECD spectrum of **6** in MeOH

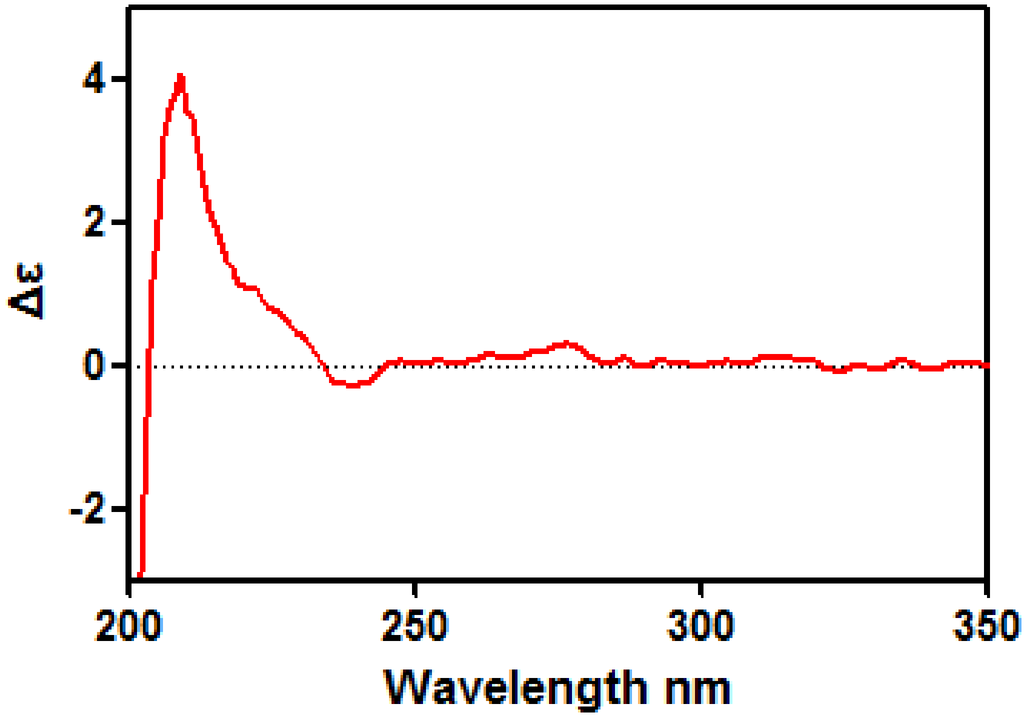

Figure S58. (+)-HR-ESI-MS spectrum of **7**

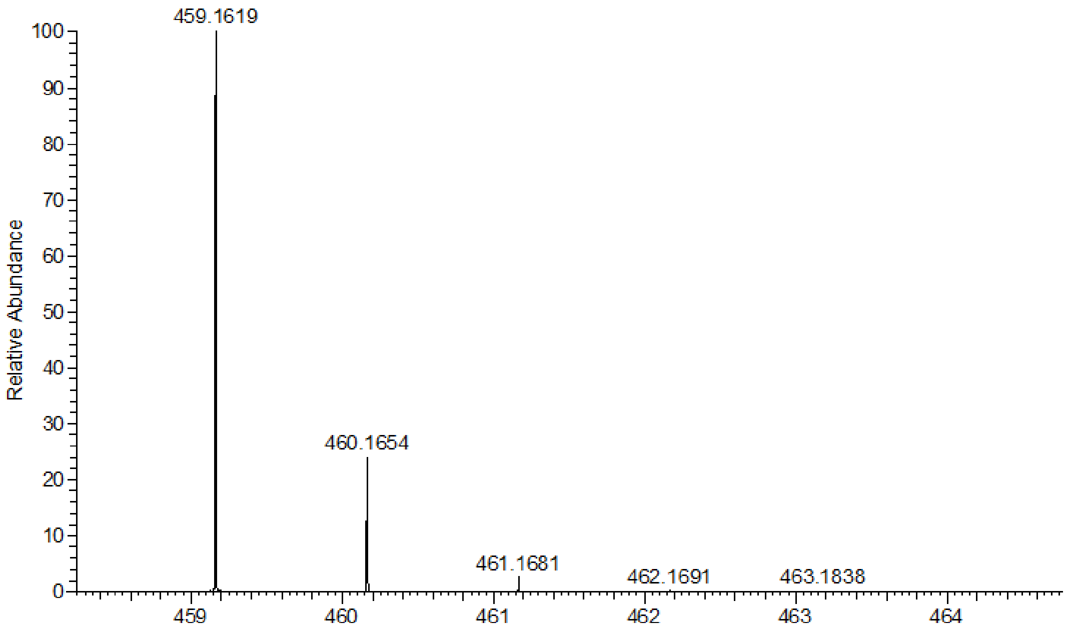

Figure S59. IR spectrum of **7**

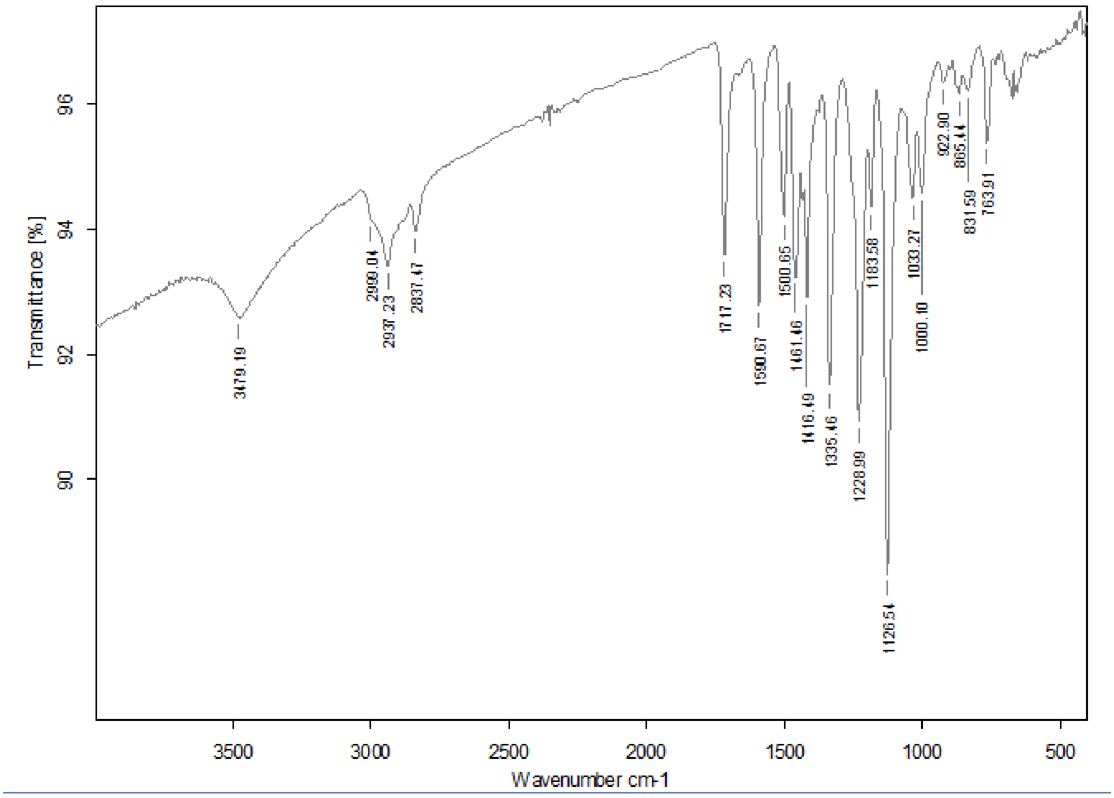

Figure S60. UV spectrum of **7**

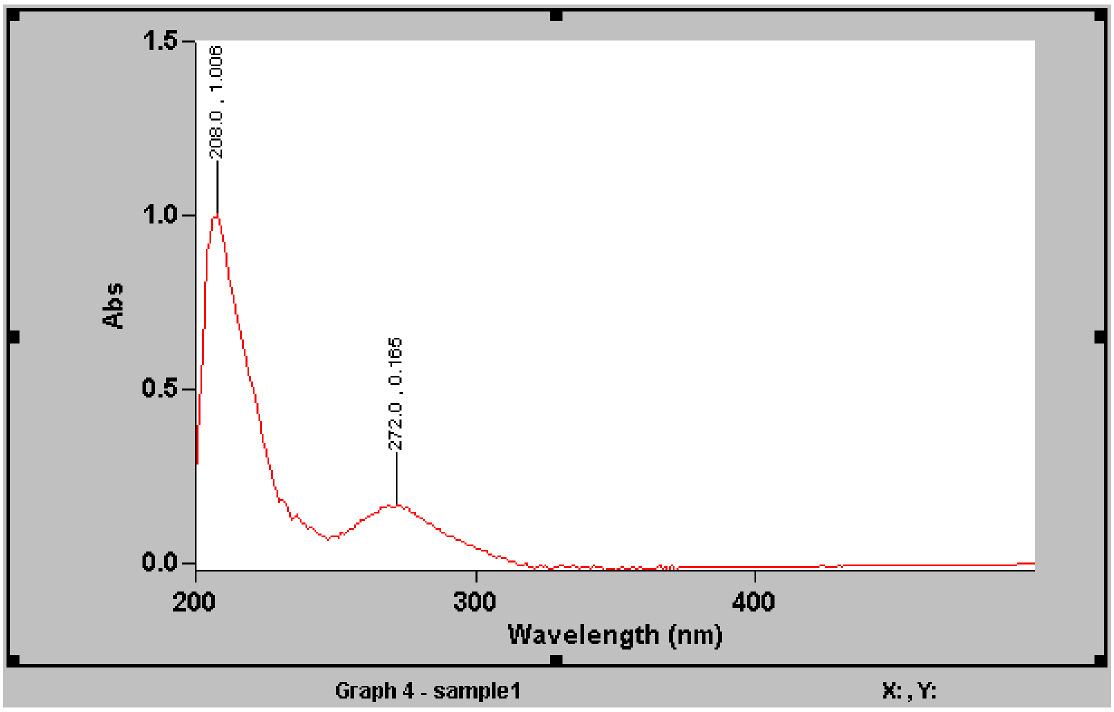

Figure S61. ^{1}H NMR spectrum of **7** in CD_3OD

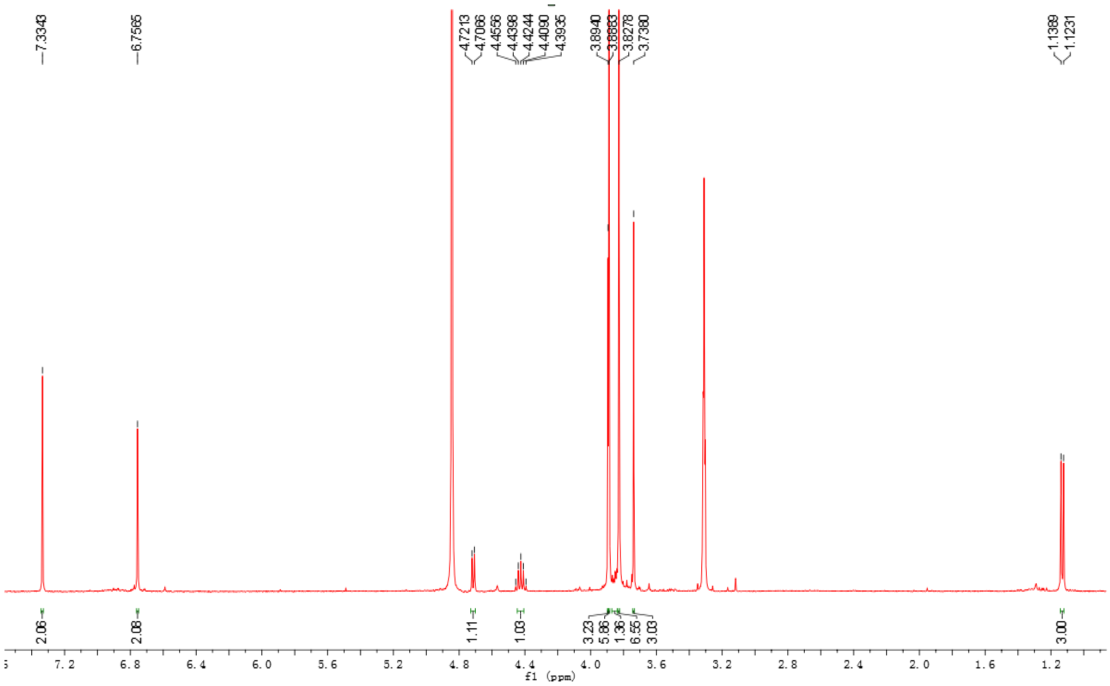

Figure S62. ^{13}C NMR spectrum of **7** in CD_3OD

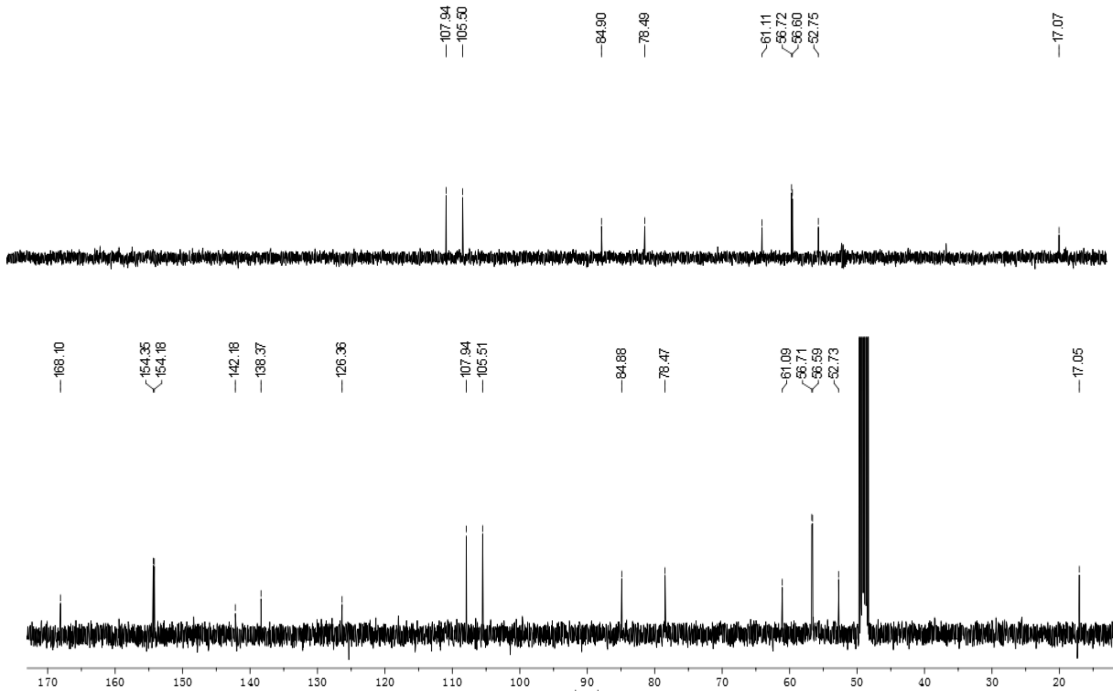

Figure S63. HSQC spectrum of **7** in CD_3OD

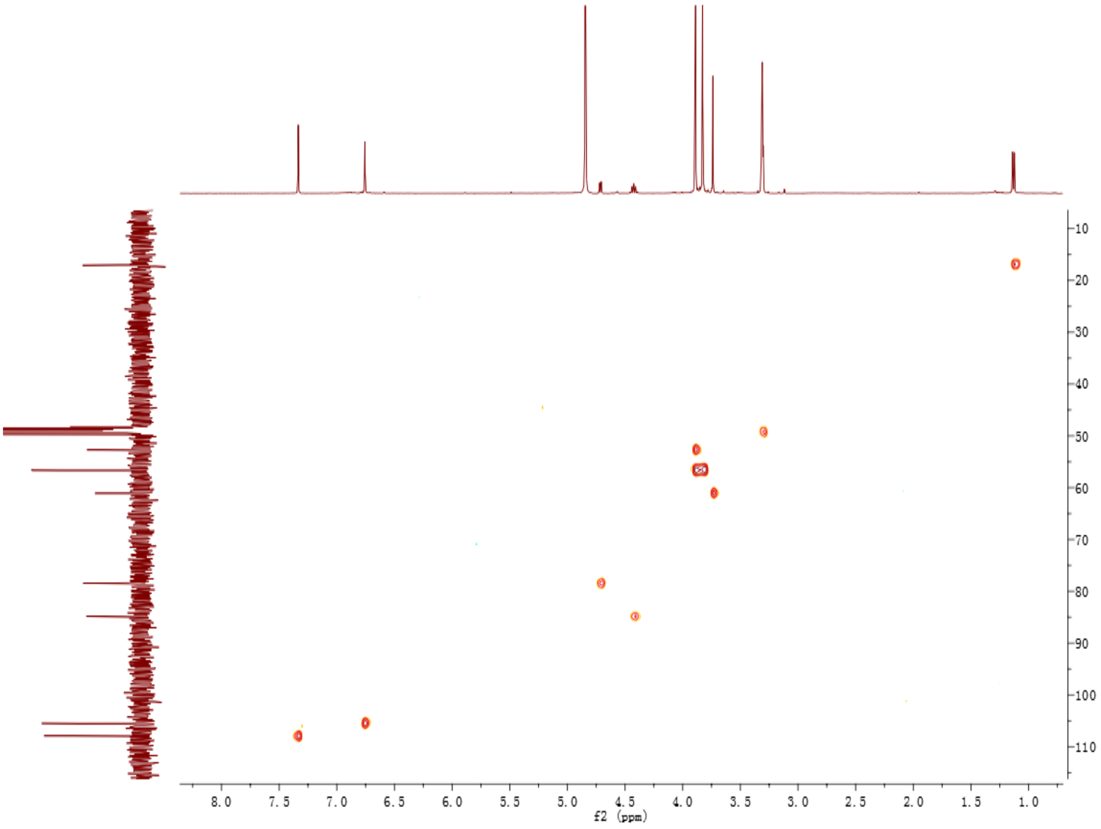

Figure S64. HMBC spectrum of **7** in CD_3OD

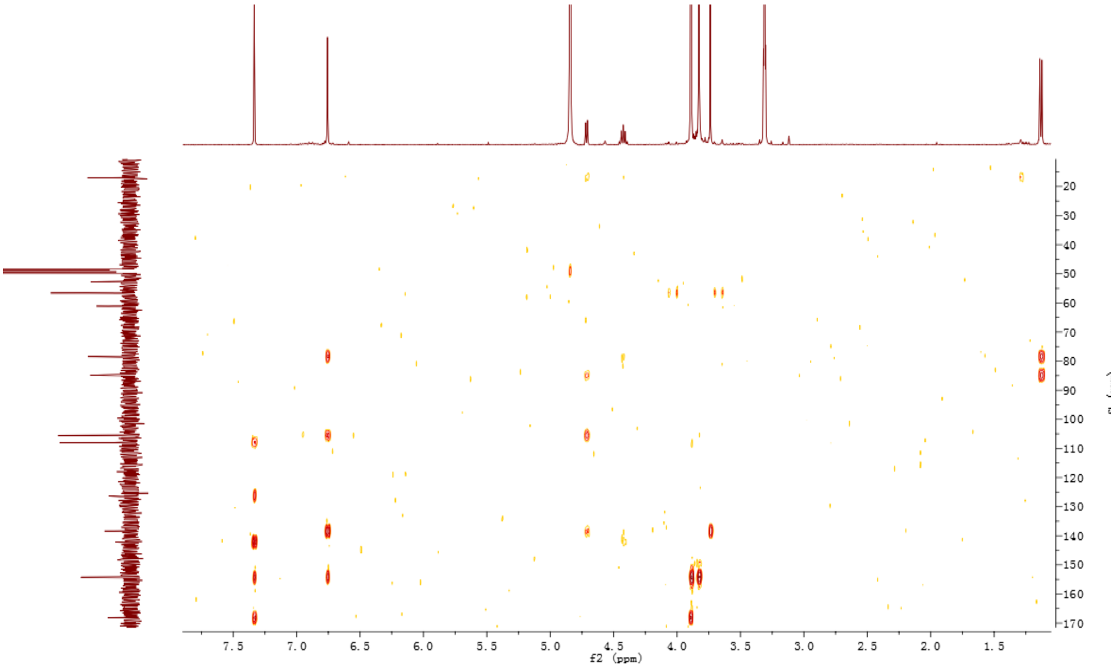

Figure S65. ${}^{1}H$-${}^{1}H$ COSY spectrum of **7** in CD_3OD

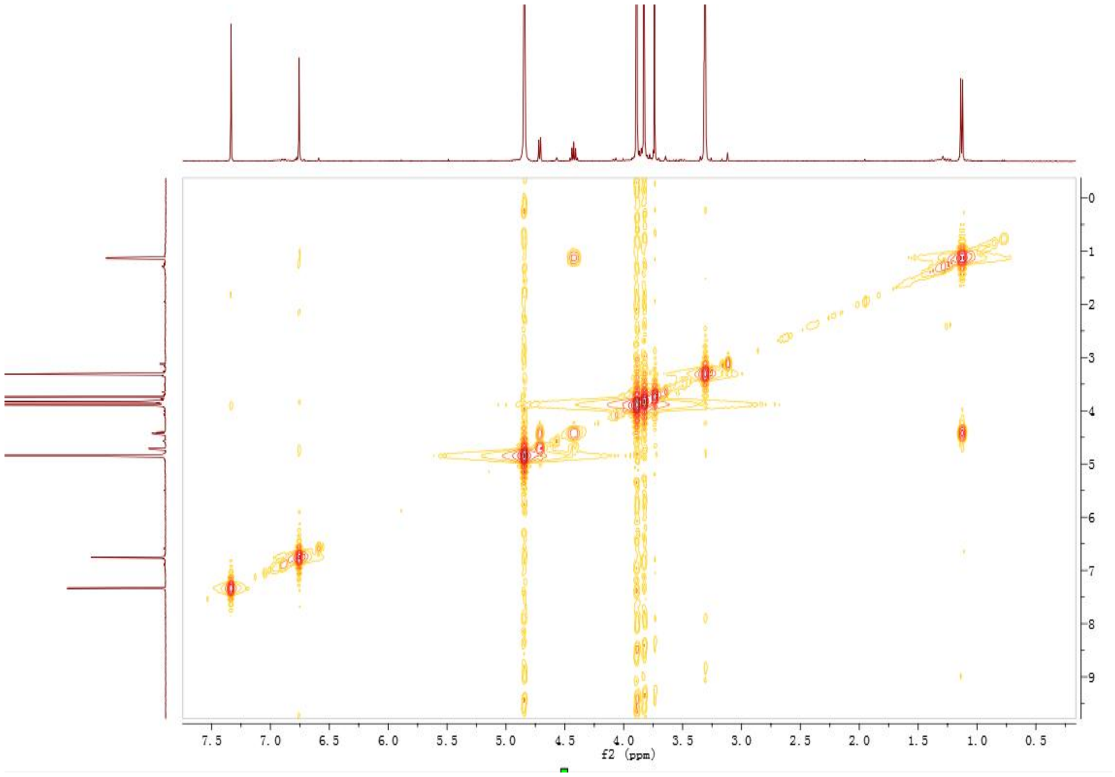

Figure S66. NOESY spectrum of **7** in CD_3OD

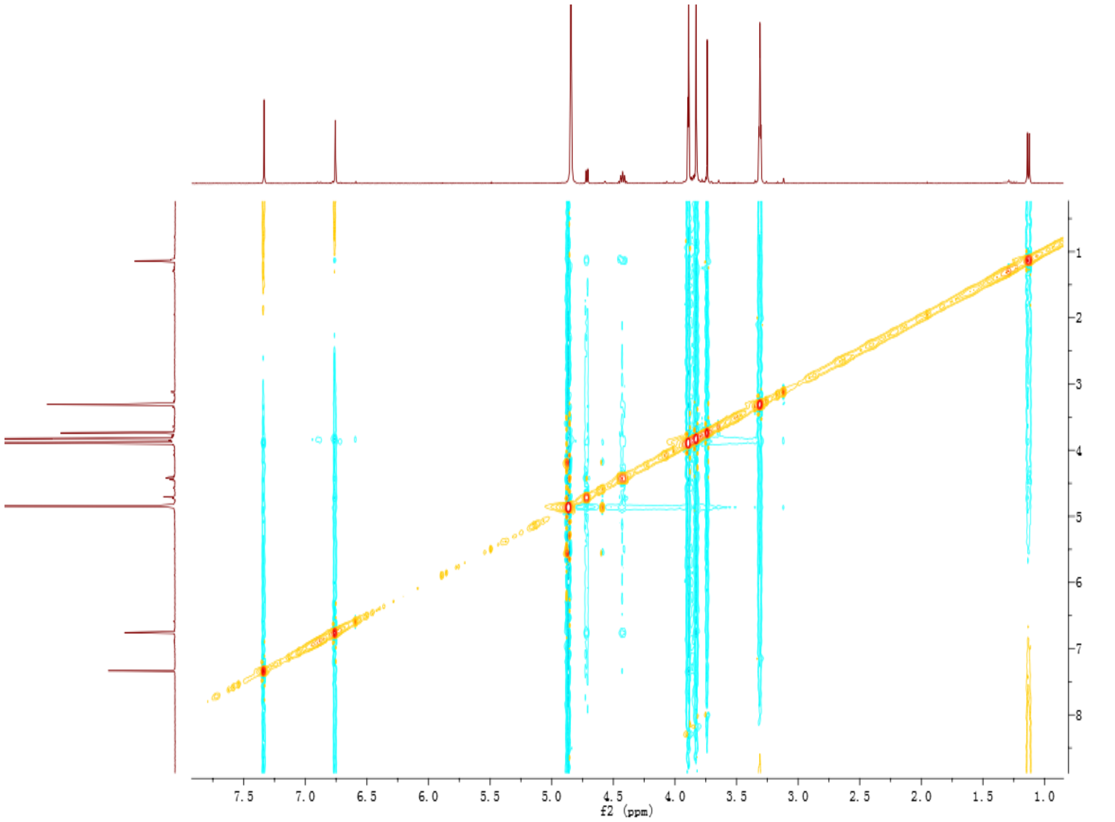

Figure S67. ECD spectrum of **7** in MeOH

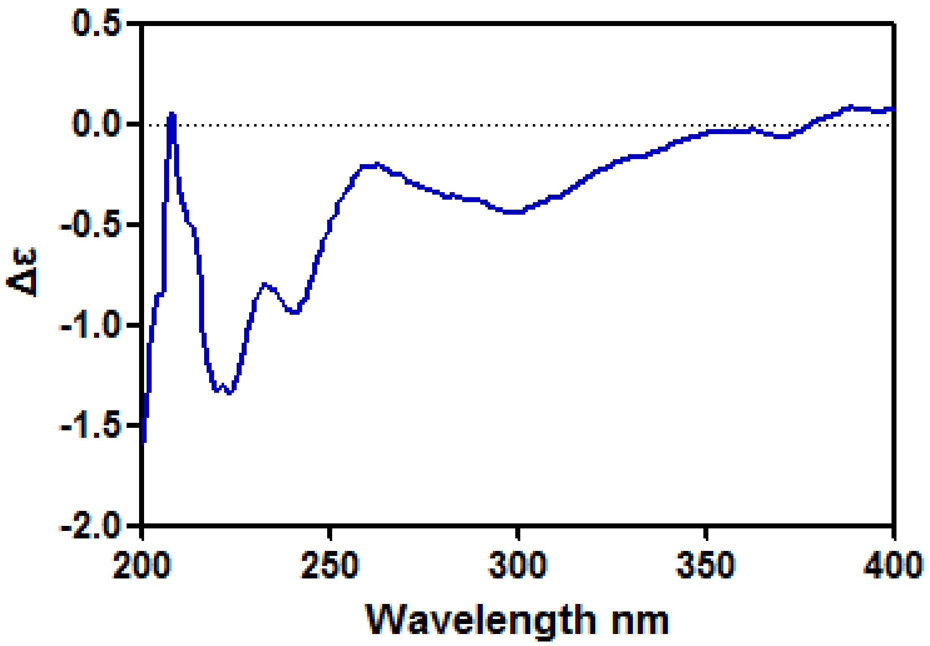

Figure S68. (+)-HR-ESI-MS spectrum of **8**

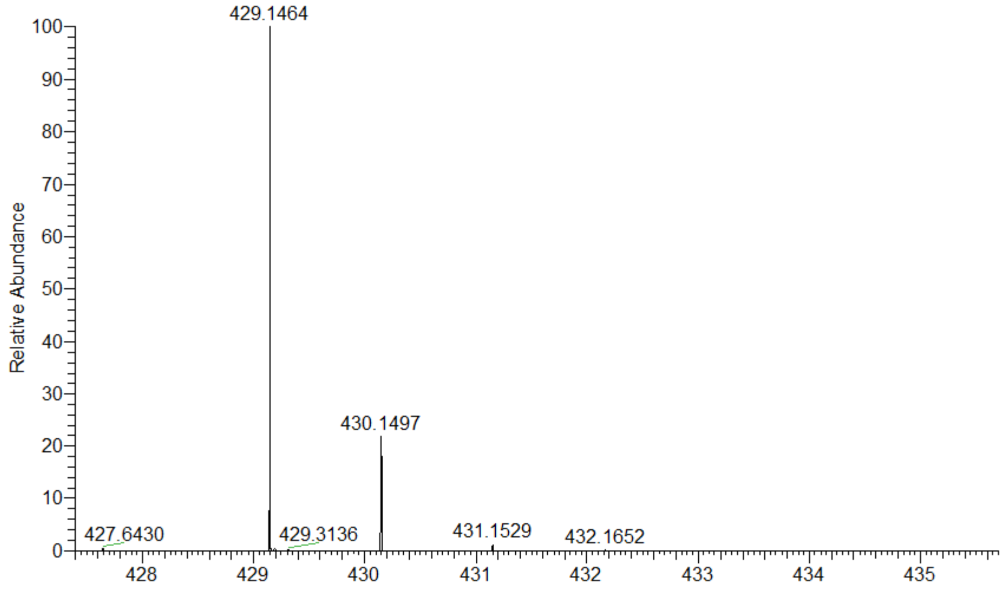

Figure S69. IR spectrum of **8**

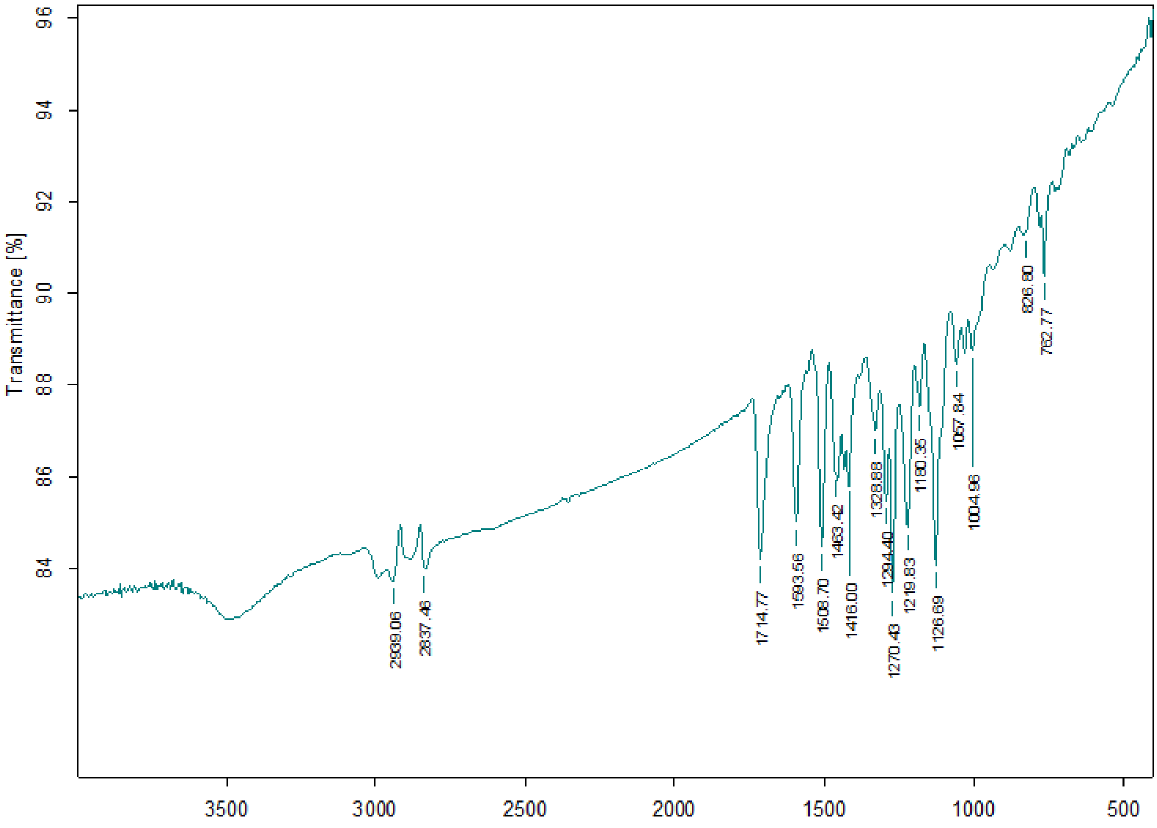

Figure S70. UV spectrum of **8**

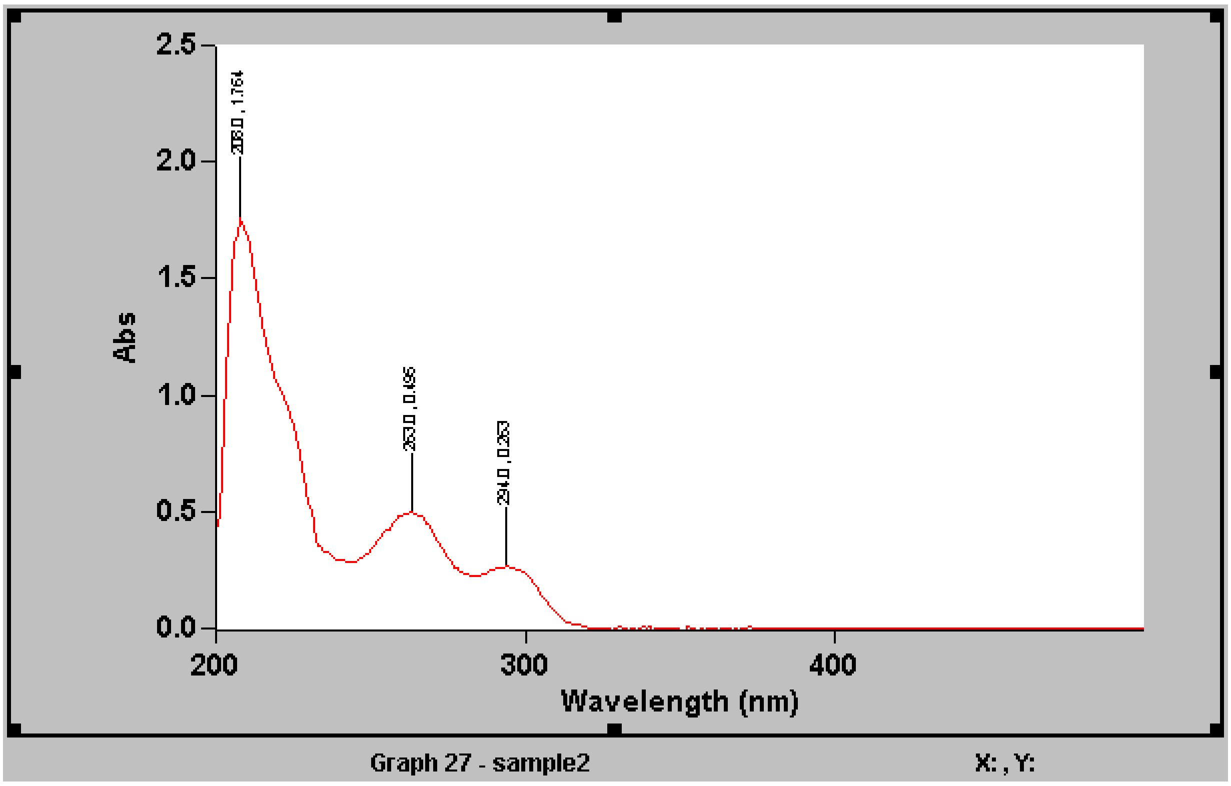

Figure S71. ^{1}H NMR spectrum of **8** in CD_3OD

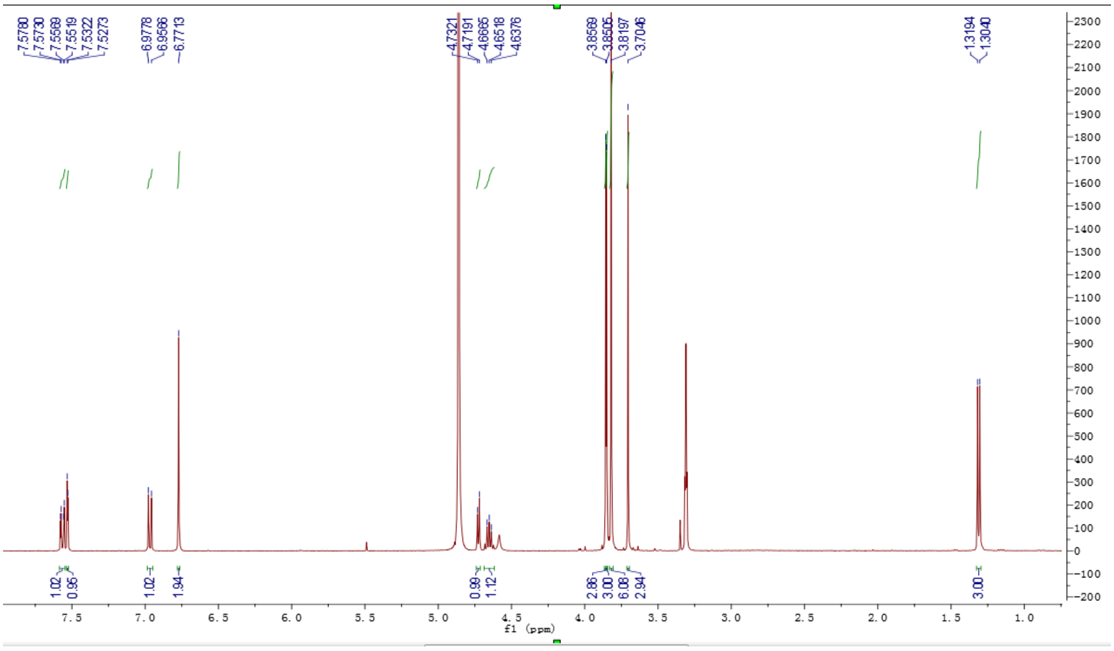

Figure S72. ^{13}C NMR spectrum of **8** in CD_3OD

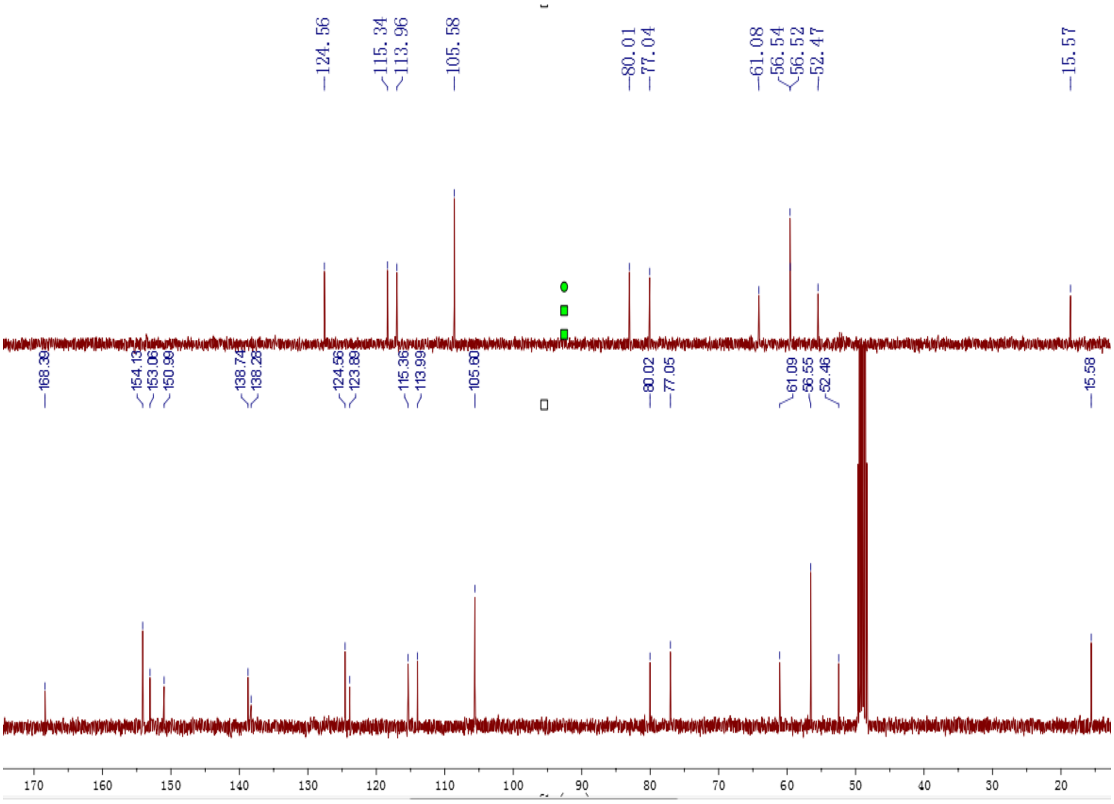

Figure S73. HSQC spectrum of **8** in CD_3OD

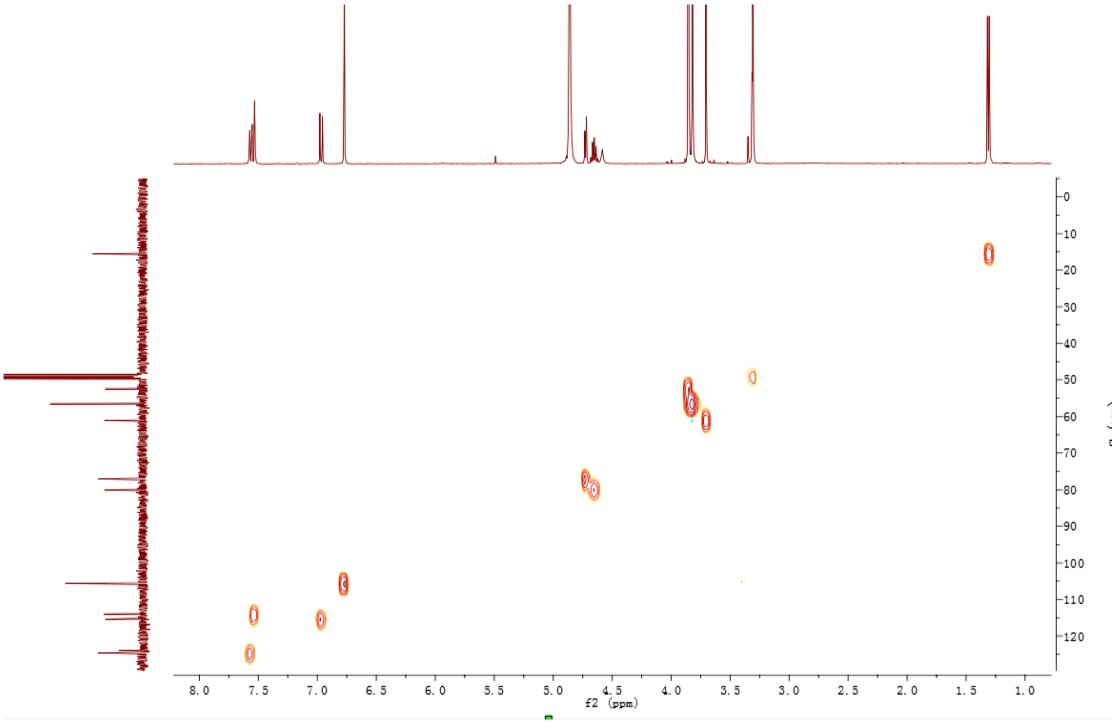

Figure S74. HMBC spectrum of **8** in CD_3OD

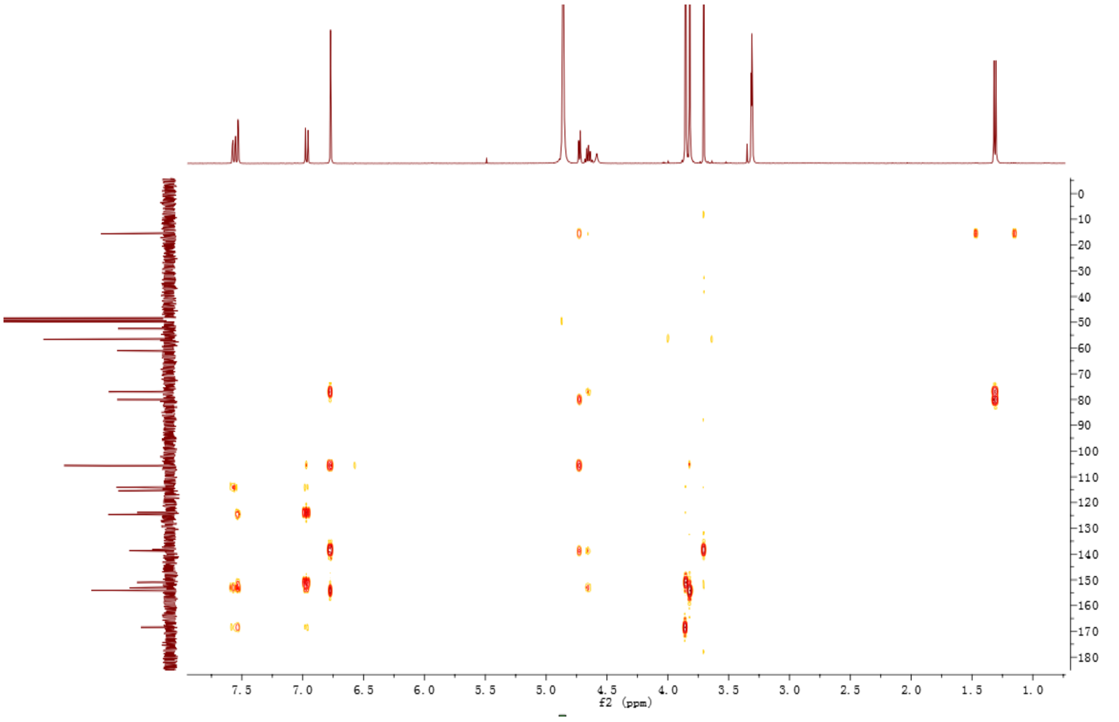

Figure S75. ^{1}H-^{1}H COSY spectrum of **8** in CD_3OD

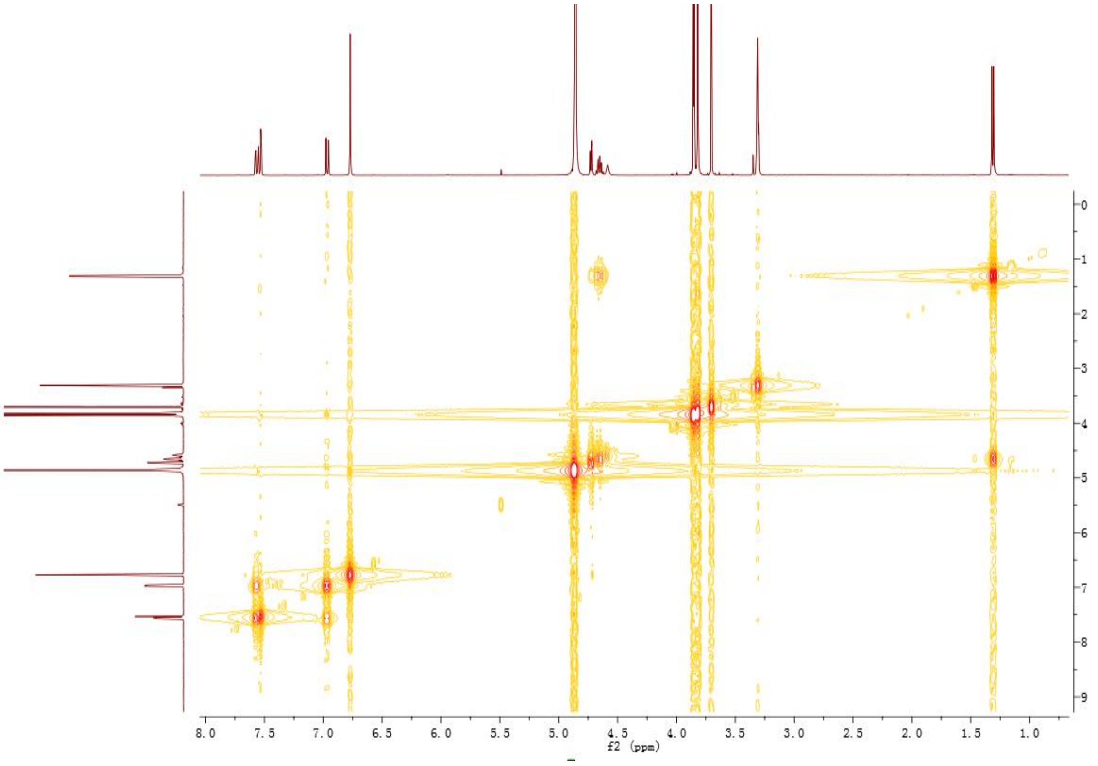

Figure S76. NOESY spectrum of **8** in CD_3OD

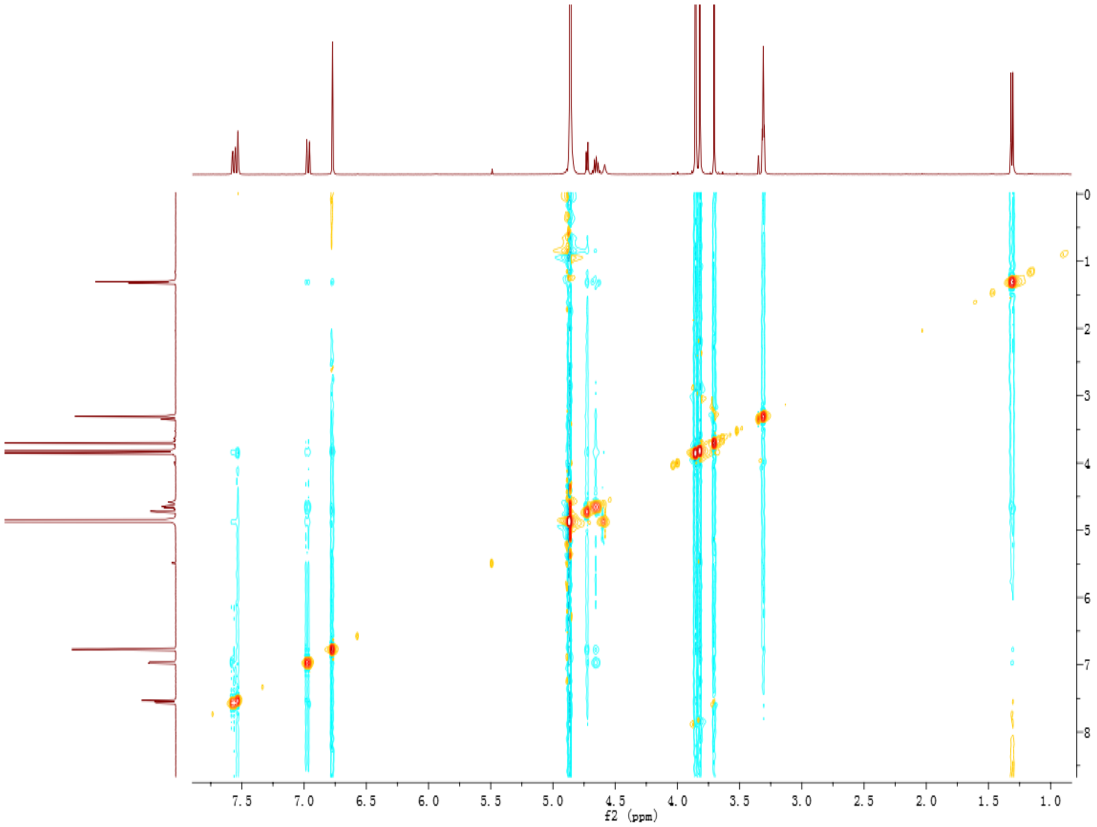

Figure S77. ECD spectrum of **8** in MeOH

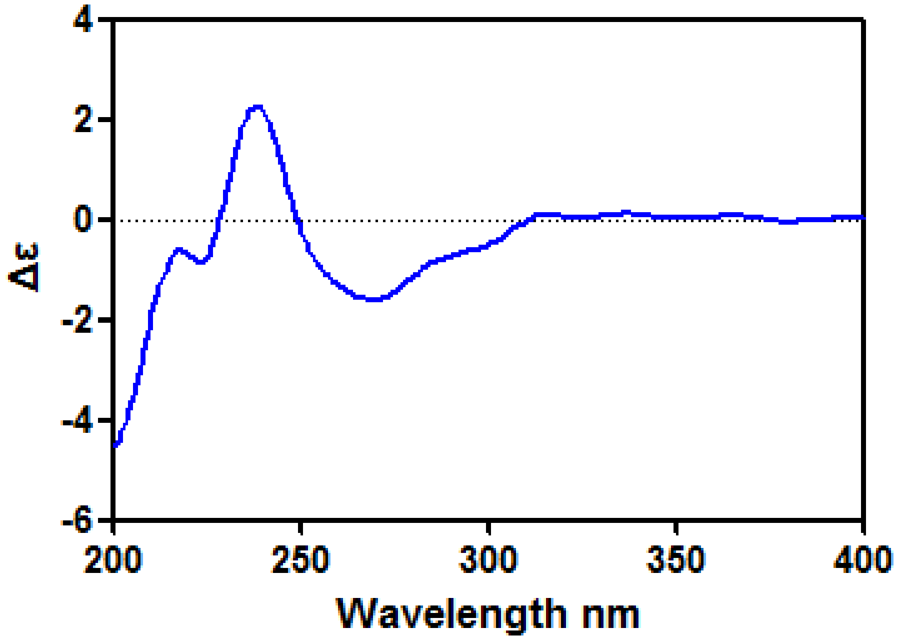

Figure S78. (+)-HR-ESI-MS spectrum of *S*-MTPA-**7** ester

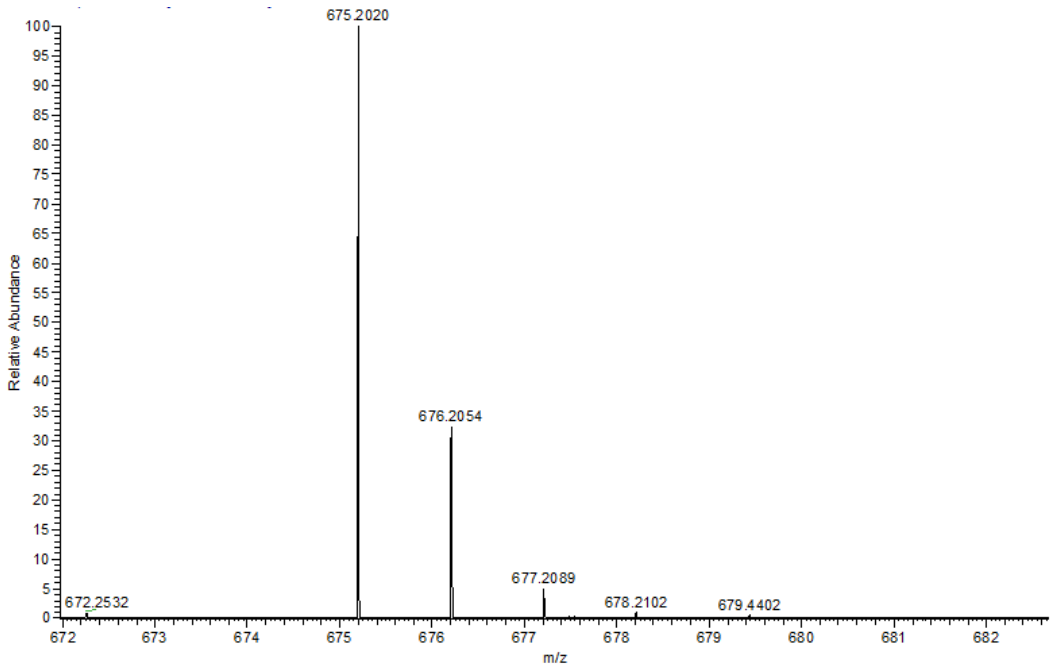

Figure S79. (+)-HR-ESI-MS spectrum of *R*-MTPA-**7** ester

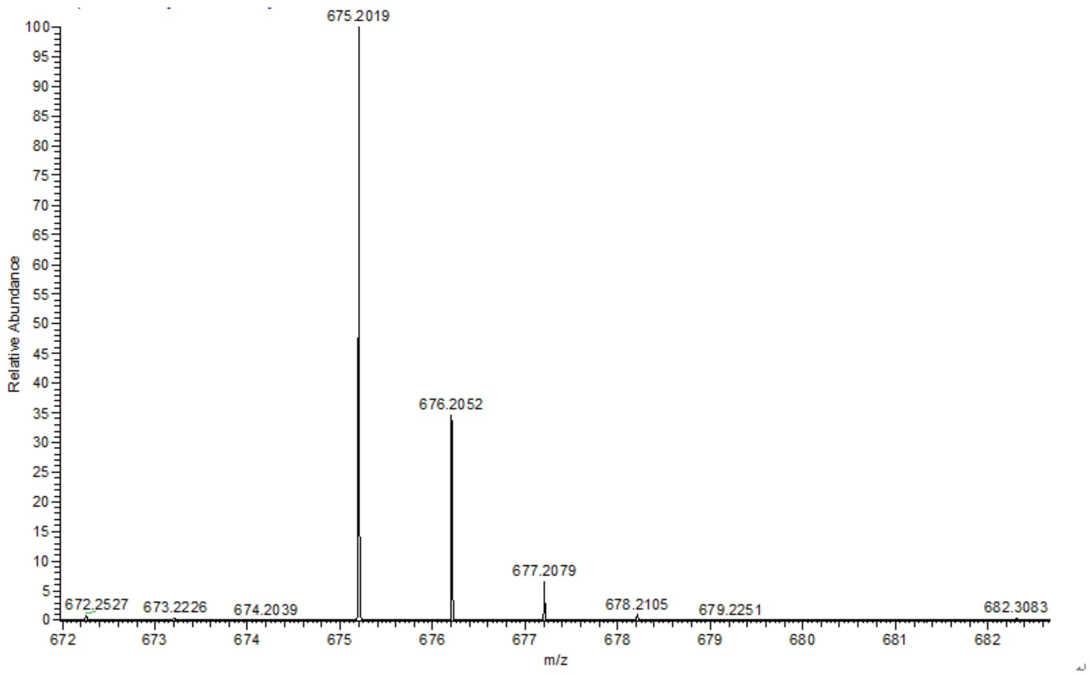

Figure S80. ^{1}H NMR spectrum of *R*/*S*-MTPA-**7** ester in CD_3OD

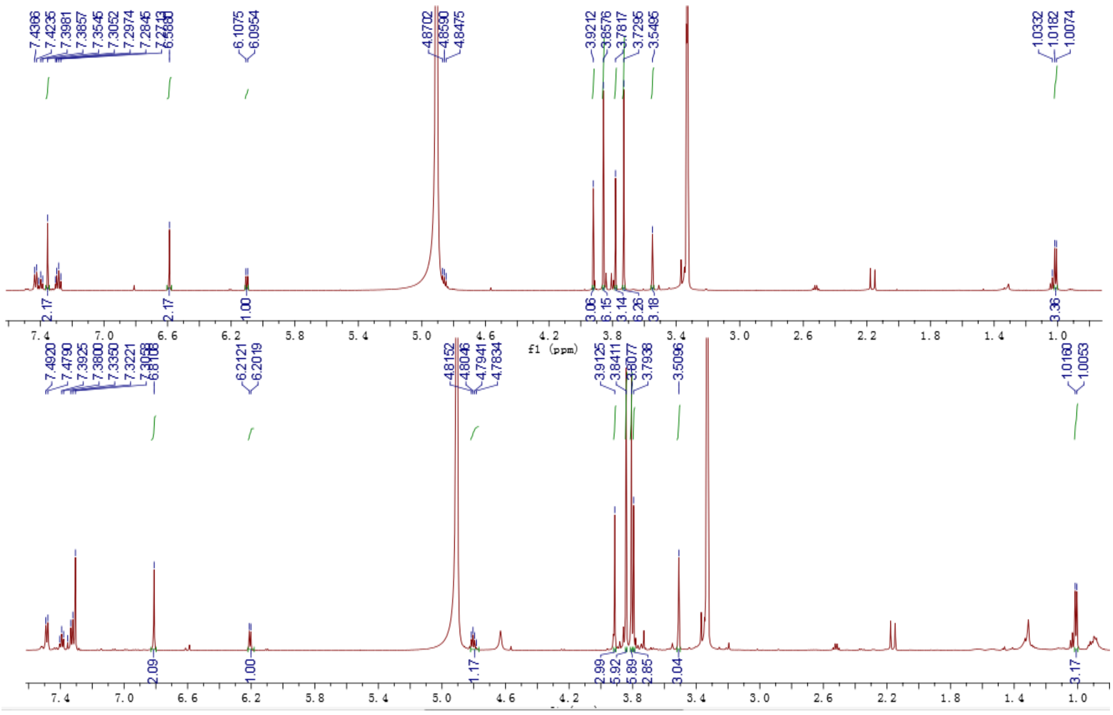

Figure S81. ECD spectra calculation

The conformational analyses were carried out for compounds **1a/1b** using BALLOON11 and confab12 programs. The theoretical calculation of ECD was performed using time-dependent density functional theory (TDDFT) at the B3LYP/6-31G (d, p) level in methanol with a PCM model. The calculated ECD curve was generated using SpecDis 1.51.

Figure S82. Five lowest energy conformers of the isomer 7*S*,7′*R*,8*S*,8′*S* and five lowest energy conformers of the 7*R*,7′*S*,8*R*,8′*R* isomer of compound **1**.

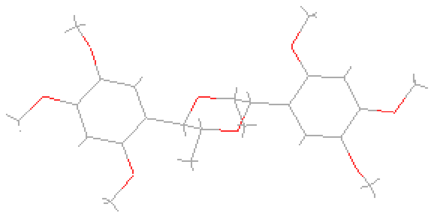

a

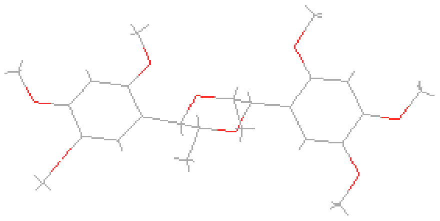

b

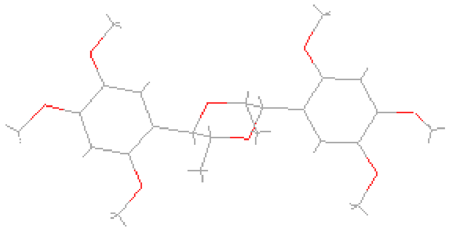

c

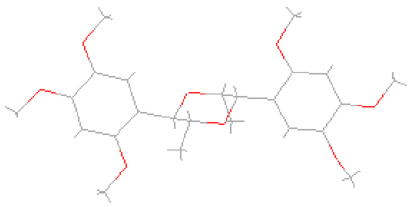

d

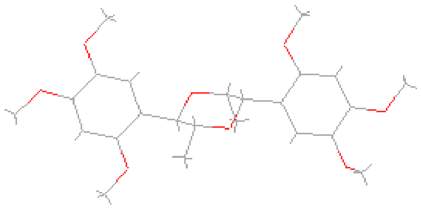

e

a-e: Five lowest energy conformers of the7*S*, 7′*R*, 8*S*, 8′*S* isomer.

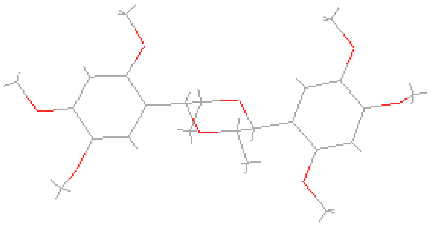

a′

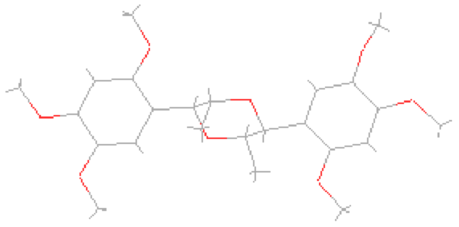

b′

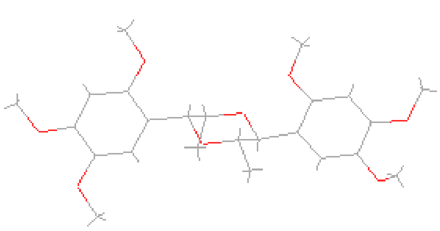

c′

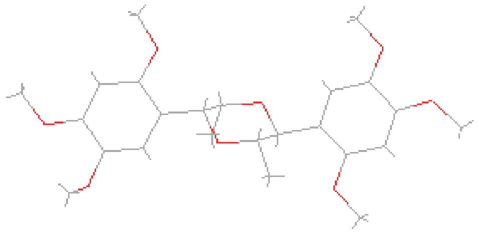

d′

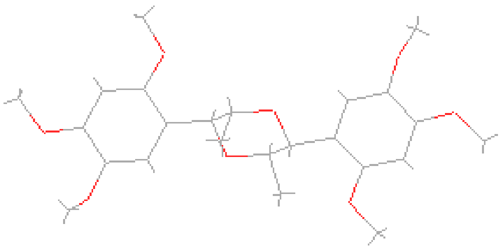

e′

a′-e′: Five lowest energy conformers of the 7*R*, 7′*S*, 8*R*, 8′*R* isomer.

Table S1. Relative free energies (ΔG) and equilibrium populations (P) of the conformers of the (7*S*,7′*R*,8*S*,8′*S*)-**1** and (7*R*,7′*S*,8*R*,8′*R*)-**1**

conformer	isomer 7*S*,7′*R*,8*S*,8′*S*		conformer	Isomer 7*R*,7′*S*,8*R*,8′*R*	
	ΔG (kcal/mol)	P (%)		ΔG (kcal/mol)	P (%)
a	0.7975	9.96%	a′	1.4749	3.98%
b	1.5650	2.73%	b′	0.0000	47.89%
c	0.6379	13.05%	c′	1. 4666	4.03%
d	0.0000	38.27%	d′	0.2589	30.94%
e	0.0365	35.99%	e′	0.7650	13.17%
