# Supplementary material for: Antioxidant Lignans and Neolignans from Acorus tatarinowii
Source: Sci Rep. 2016 Mar 10;6:22909. doi: 10.1038/srep22909 (PMC4785495; doi:10.1038/srep22909)

checkCIF/PLATON report

You have not supplied any structure factors. As a result the full set of tests cannot be run.

THIS REPORT IS FOR GUIDANCE ONLY. IF USED AS PART OF A REVIEW PROCEDURE FOR PUBLICATION, IT SHOULD NOT REPLACE THE EXPERTISE OF AN EXPERIENCED CRYSTALLOGRAPHIC REFEREE.

No syntax errors found. CIF dictionary Interpreting this report

Datablock: cu_zyh_lyy1572_0m

```
Bond precision:  C-C = 0.0030 A          Wavelength=1.54178

Cell:            a=9.8071(4)     b=7.9422(3)        c=13.6711(5)
                 alpha=90        beta=99.039(1)     gamma=90
Temperature:     100 K

                Calculated                 Reported
Volume          1051.62(7)                 1051.62(7)
Space group     P 21                       P 21
Hall group      P 2yb                      P 2yb
Moiety formula  C23 H28 O7                 C23 H28 O7
Sum formula     C23 H28 O7                 C23 H28 O7
Mr              416.45                     416.45
Dx,g cm-3       1.315                      1.315
Z               2                          2
Mu (mm-1)       0.801                      0.801
F000            444.0                      444.0
F000'           445.47
h,k,lmax        11,9,16                    11,9,16
Nref            3960[ 2131]                3314
Tmin,Tmax       0.436,0.548                0.391,0.585
Tmin'           0.274

Correction method= # Reported T Limits: Tmin=0.391 Tmax=0.585
AbsCorr = MULTI-SCAN

Data completeness= 1.56/0.84      Theta(max)= 69.600

R(reflections)= 0.0338( 3313)     wR2(reflections)= 0.0983( 3314)

S = 1.094                  Npar= 278
```

The following ALERTS were generated. Each ALERT has the format
test-name_ALERT_alert-type_alert-level.
Click on the hyperlinks for more details of the test.

Alert level A

```
PLAT029_ALERT_3_A _diffrn_measured_fraction_theta_full Low .......      0.920 Note
```

Alert level B

```
CRYSS02_ALERT_3_B  The value of _exptl_crystal_size_min is > 0.6
   Minimum crystal size given =   0.750
CRYSS02_ALERT_3_B  The value of _exptl_crystal_size_mid is > 0.8
   Mid crystal size given     =   1.010
CRYSS02_ALERT_3_B  The value of _exptl_crystal_size_max is > 1.0
   Maximum crystal size given =   1.440
```

Alert level G

```
PLAT005_ALERT_5_G No _iucr_refine_instructions_details  in the CIF     Please Do !
PLAT063_ALERT_4_G Crystal Size Likely too Large for Beam Size ....       1.44 mm
PLAT720_ALERT_4_G Number of Unusual/Non-Standard Labels ..........          2 Note
PLAT791_ALERT_4_G The Model has Chirality at C7      (Chiral SPGR)          R Verify
PLAT791_ALERT_4_G The Model has Chirality at C8      (Chiral SPGR)          R Verify
PLAT791_ALERT_4_G The Model has Chirality at C8'     (Chiral SPGR)          R Verify
PLAT899_ALERT_4_G SHELXL97   is Deprecated and Succeeded by SHELXL       2014 Note
```

1 **ALERT level A** = Most likely a serious problem - resolve or explain
3 **ALERT level B** = A potentially serious problem, consider carefully
0 **ALERT level C** = Check. Ensure it is not caused by an omission or oversight
7 **ALERT level G** = General information/check it is not something unexpected

0 ALERT type 1 CIF construction/syntax error, inconsistent or missing data
0 ALERT type 2 Indicator that the structure model may be wrong or deficient
4 ALERT type 3 Indicator that the structure quality may be low
6 ALERT type 4 Improvement, methodology, query or suggestion
1 ALERT type 5 Informative message, check

It is advisable to attempt to resolve as many as possible of the alerts in all categories. Often the minor alerts point to easily fixed oversights, errors and omissions in your CIF or refinement strategy, so attention to these fine details can be worthwhile. In order to resolve some of the more serious problems it may be necessary to carry out additional measurements or structure refinements. However, the purpose of your study may justify the reported deviations and the more serious of these should normally be commented upon in the discussion or experimental section of a paper or in the "special_details" fields of the CIF. checkCIF was carefully designed to identify outliers and unusual parameters, but every test has its limitations and alerts that are not important in a particular case may appear. Conversely, the absence of alerts does not guarantee there are no aspects of the results needing attention. It is up to the individual to critically assess their own results and, if necessary, seek expert advice.

Publication of your CIF in IUCr journals

A basic structural check has been run on your CIF. These basic checks will be run on all CIFs submitted for publication in IUCr journals (*Acta Crystallographica*, *Journal of Applied Crystallography*, *Journal of Synchrotron Radiation*); however, if you intend to submit to *Acta Crystallographica Section C* or *E*, you should make sure that full publication checks are run on the final version of your CIF prior to submission.

Publication of your CIF in other journals

Please refer to the *Notes for Authors* of the relevant journal for any special instructions relating to CIF submission.

Validation response form

Please find below a validation response form (VRF) that can be filled in and pasted into your CIF.

```
# start Validation Reply Form
_vrf_PLAT029_cu_zyh_lyy1572_0m
;
PROBLEM: _diffrn_measured_fraction_theta_full Low .......      0.920 Note
RESPONSE: ...
;
# end Validation Reply Form
```

PLATON version of 21/06/2015; check.def file version of 21/06/2015

Datablock cu_zyh_lyy1572_0m - ellipsoid plot

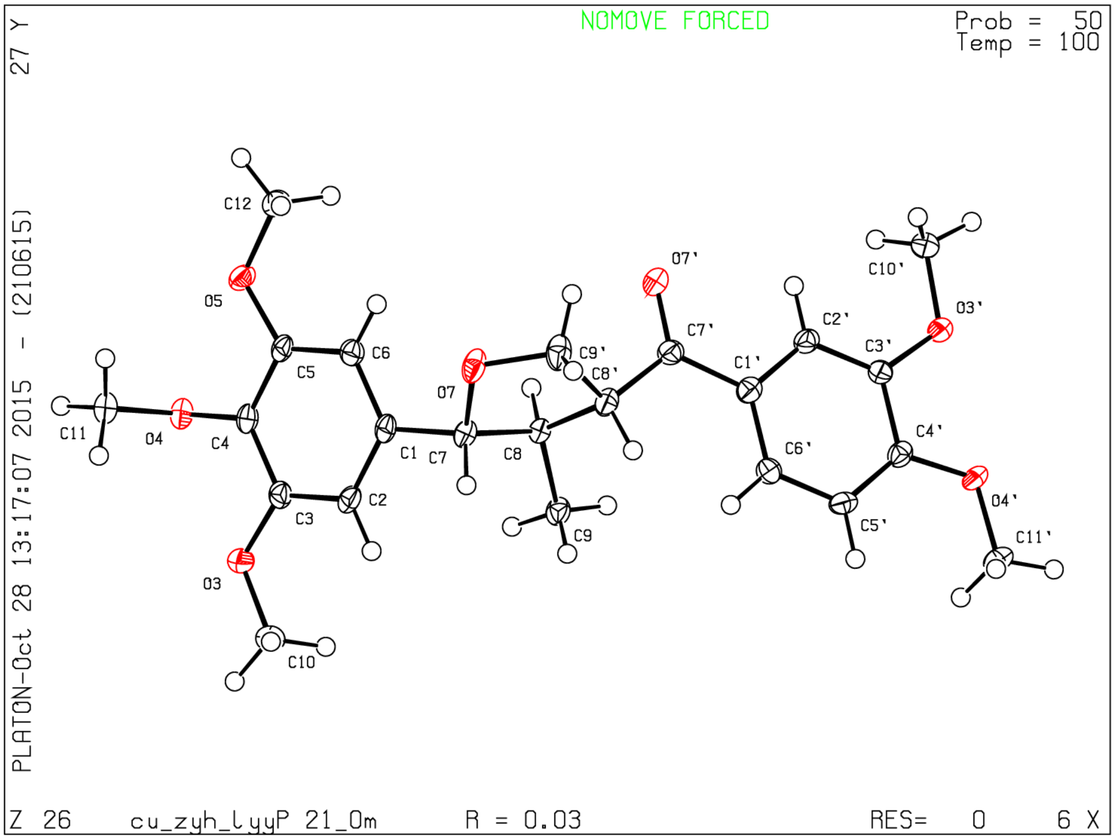

Supplement: Supplementary checkcif 4 [file srep22909-s3.pdf]
